# Supplementary figures and images for: The integrated stress response drives MET oncogene overexpression in cancers
Source: EMBO J. 2025 Jan 7;44(4):1107–30. doi: 10.1038/s44318-024-00338-4 (PMC11832788; doi:10.1038/s44318-024-00338-4)

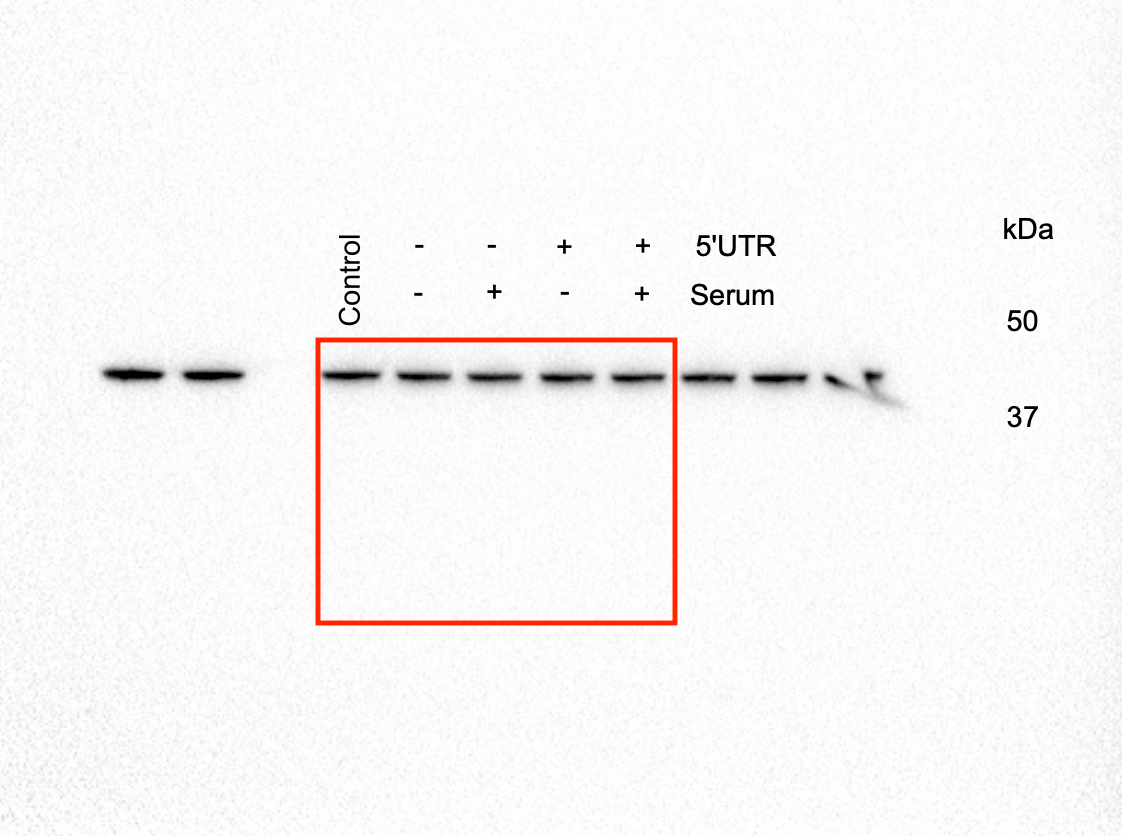

Supplement: Supplementary file 2 — Source data Fig. 1 [file 44318_2024_338_MOESM2_ESM.zip › SD figure 1/1F/TBP WB.tif]

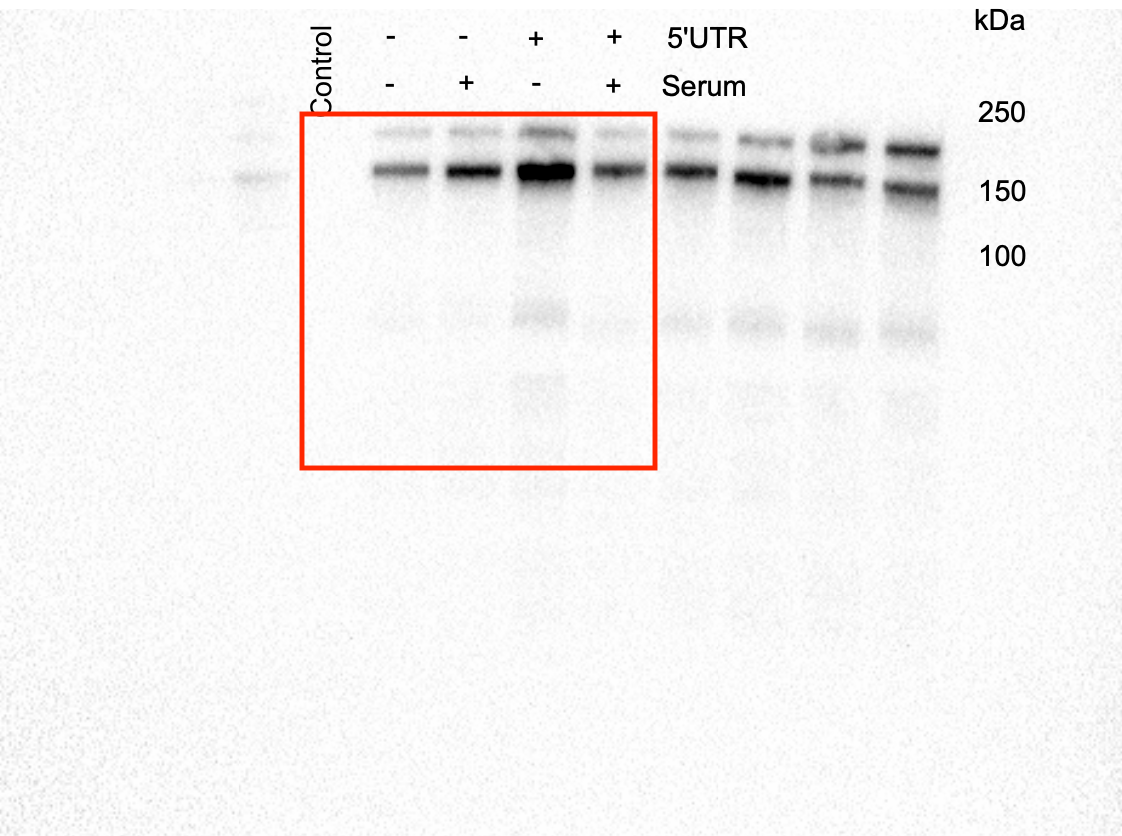

Supplement: Supplementary file 2 — Source data Fig. 1 [file 44318_2024_338_MOESM2_ESM.zip › SD figure 1/1F/MET WB.tif]

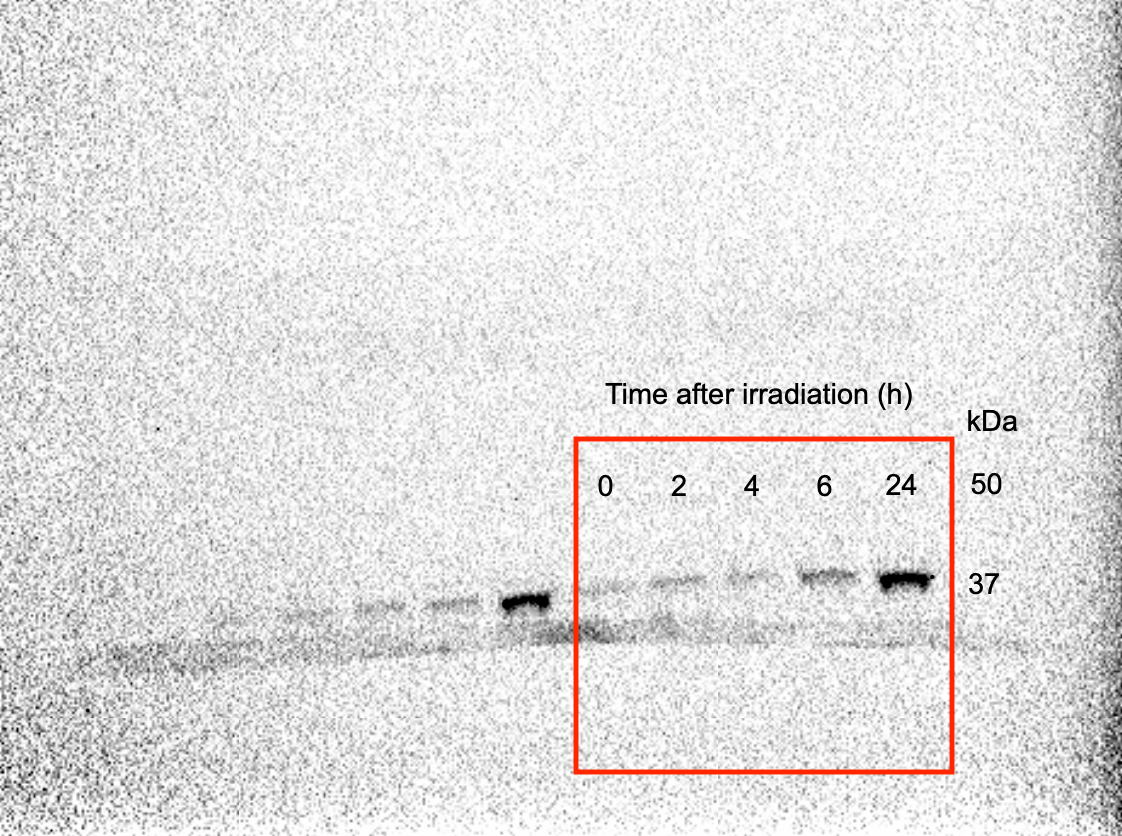

Supplement: Supplementary file 2 — Source data Fig. 1 [file 44318_2024_338_MOESM2_ESM.zip › SD figure 1/1E/eIF2a-P WB Irradiation.tif]

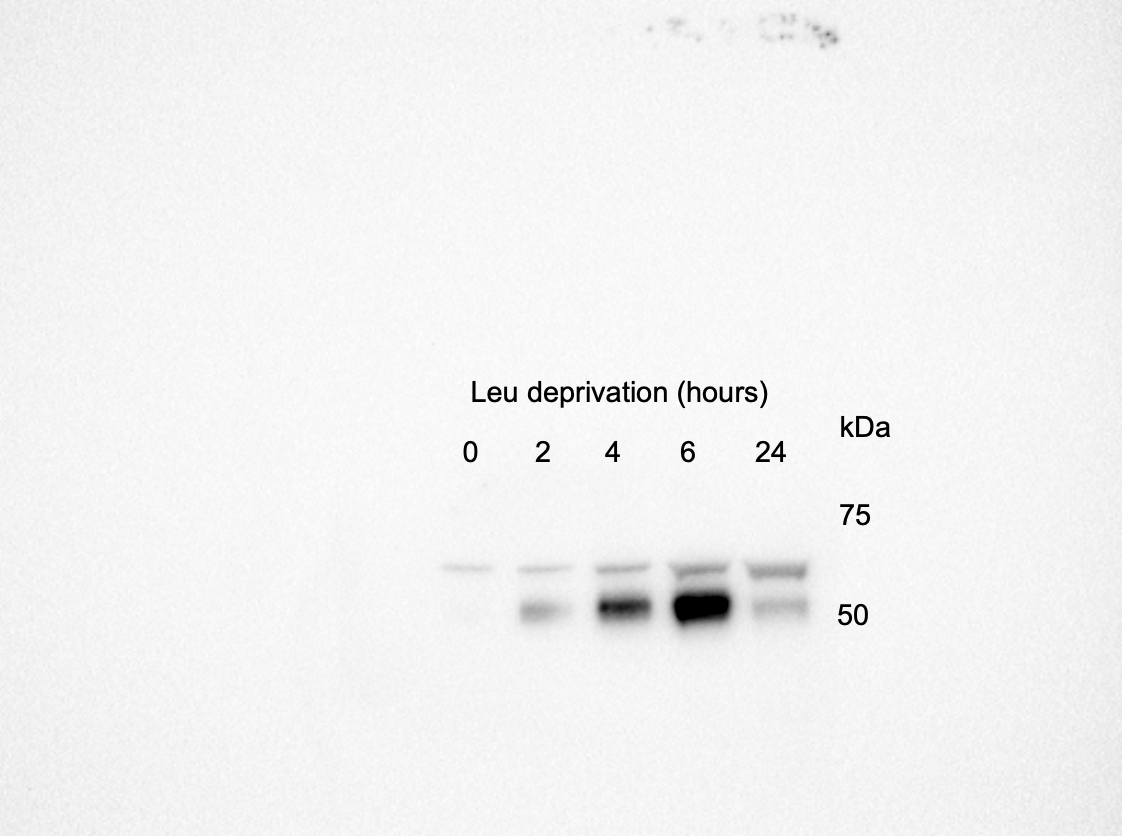

Supplement: Supplementary file 2 — Source data Fig. 1 [file 44318_2024_338_MOESM2_ESM.zip › SD figure 1/1E/ATF4 WB Leucine deprivation.tif]

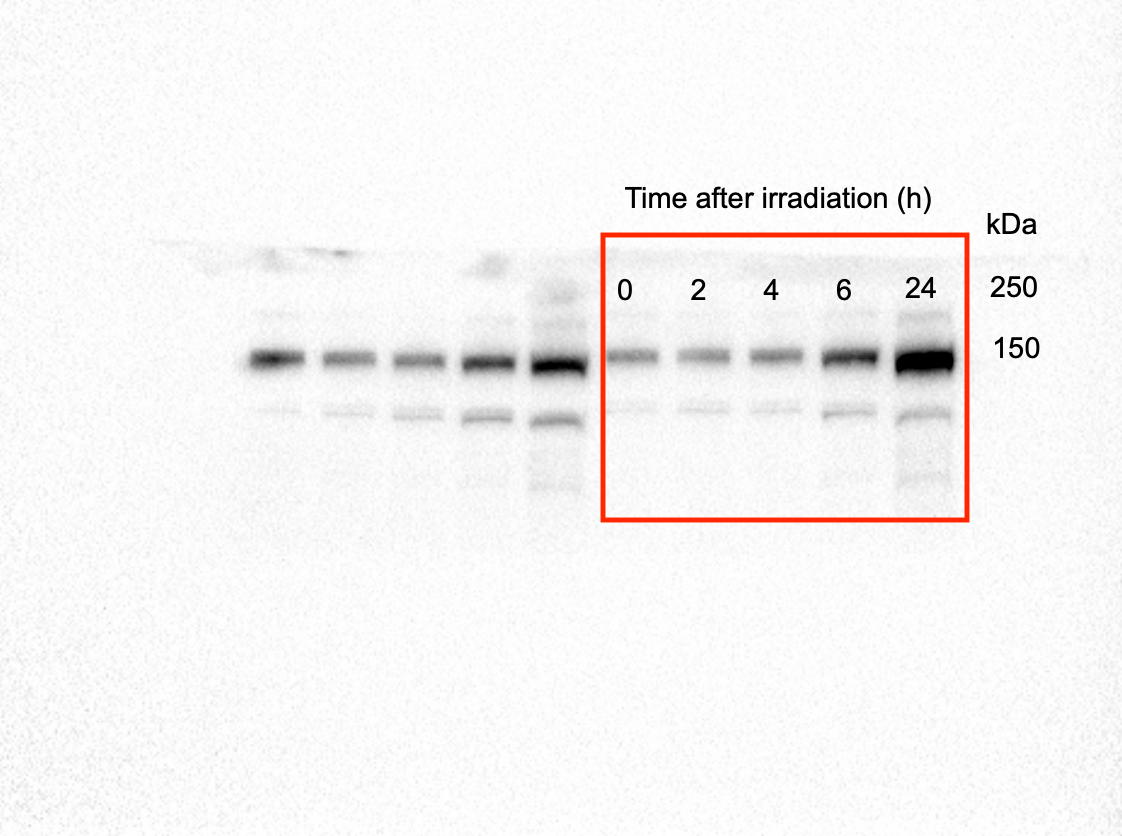

Supplement: Supplementary file 2 — Source data Fig. 1 [file 44318_2024_338_MOESM2_ESM.zip › SD figure 1/1E/MET WB Irradiation.tif]

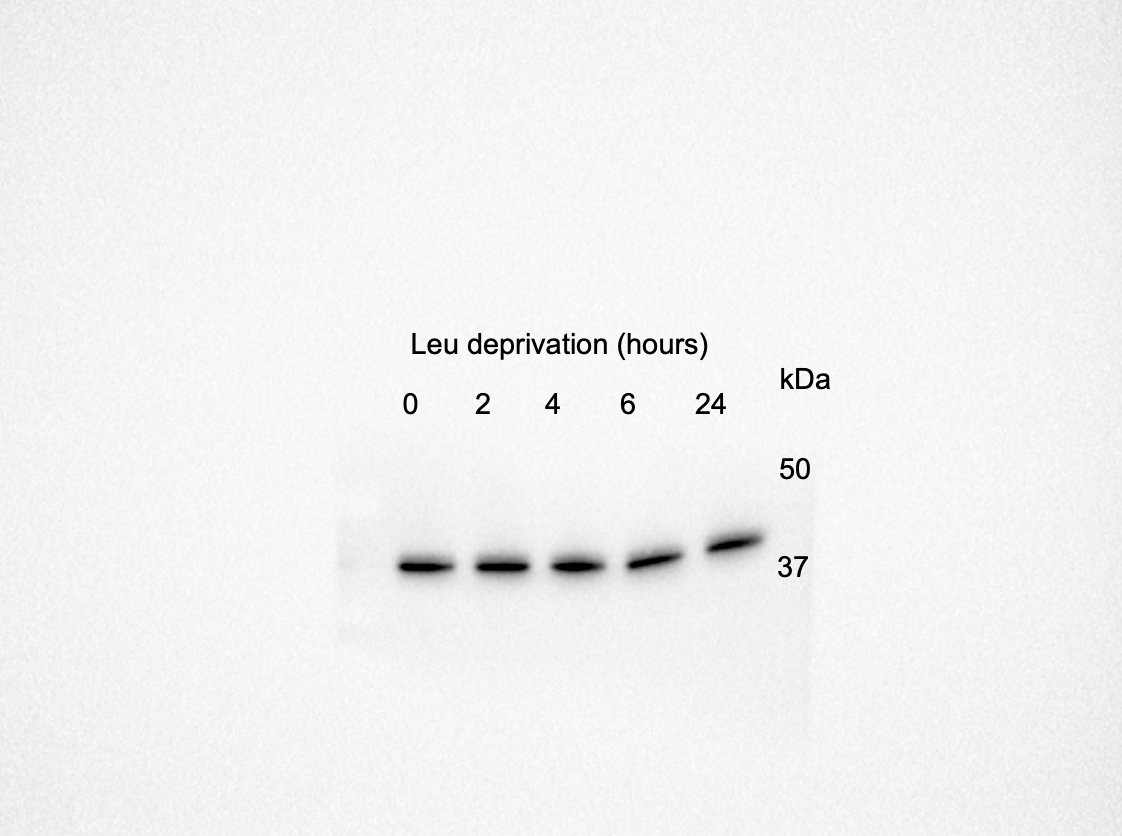

Supplement: Supplementary file 2 — Source data Fig. 1 [file 44318_2024_338_MOESM2_ESM.zip › SD figure 1/1E/TBP WB Leu deprivation .tif]

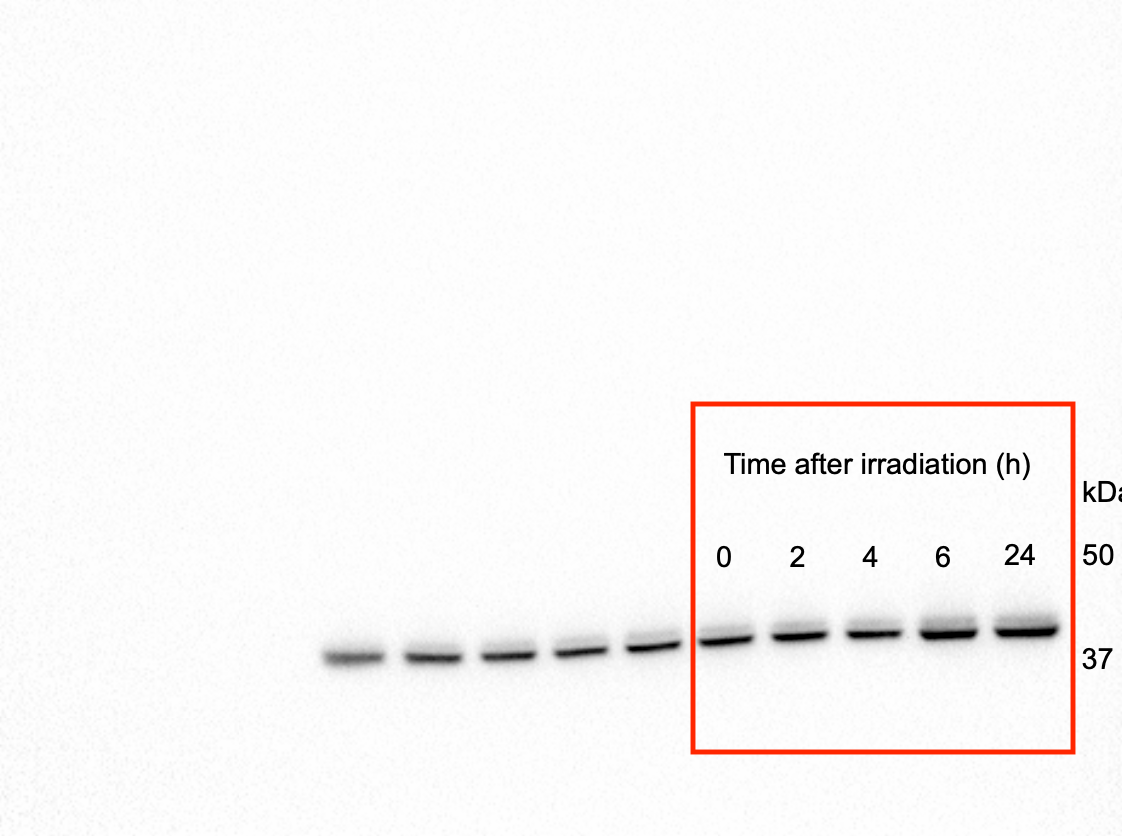

Supplement: Supplementary file 2 — Source data Fig. 1 [file 44318_2024_338_MOESM2_ESM.zip › SD figure 1/1E/TBP WB Irradiation.tif]

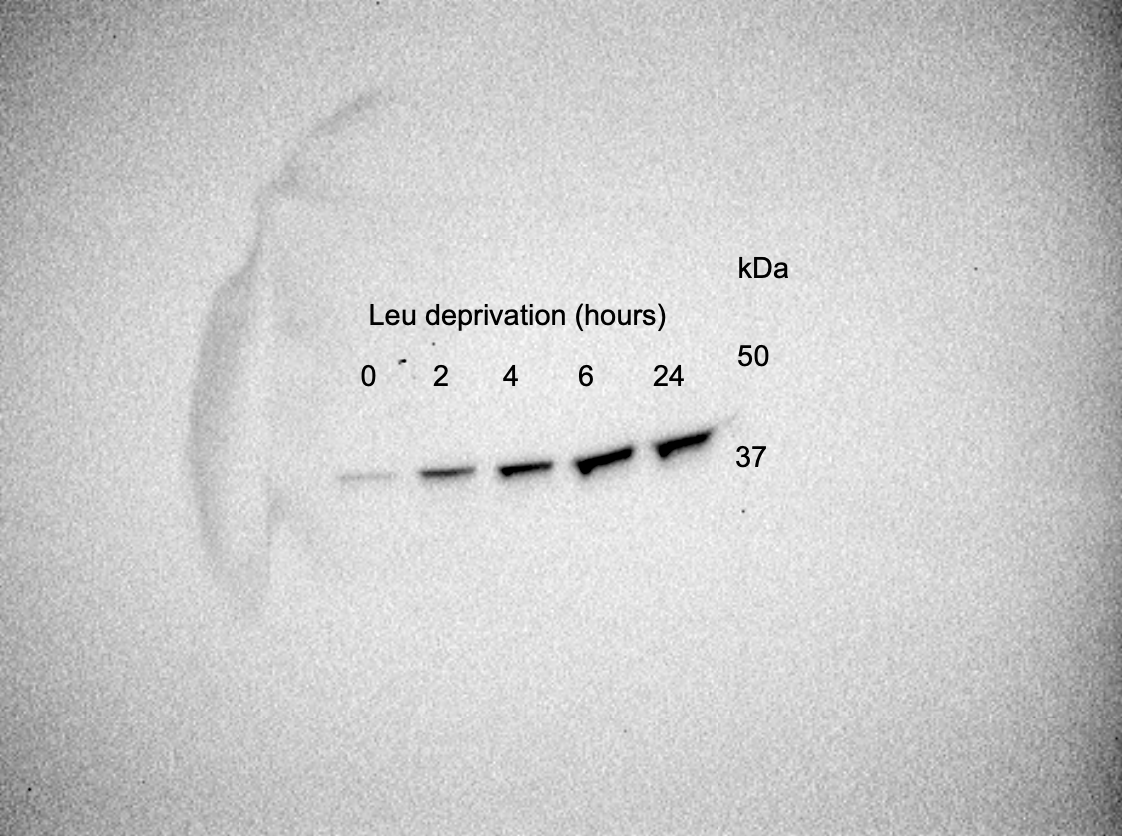

Supplement: Supplementary file 2 — Source data Fig. 1 [file 44318_2024_338_MOESM2_ESM.zip › SD figure 1/1E/eIF2a-P WB Leu deprivation .tif]

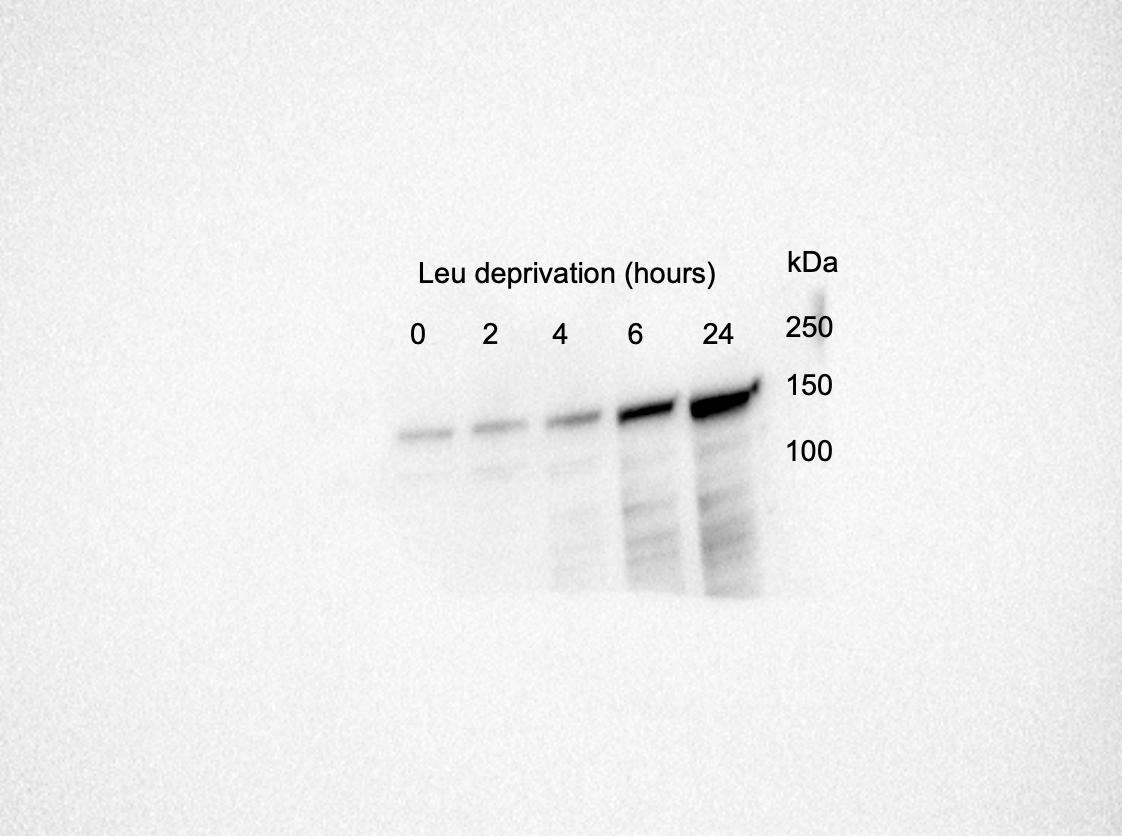

Supplement: Supplementary file 2 — Source data Fig. 1 [file 44318_2024_338_MOESM2_ESM.zip › SD figure 1/1E/MET WB Leu deprivation.tif]

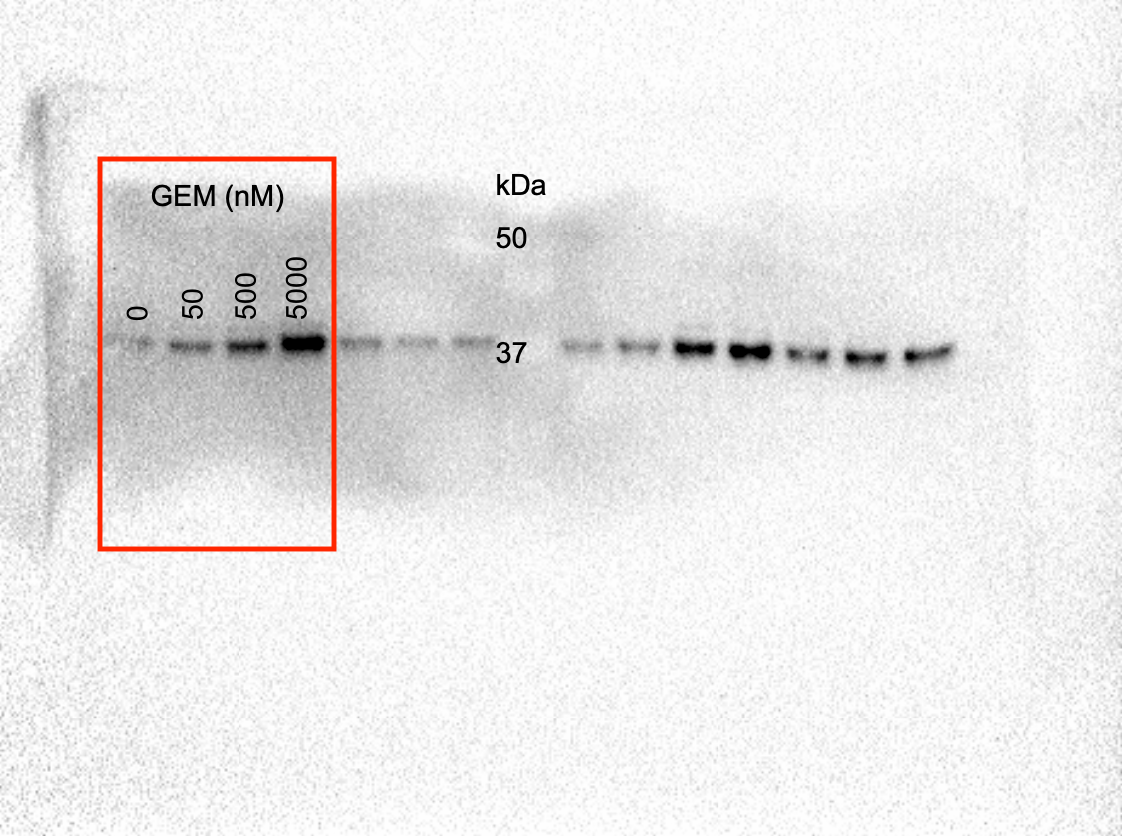

Supplement: Supplementary file 2 — Source data Fig. 1 [file 44318_2024_338_MOESM2_ESM.zip › SD figure 1/1E/eIF2a-P WB GEM.tif]

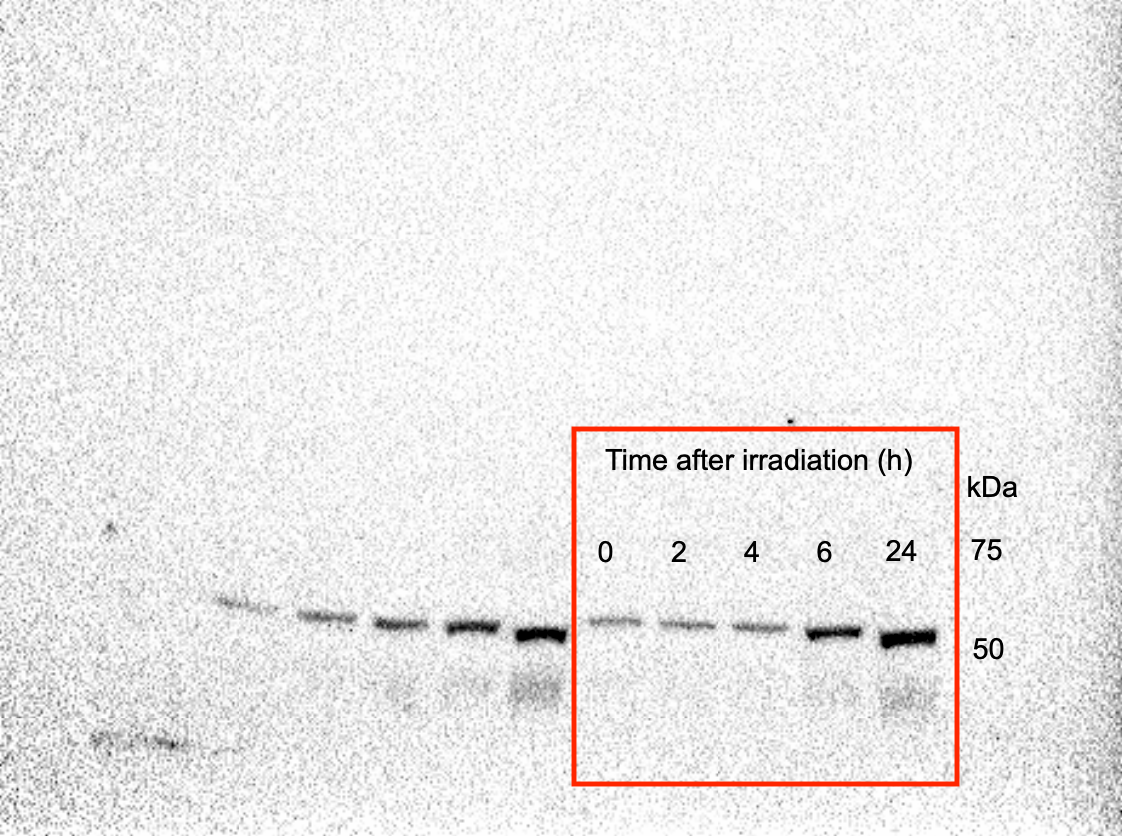

Supplement: Supplementary file 2 — Source data Fig. 1 [file 44318_2024_338_MOESM2_ESM.zip › SD figure 1/1E/ATF4 WB Irradiation.tif]

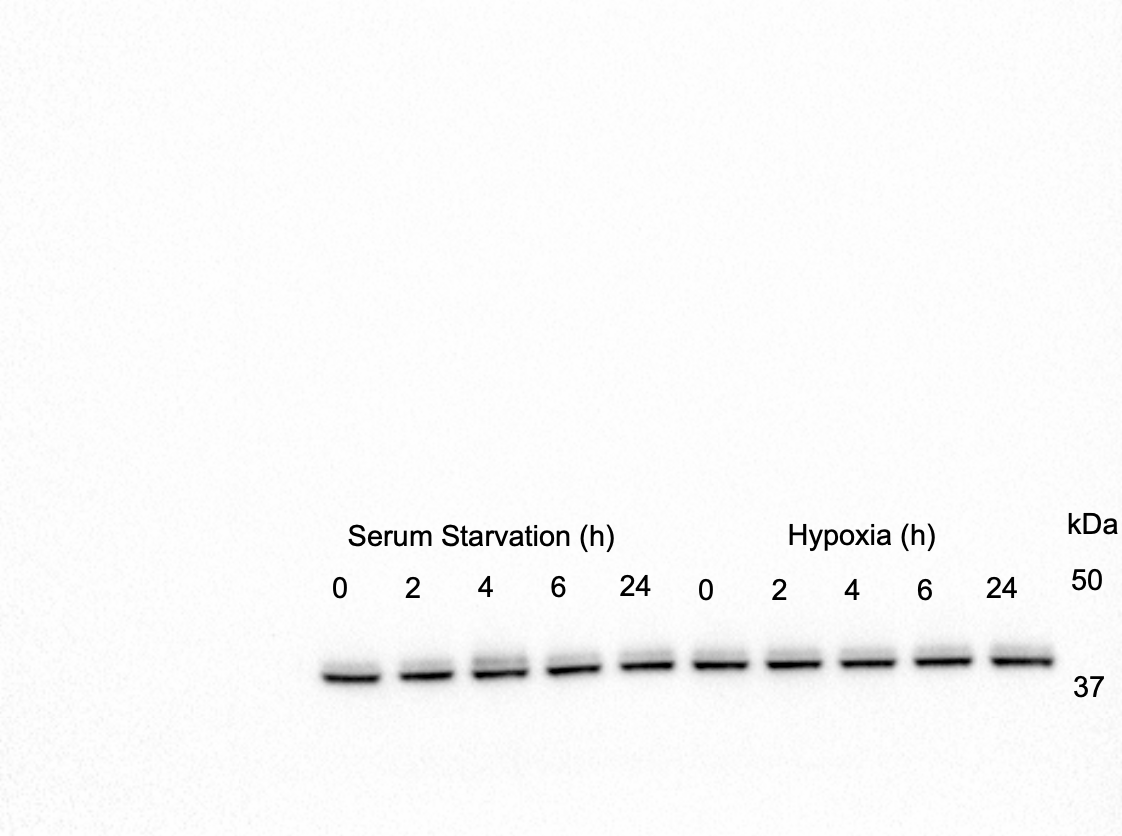

Supplement: Supplementary file 2 — Source data Fig. 1 [file 44318_2024_338_MOESM2_ESM.zip › SD figure 1/1E/TBP WB SS Hypoxia.tif]

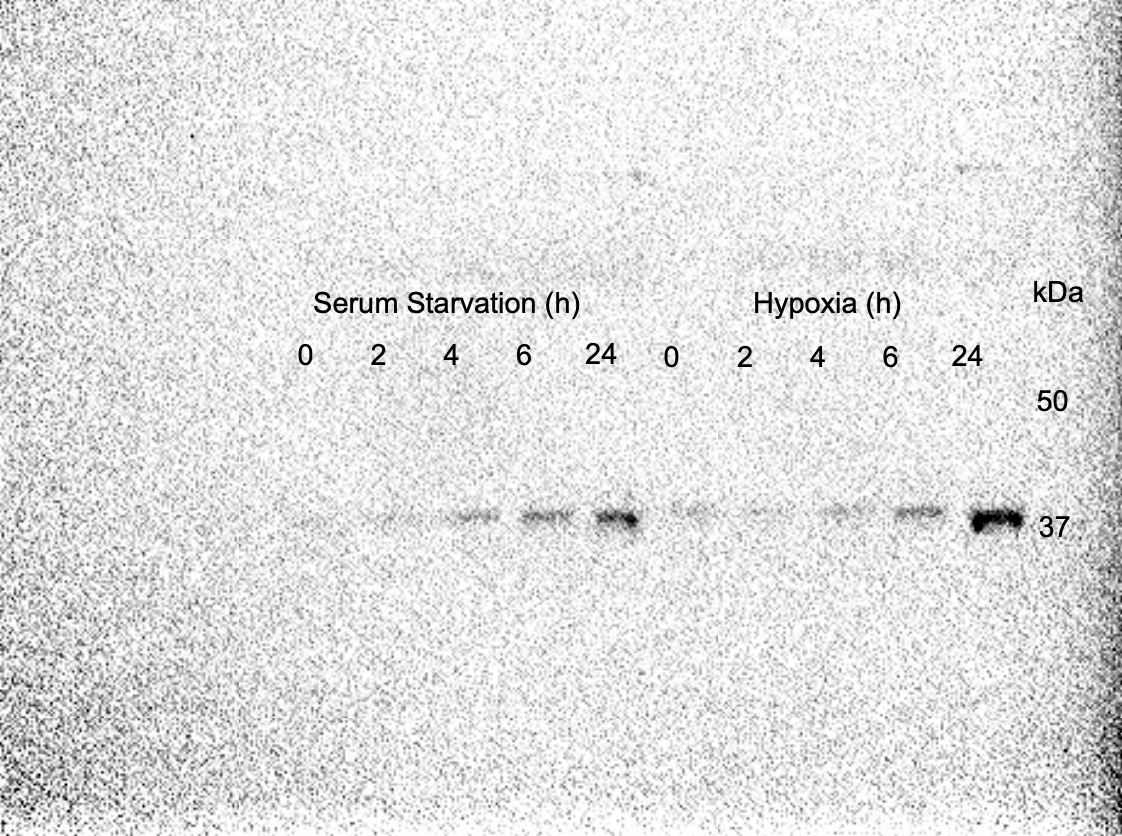

Supplement: Supplementary file 2 — Source data Fig. 1 [file 44318_2024_338_MOESM2_ESM.zip › SD figure 1/1E/eIF2a-P WB SS Hypoxia.tif]

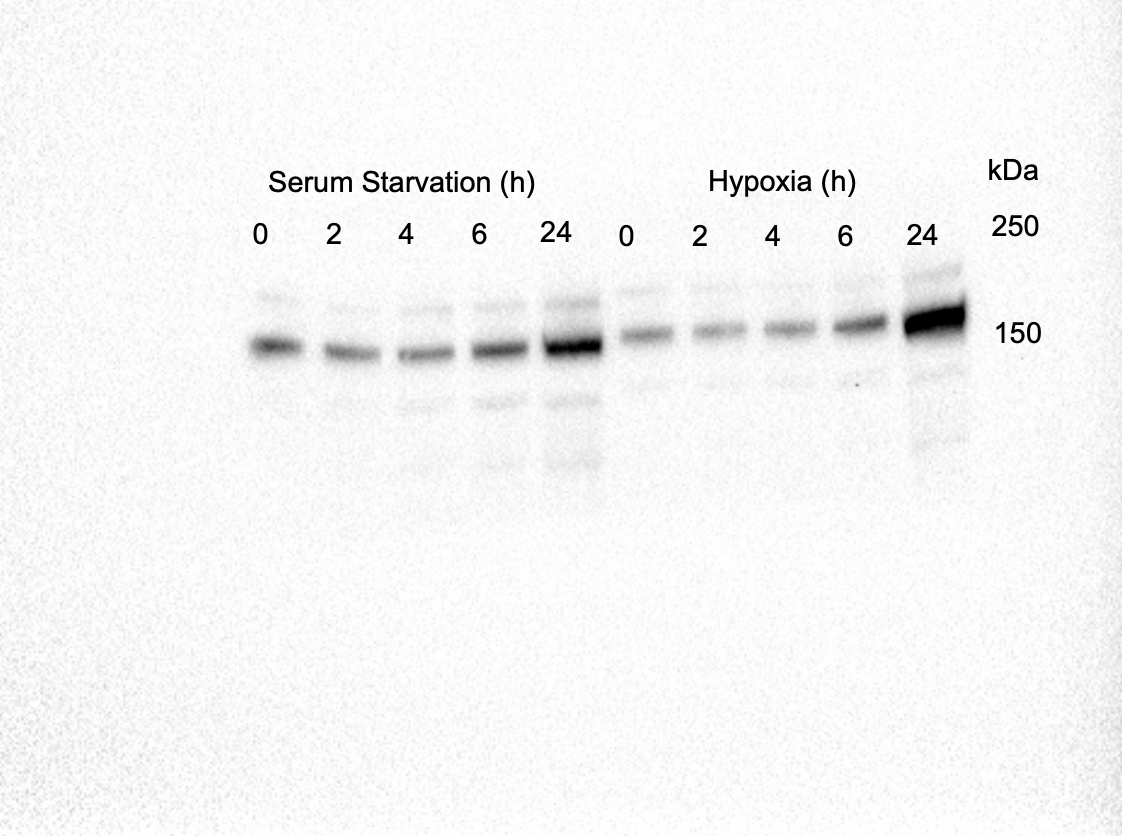

Supplement: Supplementary file 2 — Source data Fig. 1 [file 44318_2024_338_MOESM2_ESM.zip › SD figure 1/1E/MET WB SS Hypoxia.tif]

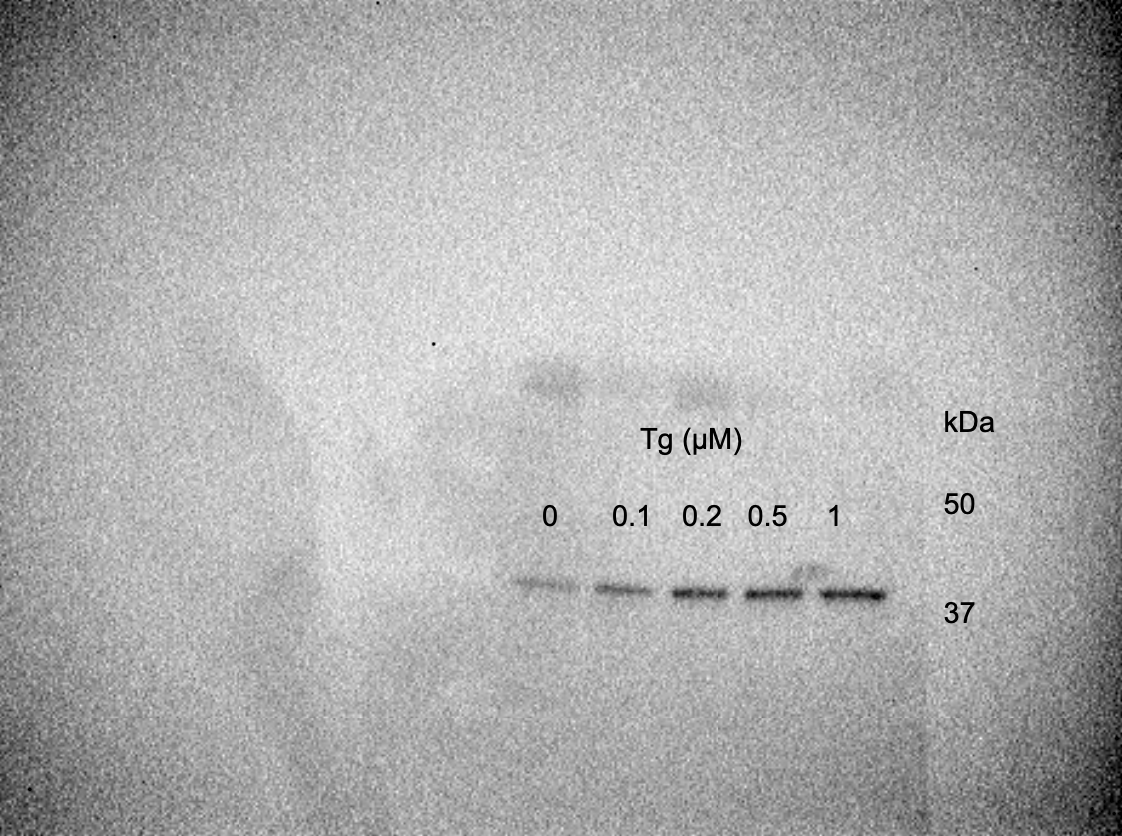

Supplement: Supplementary file 2 — Source data Fig. 1 [file 44318_2024_338_MOESM2_ESM.zip › SD figure 1/1E/eIF2a-P WB Thapsigargin .tif]

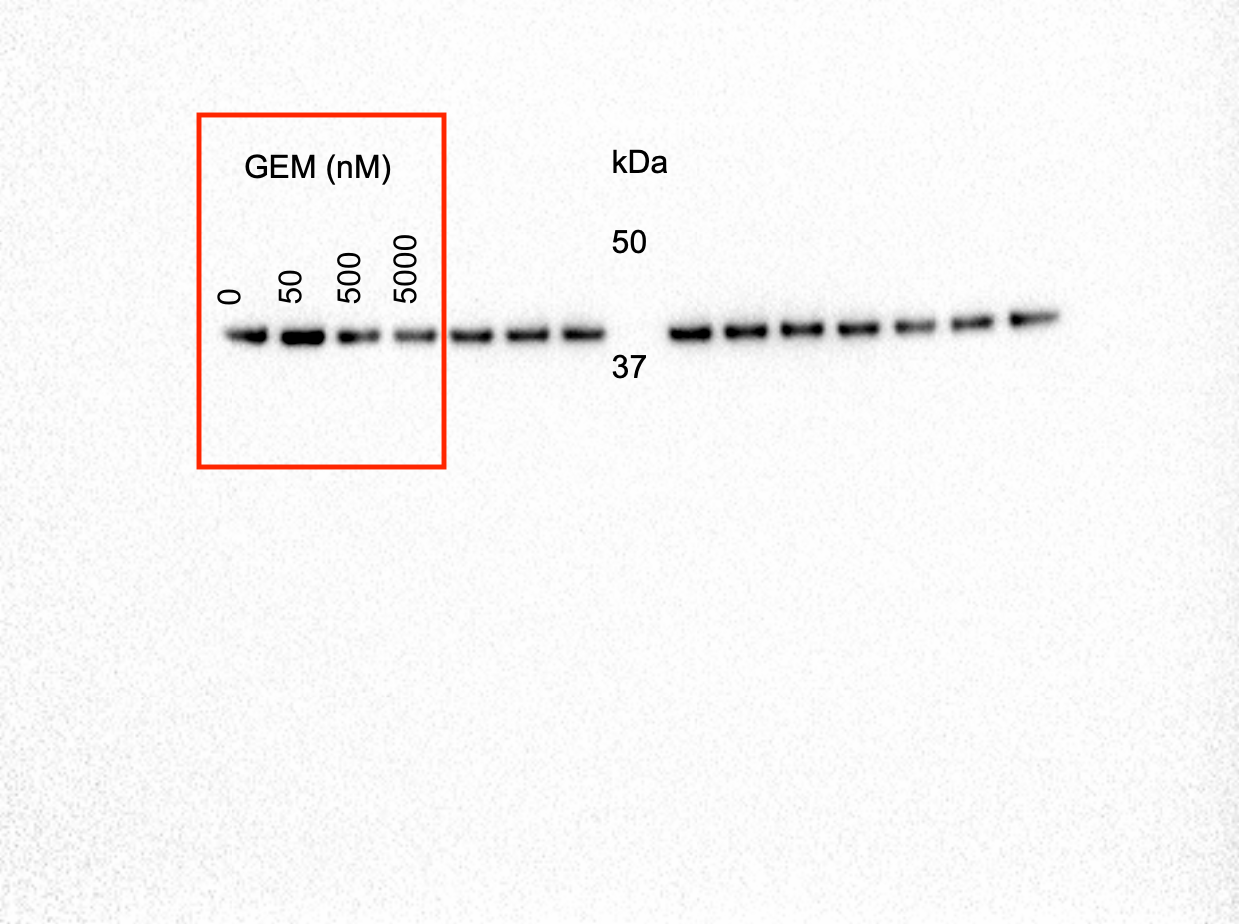

Supplement: Supplementary file 2 — Source data Fig. 1 [file 44318_2024_338_MOESM2_ESM.zip › SD figure 1/1E/TBP WB GEM.tif]

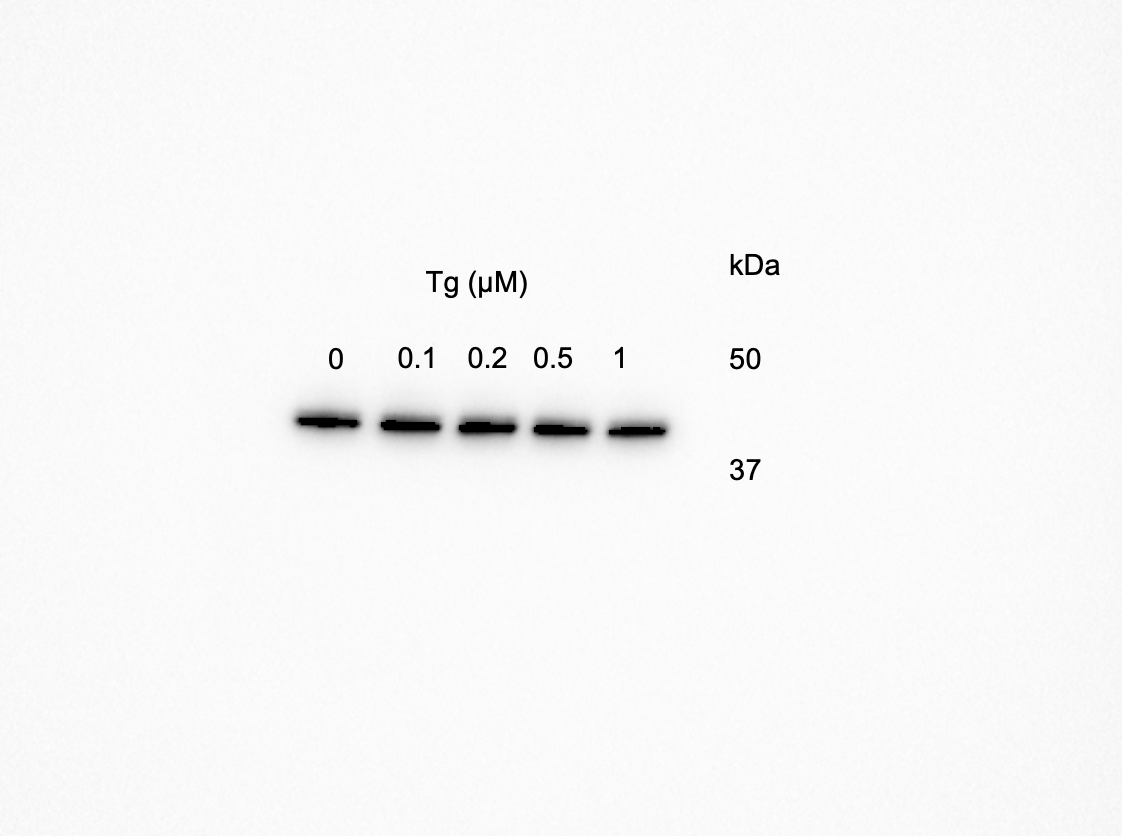

Supplement: Supplementary file 2 — Source data Fig. 1 [file 44318_2024_338_MOESM2_ESM.zip › SD figure 1/1E/TBP WB Thapsigargin .tif]

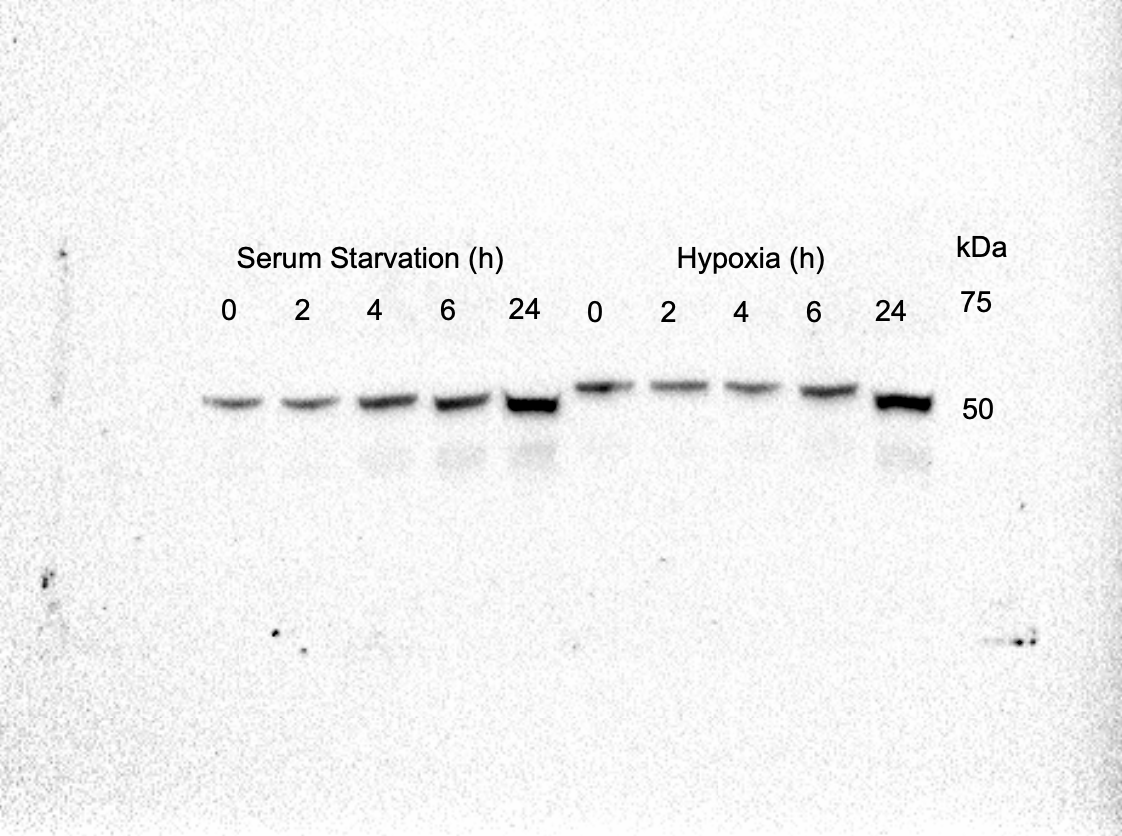

Supplement: Supplementary file 2 — Source data Fig. 1 [file 44318_2024_338_MOESM2_ESM.zip › SD figure 1/1E/ATF4 WB SS Hypoxia.tif]

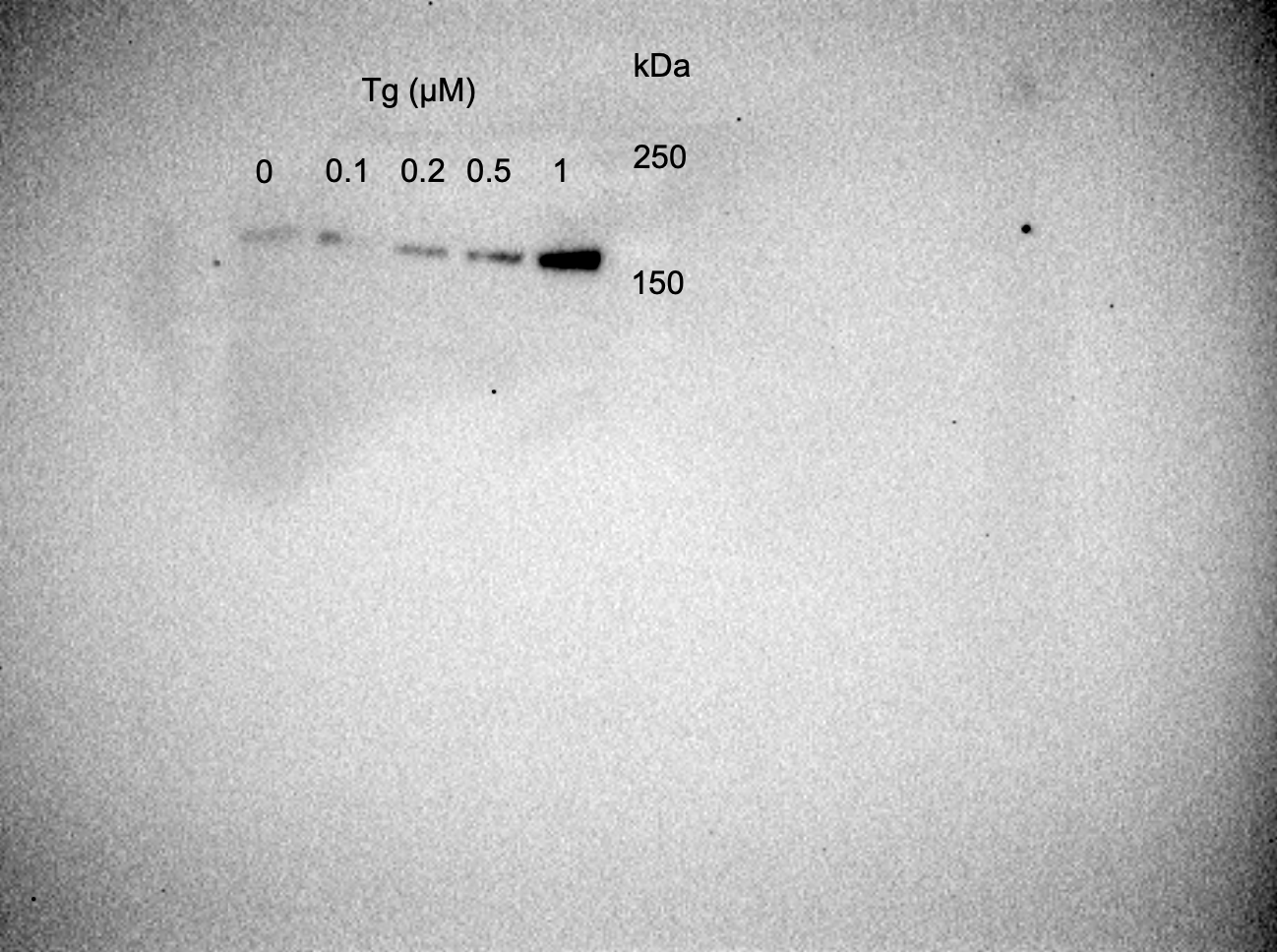

Supplement: Supplementary file 2 — Source data Fig. 1 [file 44318_2024_338_MOESM2_ESM.zip › SD figure 1/1E/MET WB Thapsigargin .tif]

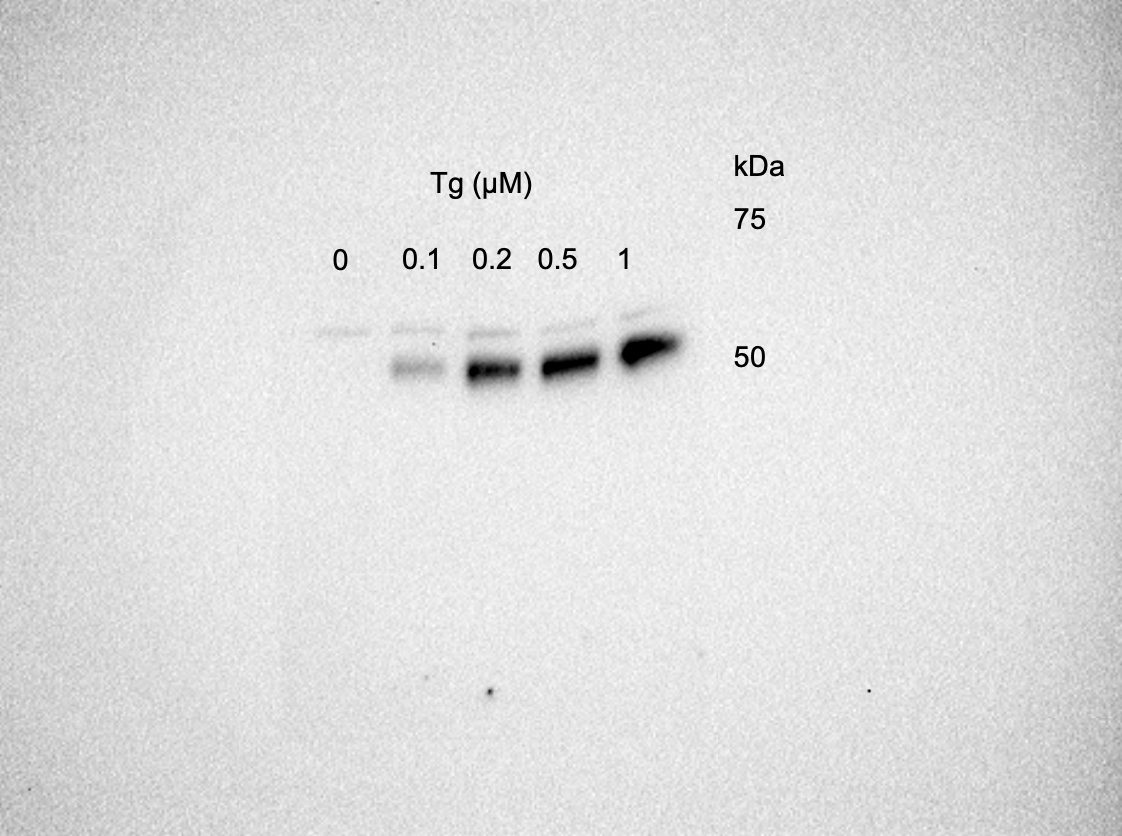

Supplement: Supplementary file 2 — Source data Fig. 1 [file 44318_2024_338_MOESM2_ESM.zip › SD figure 1/1E/ATF4 WB Thapsigargin .tif]

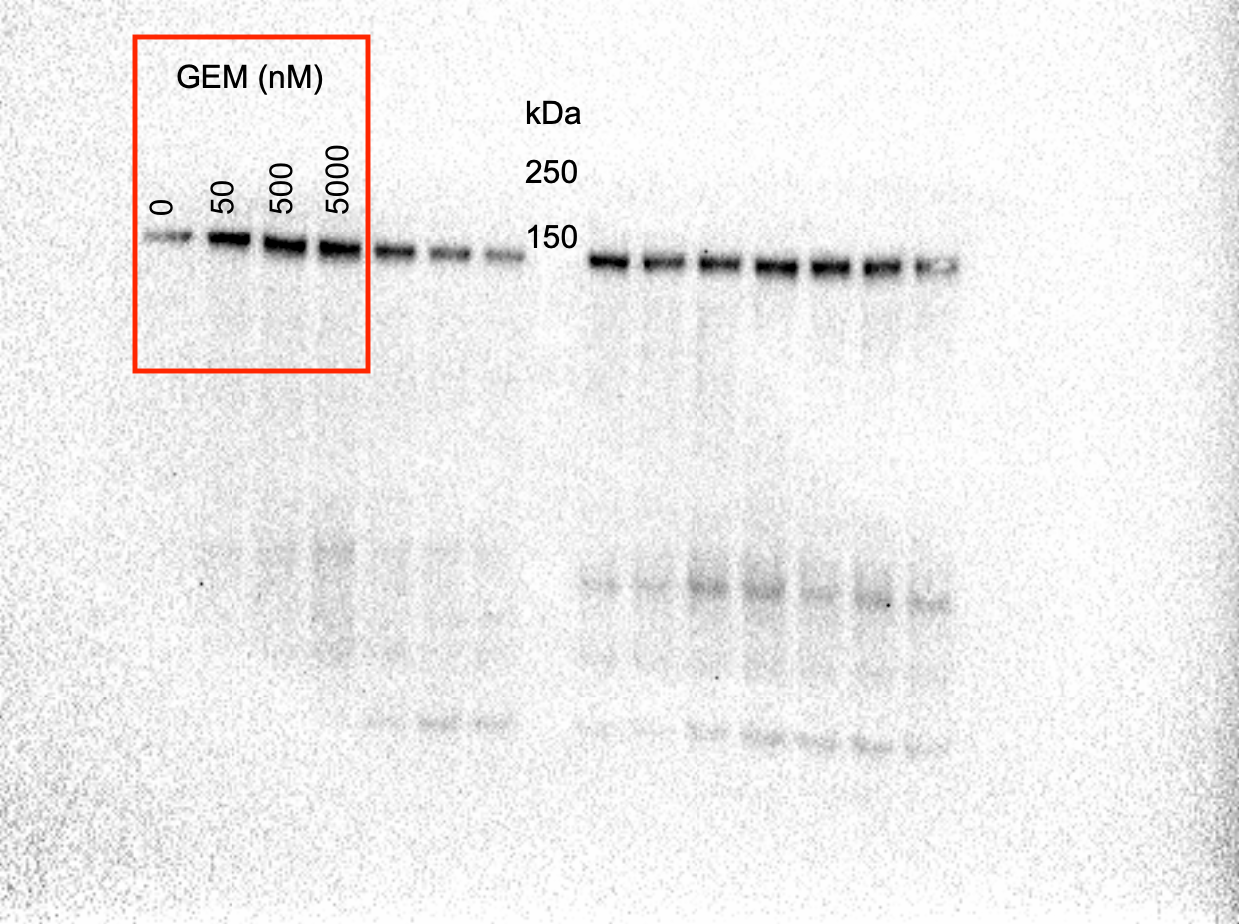

Supplement: Supplementary file 2 — Source data Fig. 1 [file 44318_2024_338_MOESM2_ESM.zip › SD figure 1/1E/MET WB GEM.tif]

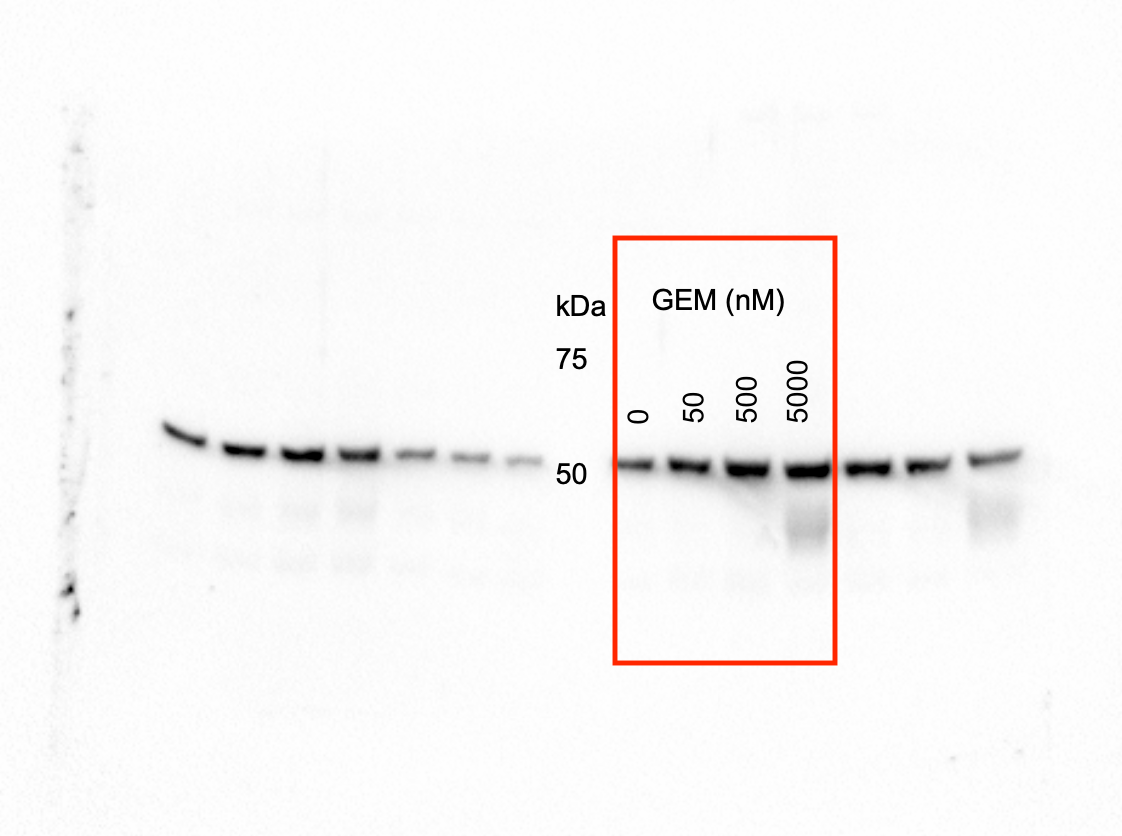

Supplement: Supplementary file 2 — Source data Fig. 1 [file 44318_2024_338_MOESM2_ESM.zip › SD figure 1/1E/ATF4 WB GEM.tif]

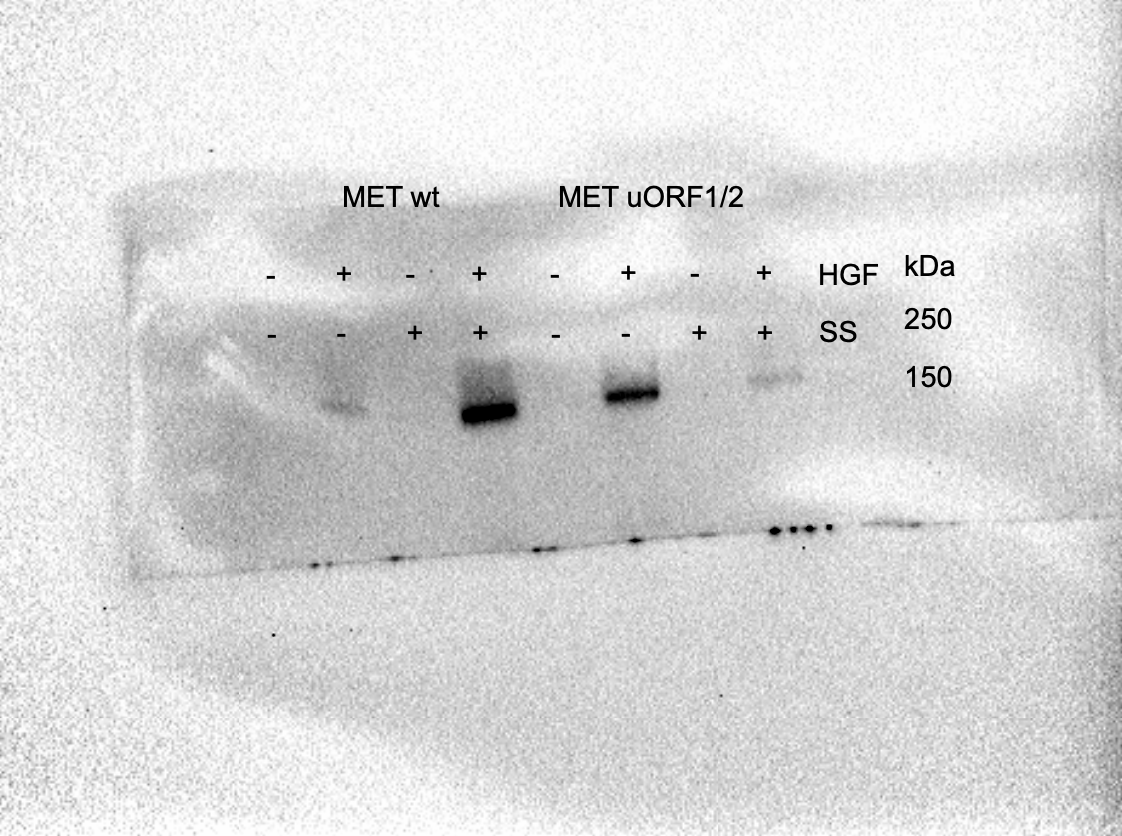

Supplement: Supplementary file 3 — Source data Fig. 2 [file 44318_2024_338_MOESM3_ESM.zip › SD figure 2/2A/MET-p WB uORF.tif]

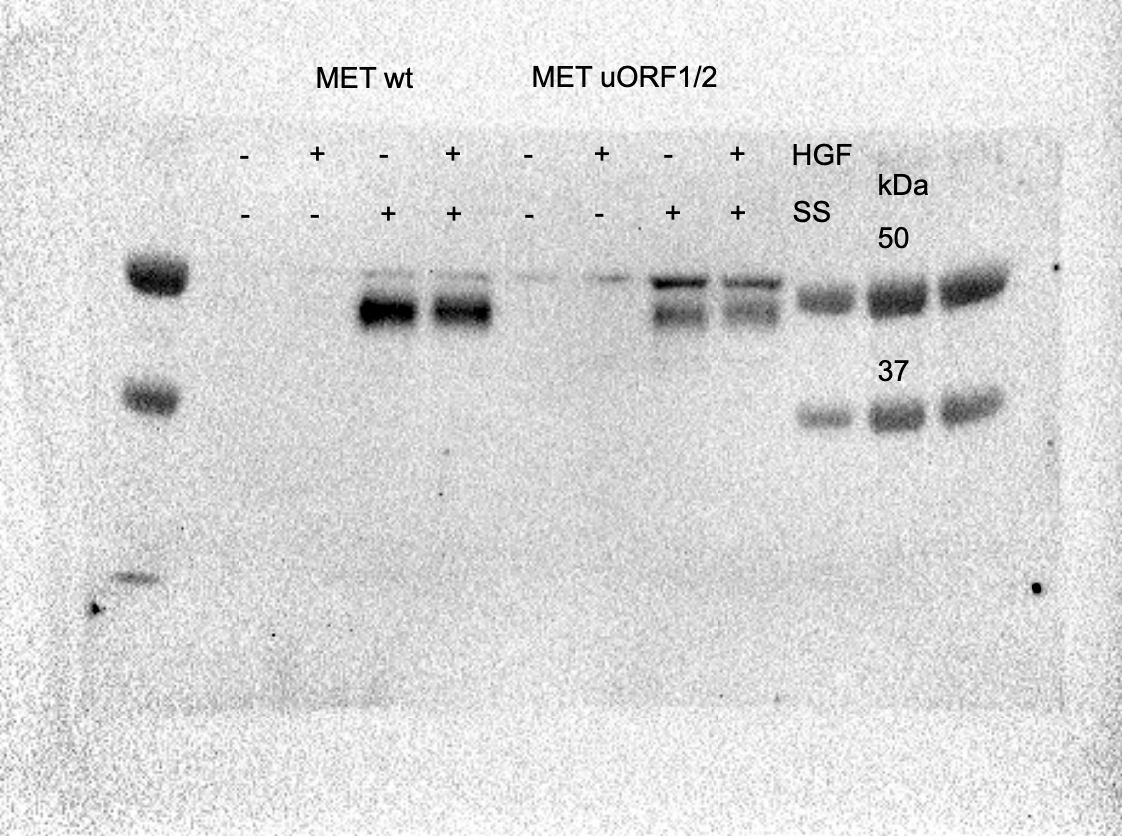

Supplement: Supplementary file 3 — Source data Fig. 2 [file 44318_2024_338_MOESM3_ESM.zip › SD figure 2/2A/ATF4 WB uORF.tif]

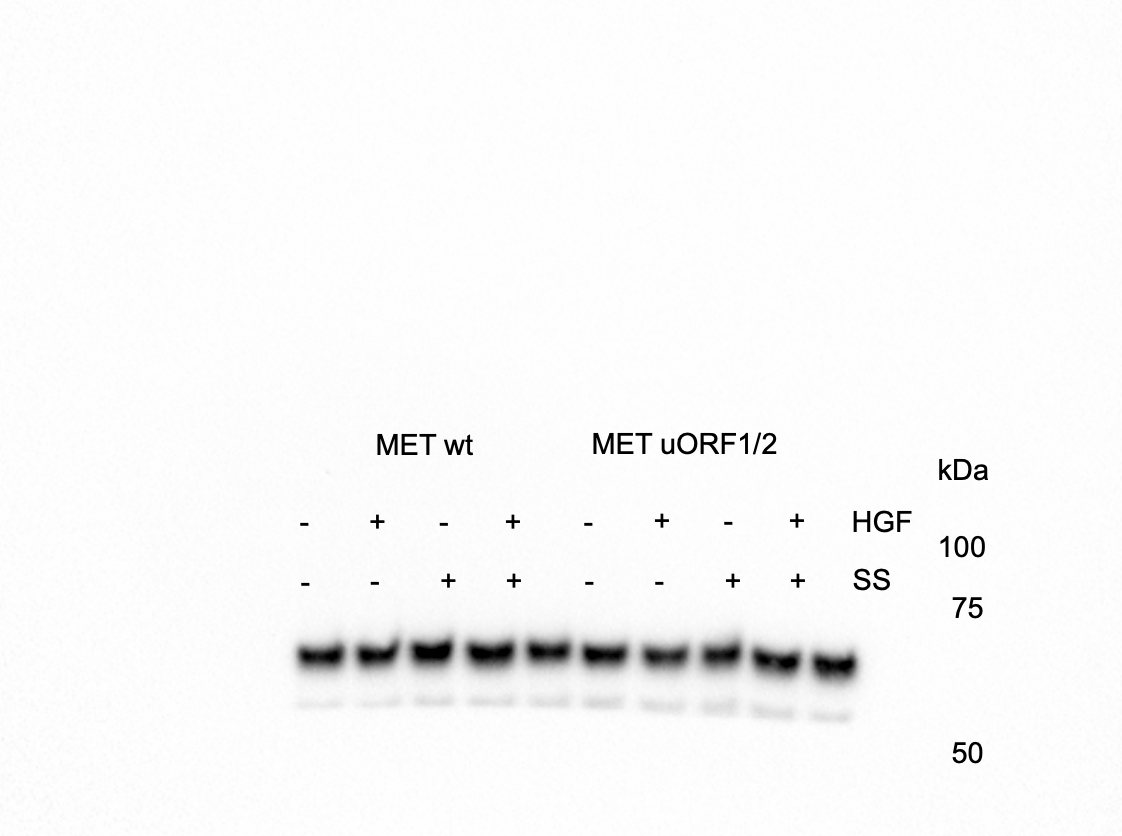

Supplement: Supplementary file 3 — Source data Fig. 2 [file 44318_2024_338_MOESM3_ESM.zip › SD figure 2/2A/AKT WB uORF.tif]

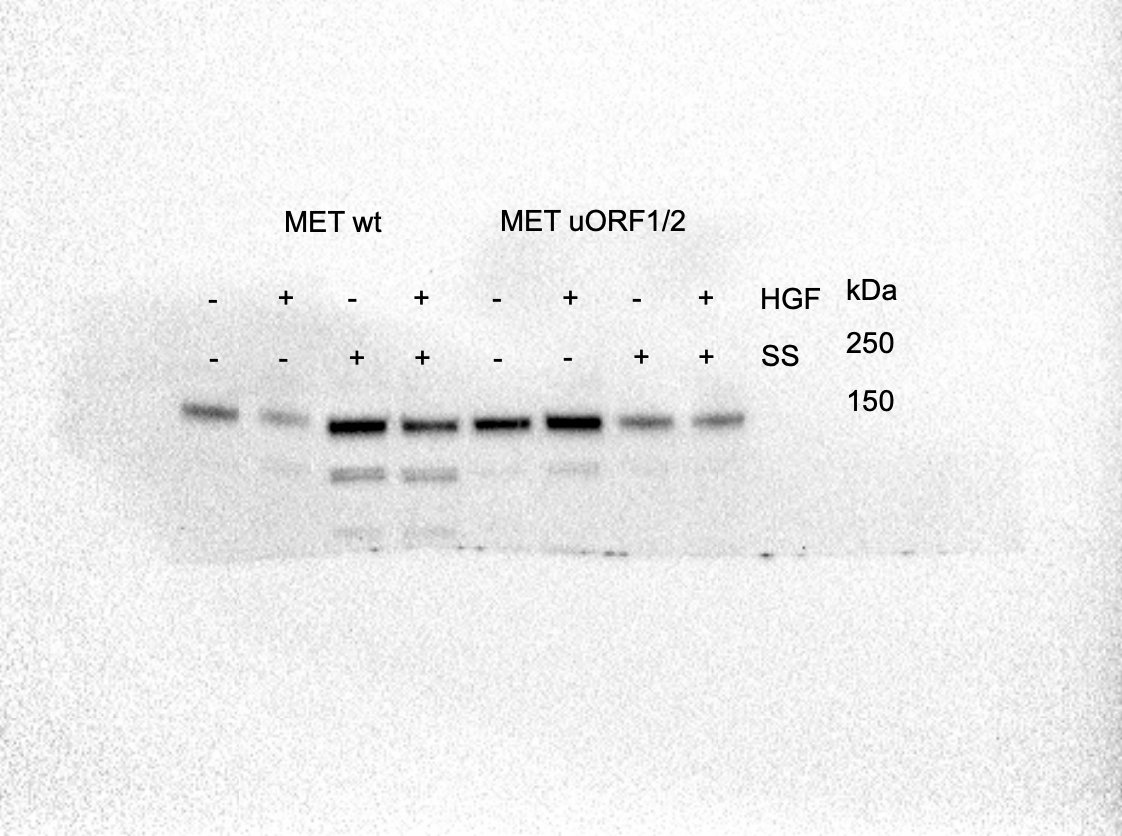

Supplement: Supplementary file 3 — Source data Fig. 2 [file 44318_2024_338_MOESM3_ESM.zip › SD figure 2/2A/MET WB uORF.tif]

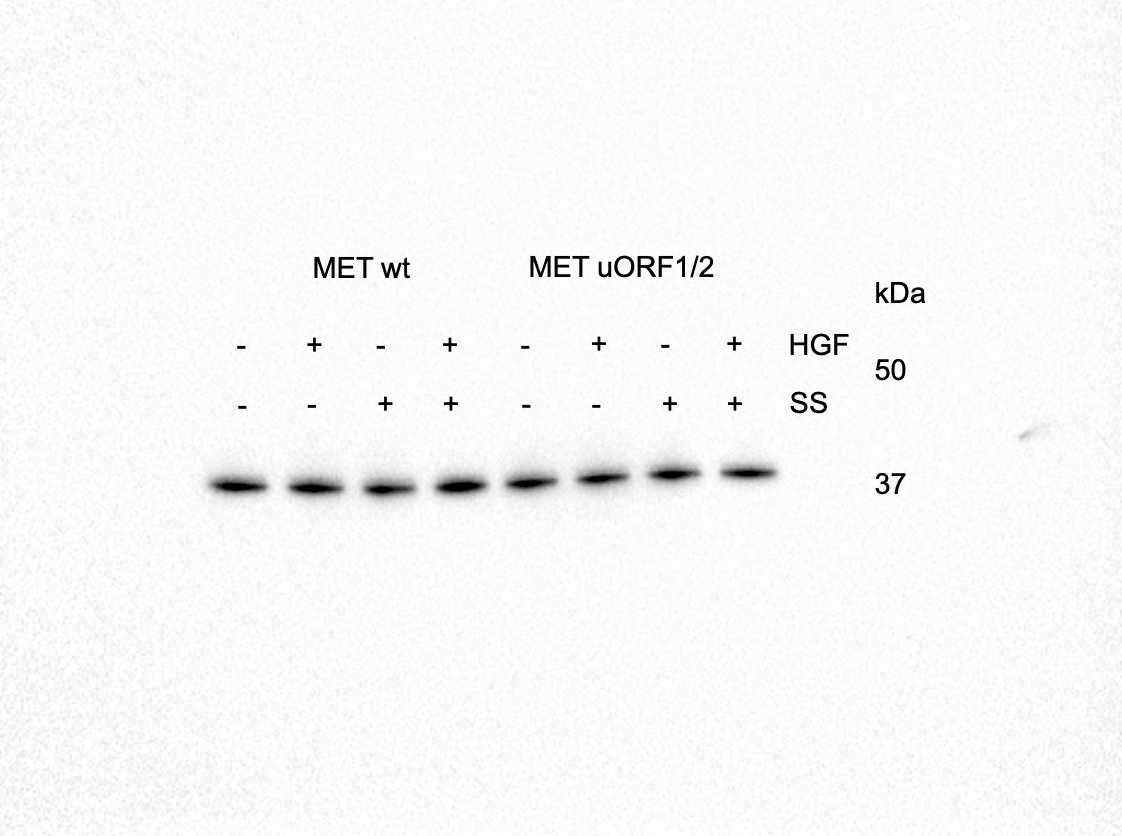

Supplement: Supplementary file 3 — Source data Fig. 2 [file 44318_2024_338_MOESM3_ESM.zip › SD figure 2/2A/TBP WB uORF.tif]

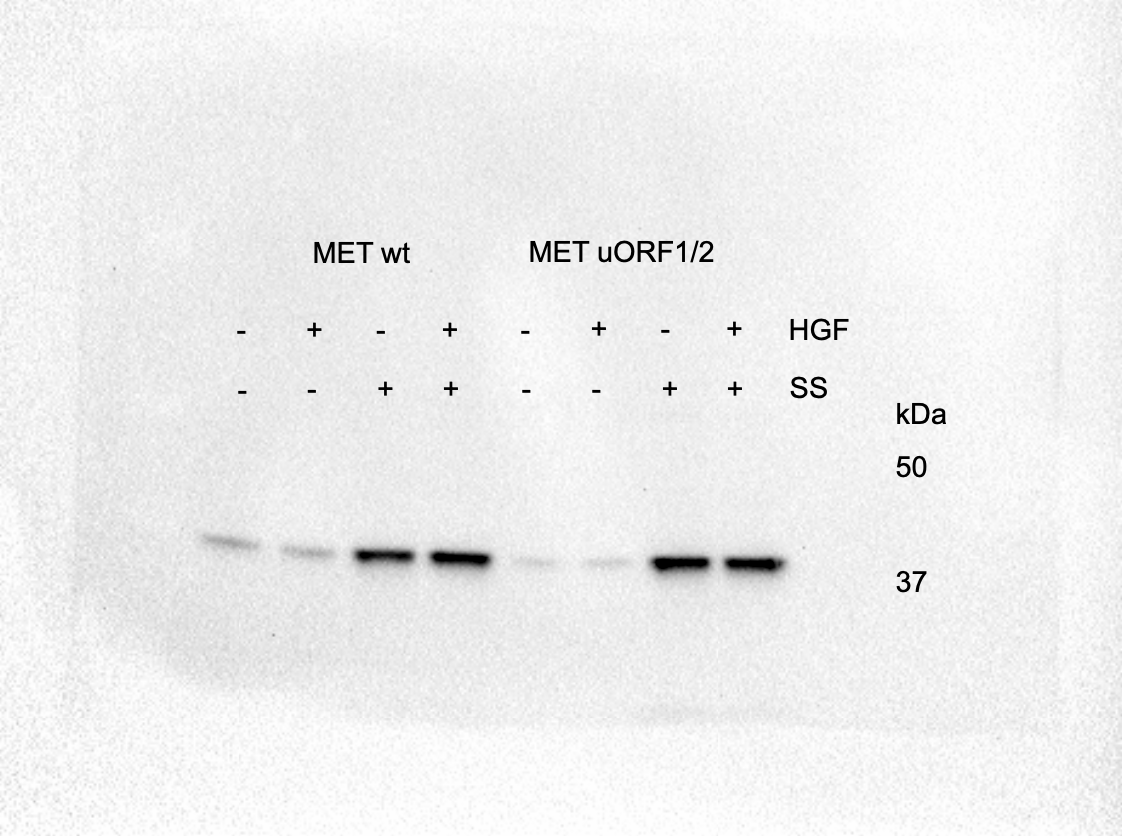

Supplement: Supplementary file 3 — Source data Fig. 2 [file 44318_2024_338_MOESM3_ESM.zip › SD figure 2/2A/eIF2a-P WB uORF.tif]

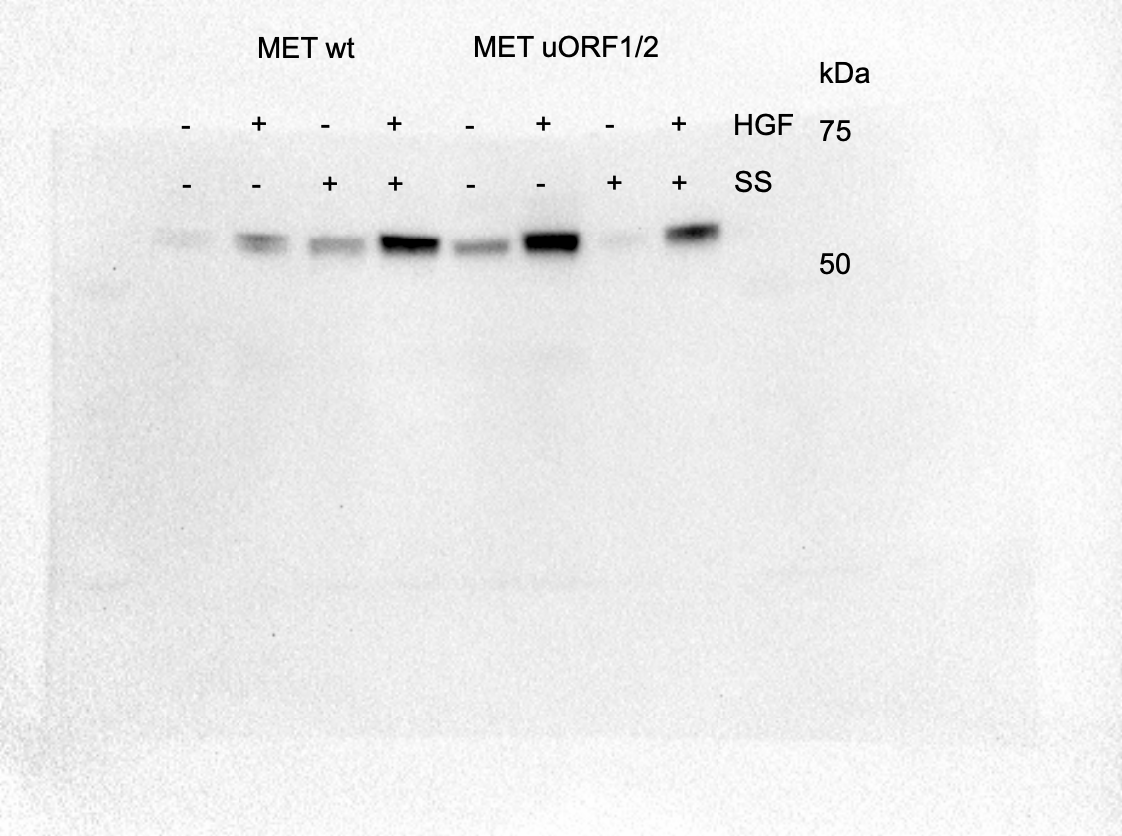

Supplement: Supplementary file 3 — Source data Fig. 2 [file 44318_2024_338_MOESM3_ESM.zip › SD figure 2/2A/AKT-p WB uORF.tif]

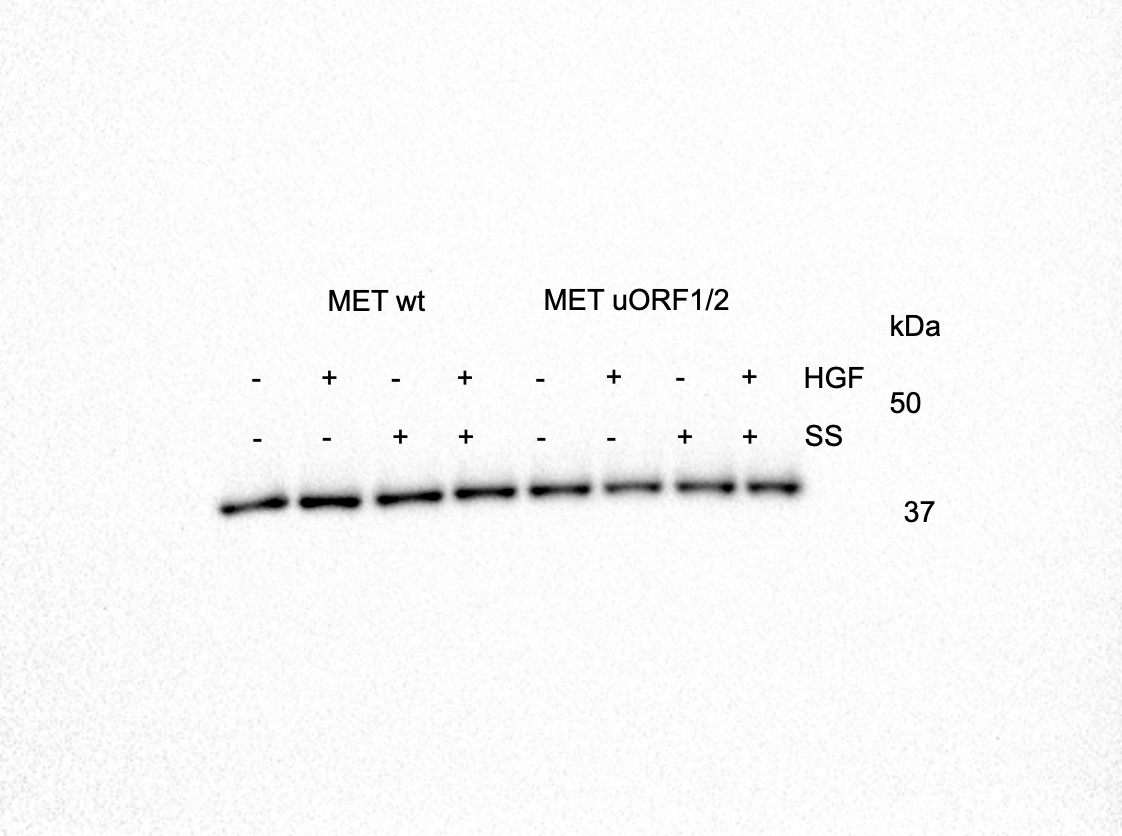

Supplement: Supplementary file 3 — Source data Fig. 2 [file 44318_2024_338_MOESM3_ESM.zip › SD figure 2/2A/eIF2a WB uORF.tif]

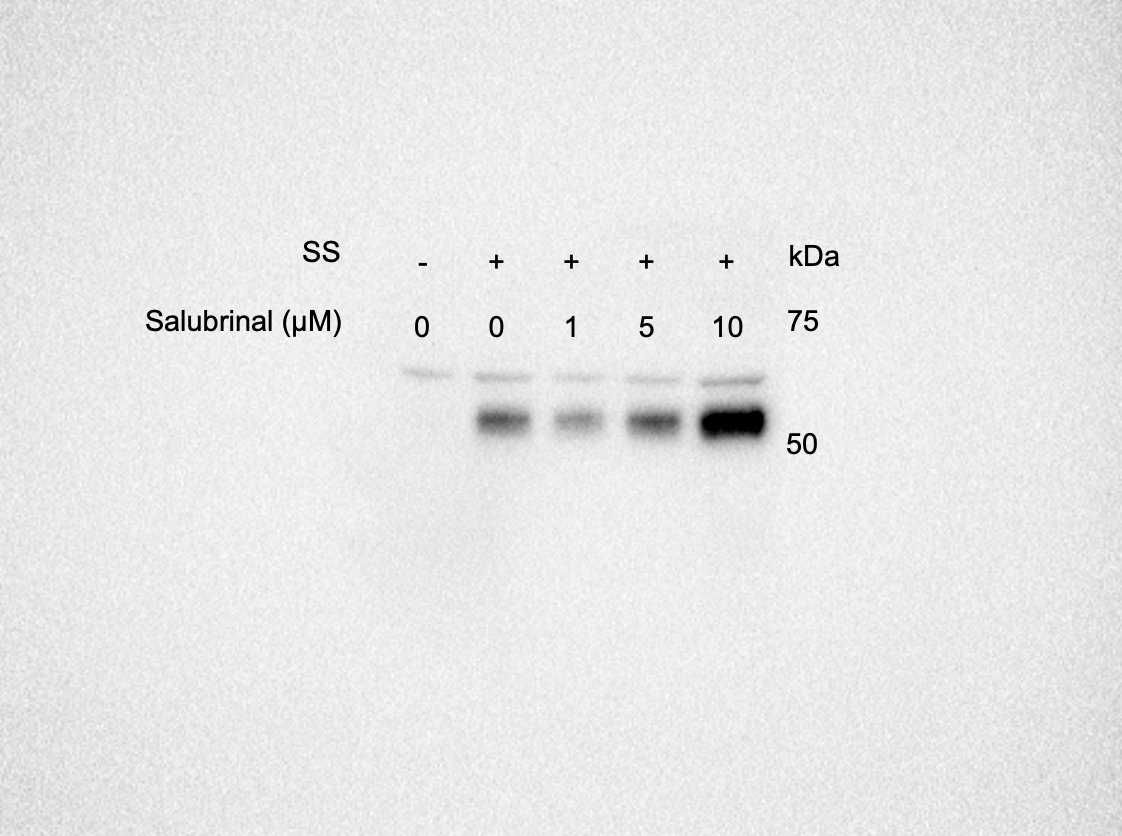

Supplement: Supplementary file 4 — Source data Fig. 3 [file 44318_2024_338_MOESM4_ESM.zip › SD figure 3/3B/ATF4 WB.tif]

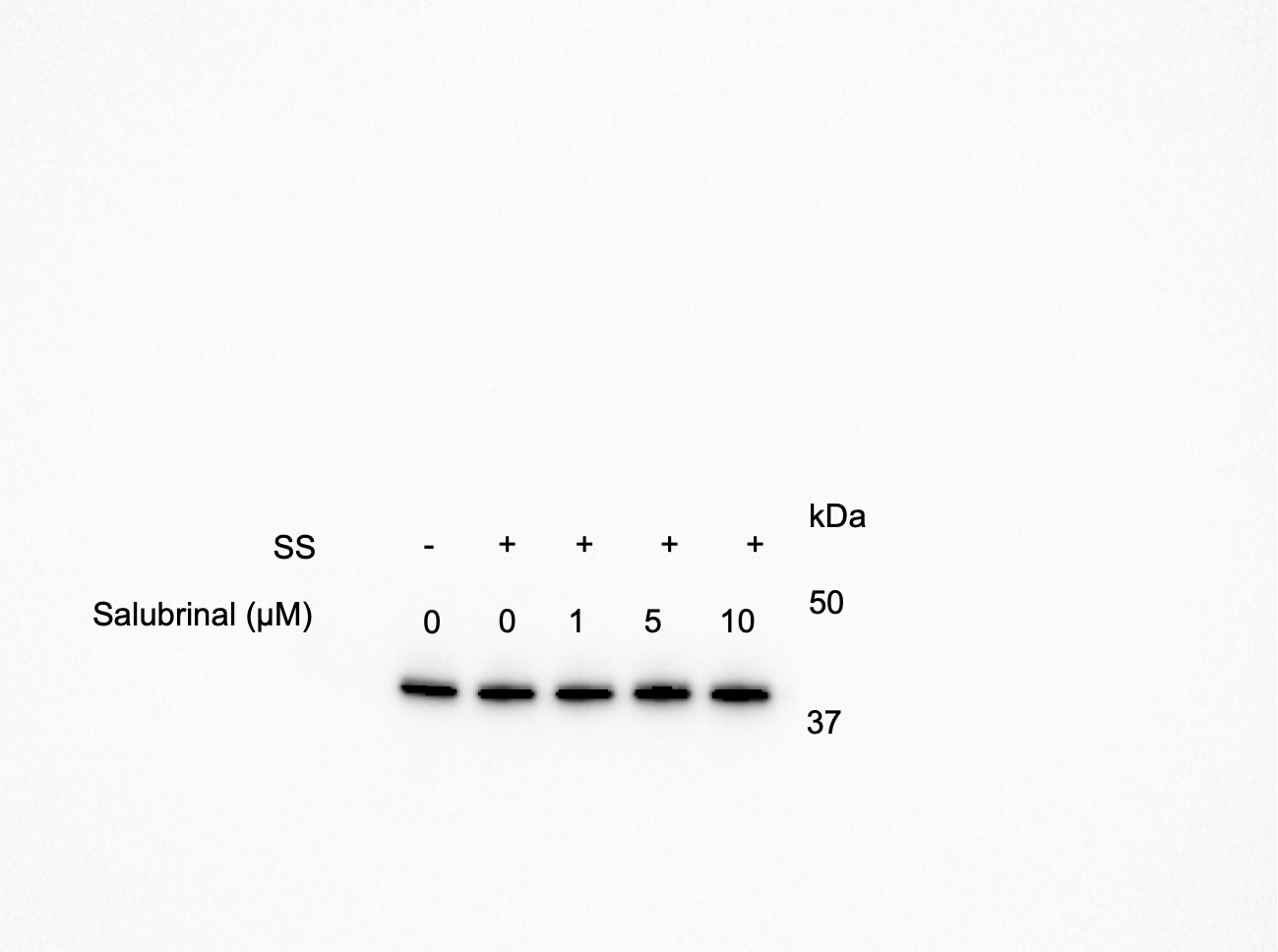

Supplement: Supplementary file 4 — Source data Fig. 3 [file 44318_2024_338_MOESM4_ESM.zip › SD figure 3/3B/TBP WB.tif]

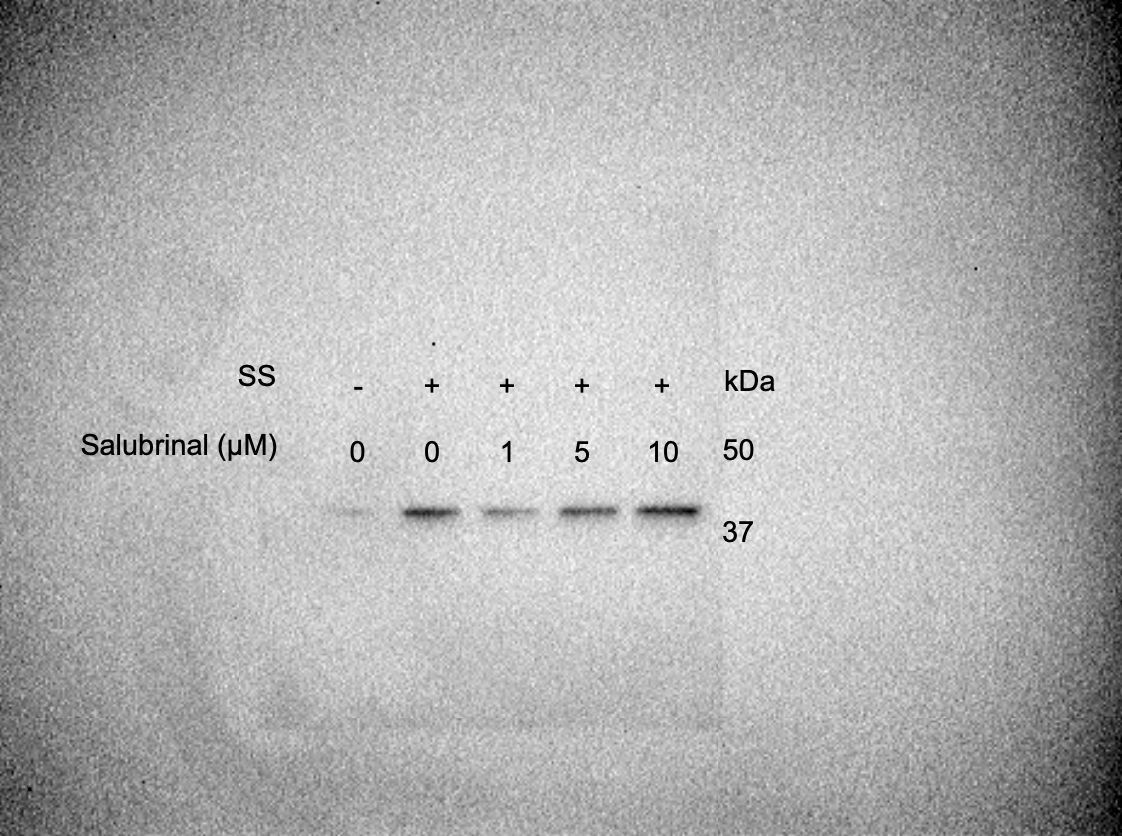

Supplement: Supplementary file 4 — Source data Fig. 3 [file 44318_2024_338_MOESM4_ESM.zip › SD figure 3/3B/eIF2alpha P WB.tif]

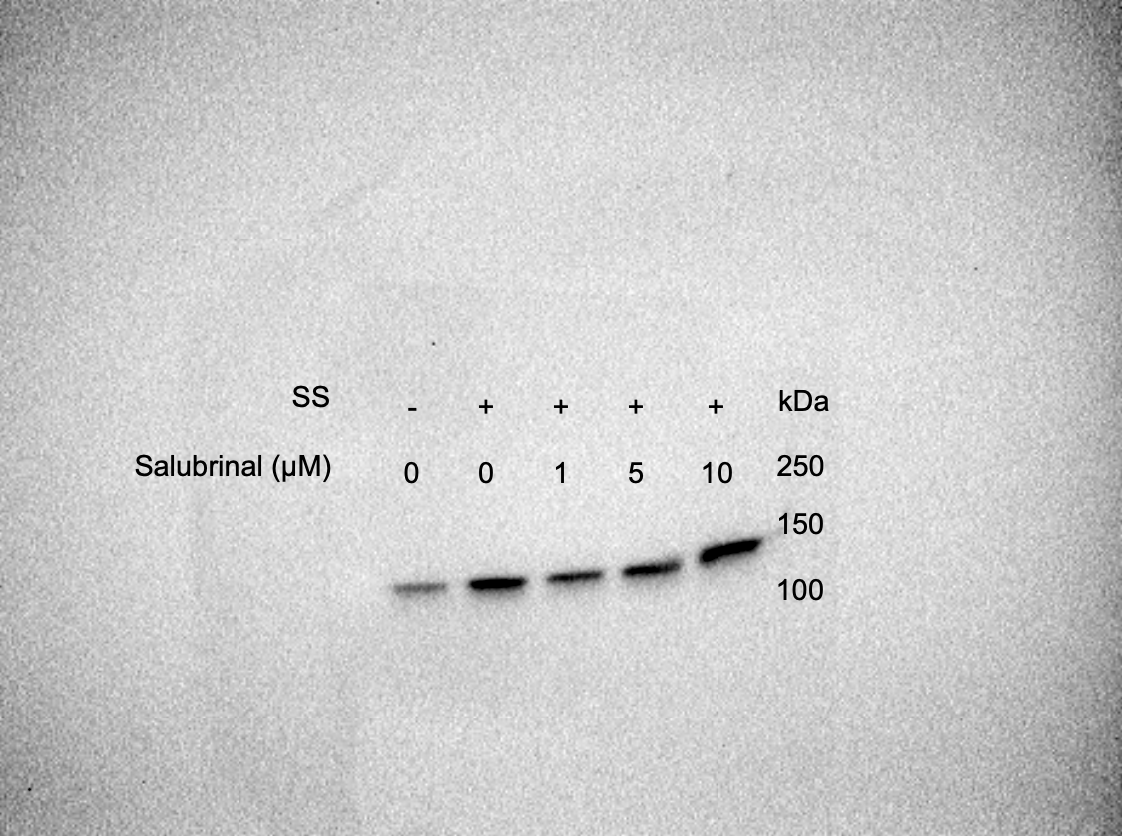

Supplement: Supplementary file 4 — Source data Fig. 3 [file 44318_2024_338_MOESM4_ESM.zip › SD figure 3/3B/MET WB.tif]

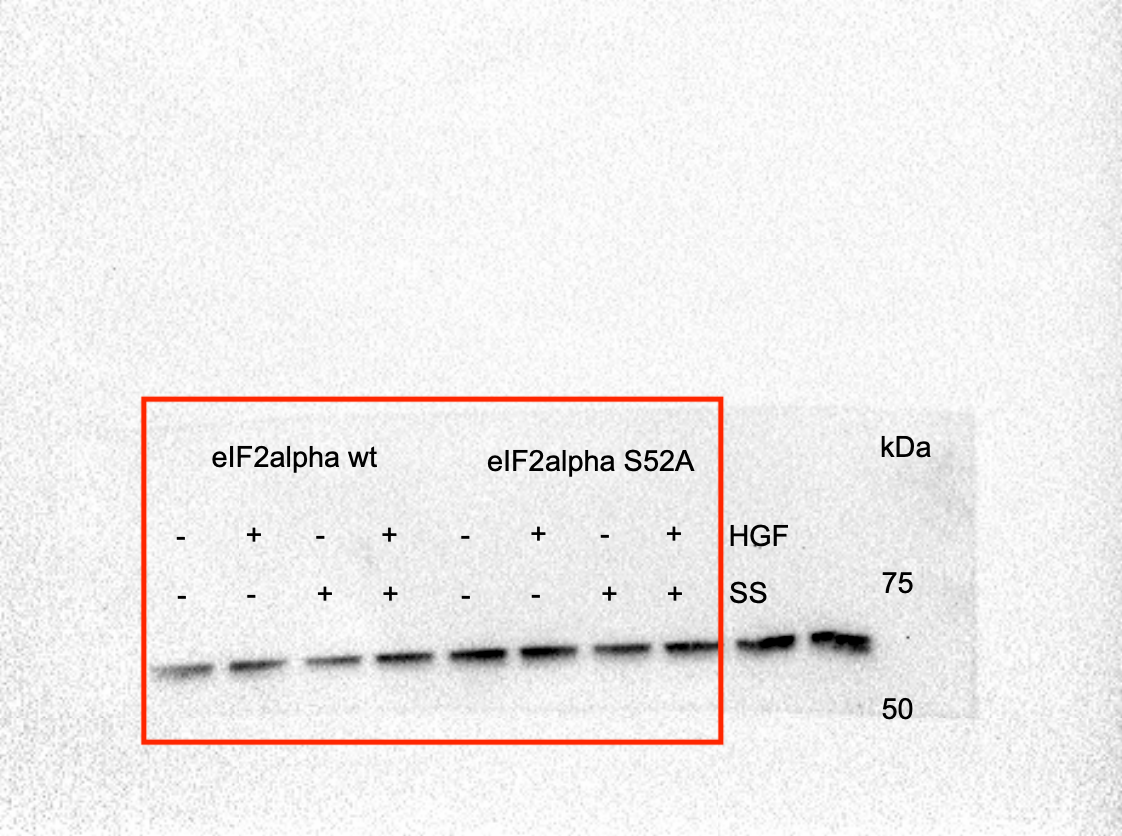

Supplement: Supplementary file 4 — Source data Fig. 3 [file 44318_2024_338_MOESM4_ESM.zip › SD figure 3/3D/AKT WB.tif]

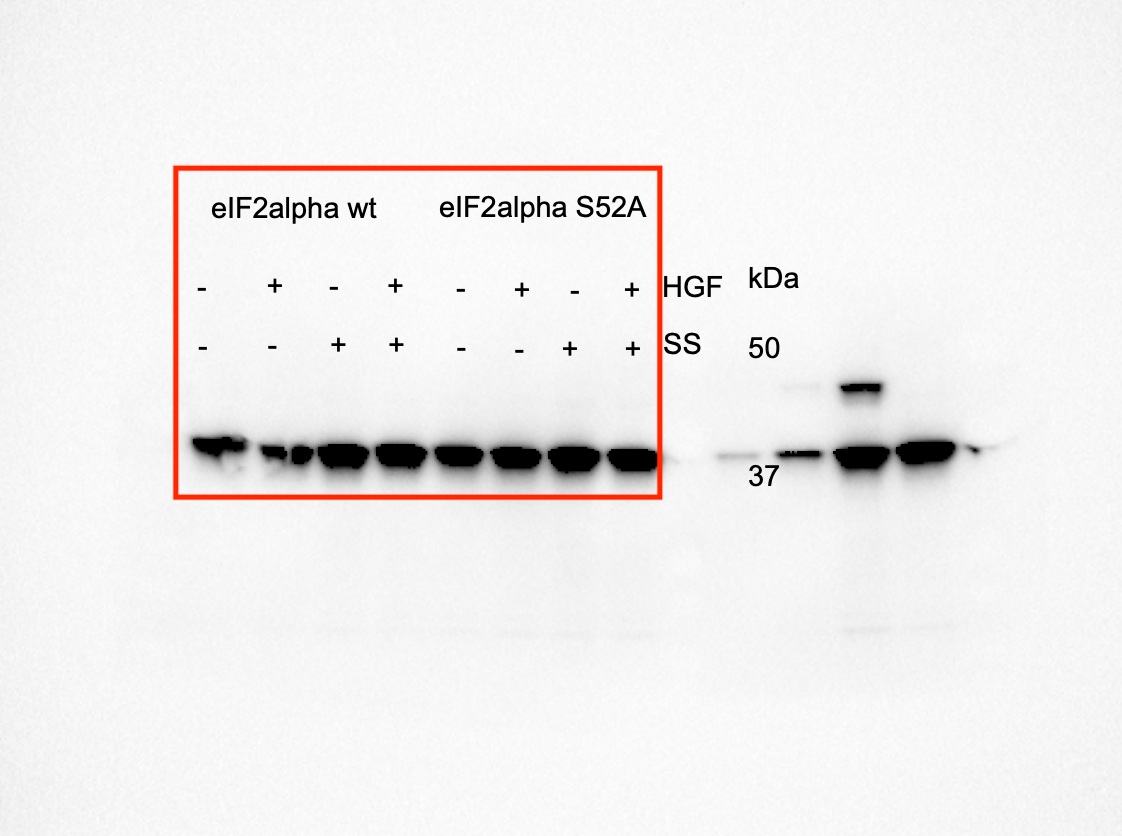

Supplement: Supplementary file 4 — Source data Fig. 3 [file 44318_2024_338_MOESM4_ESM.zip › SD figure 3/3D/eIF2a WB.tif]

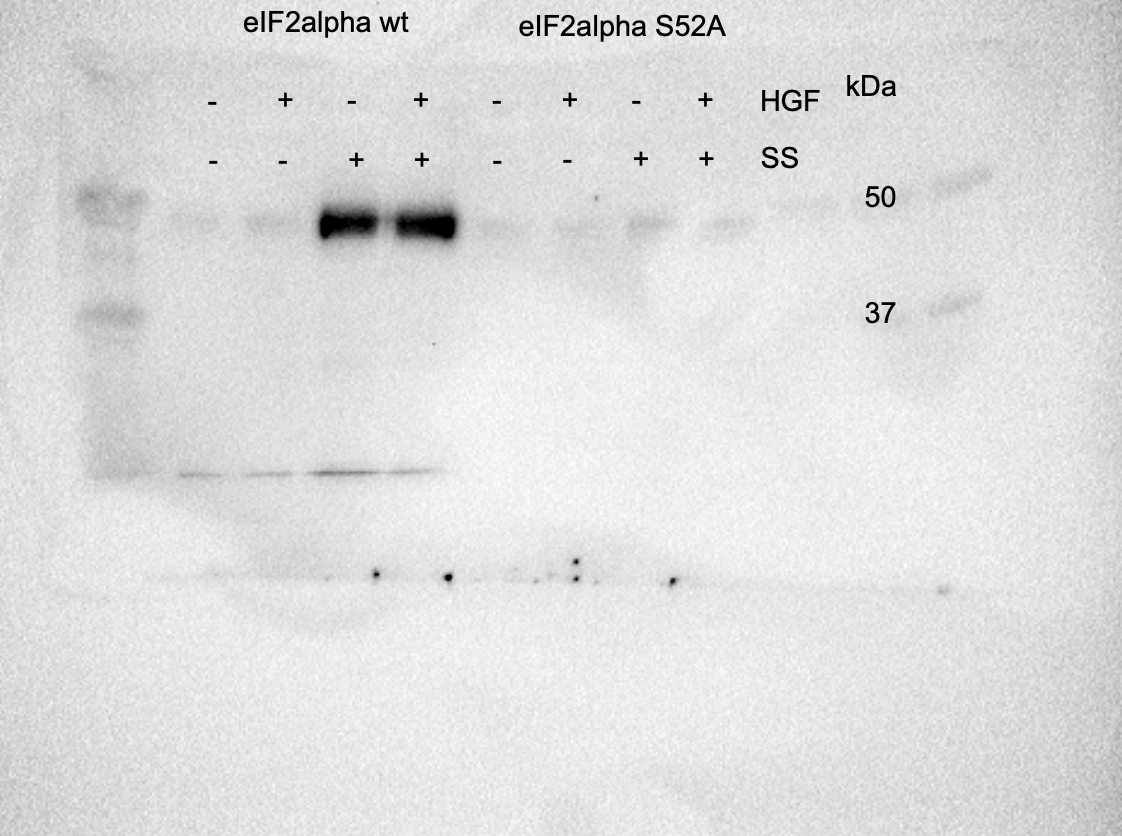

Supplement: Supplementary file 4 — Source data Fig. 3 [file 44318_2024_338_MOESM4_ESM.zip › SD figure 3/3D/ATF4 WB.tif]

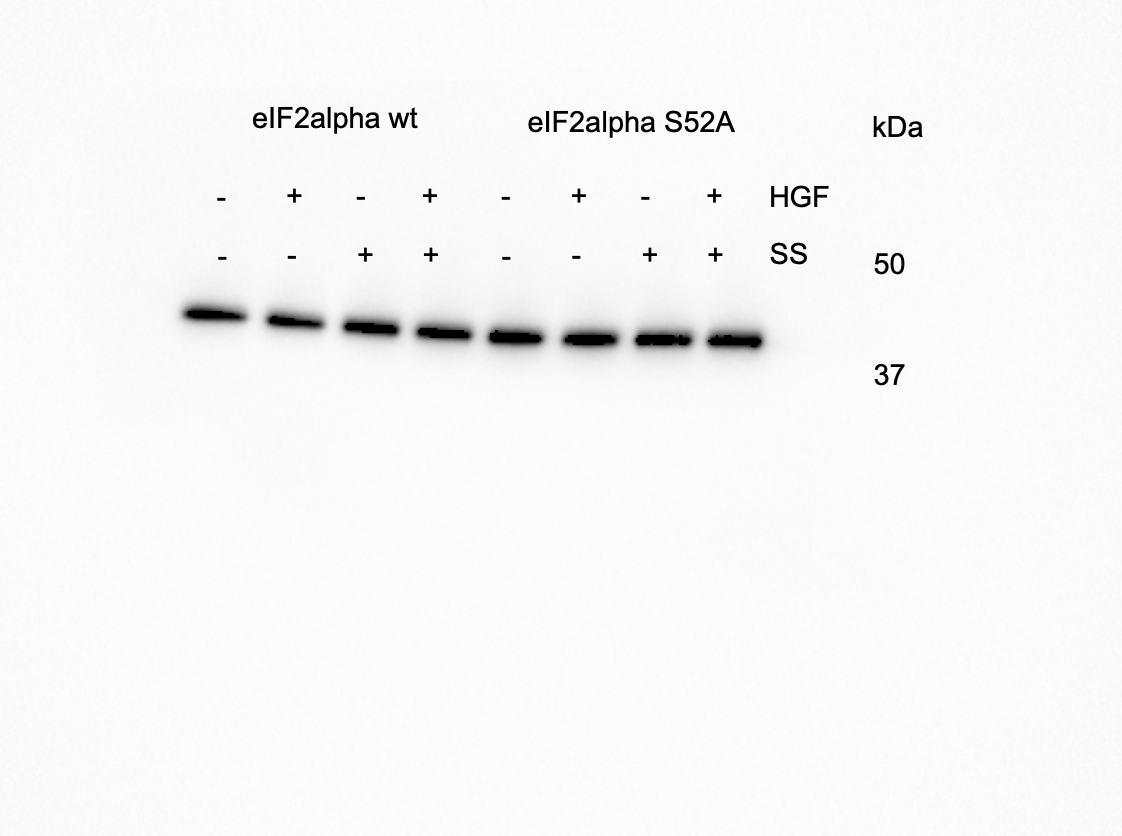

Supplement: Supplementary file 4 — Source data Fig. 3 [file 44318_2024_338_MOESM4_ESM.zip › SD figure 3/3D/TBP WB.tif]

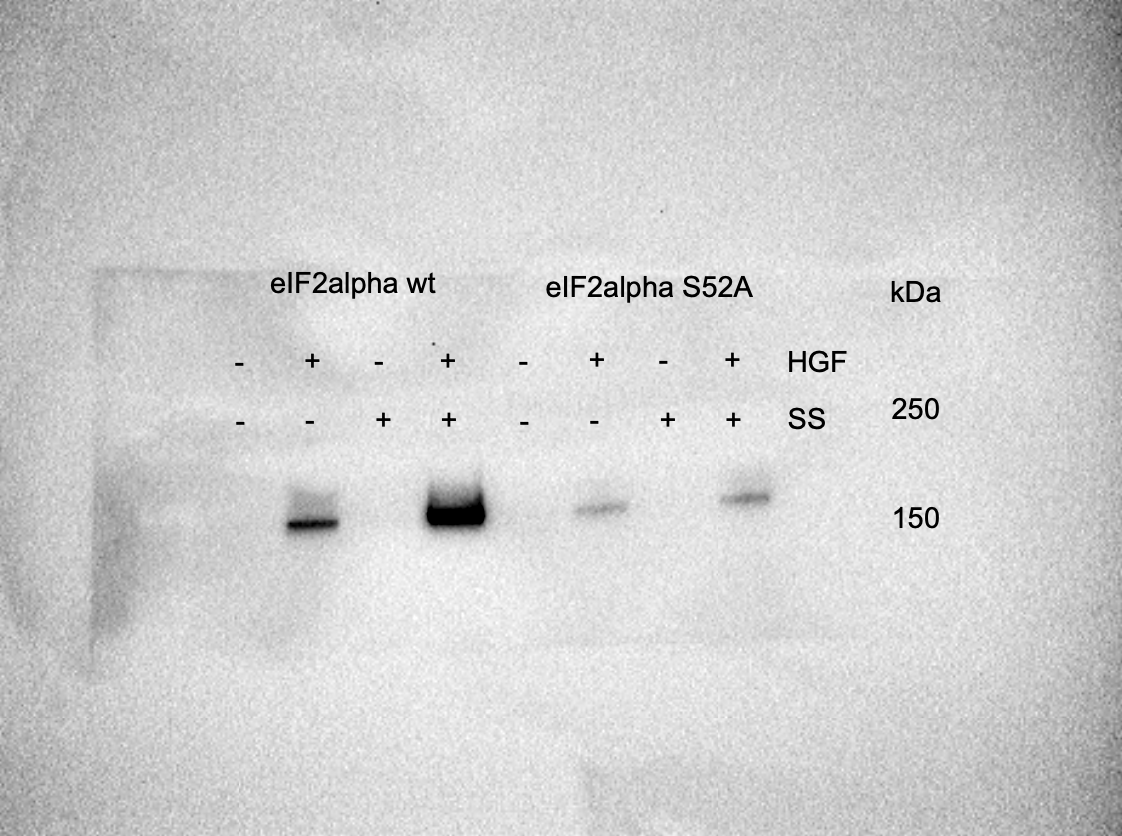

Supplement: Supplementary file 4 — Source data Fig. 3 [file 44318_2024_338_MOESM4_ESM.zip › SD figure 3/3D/MET-P WB.tif]

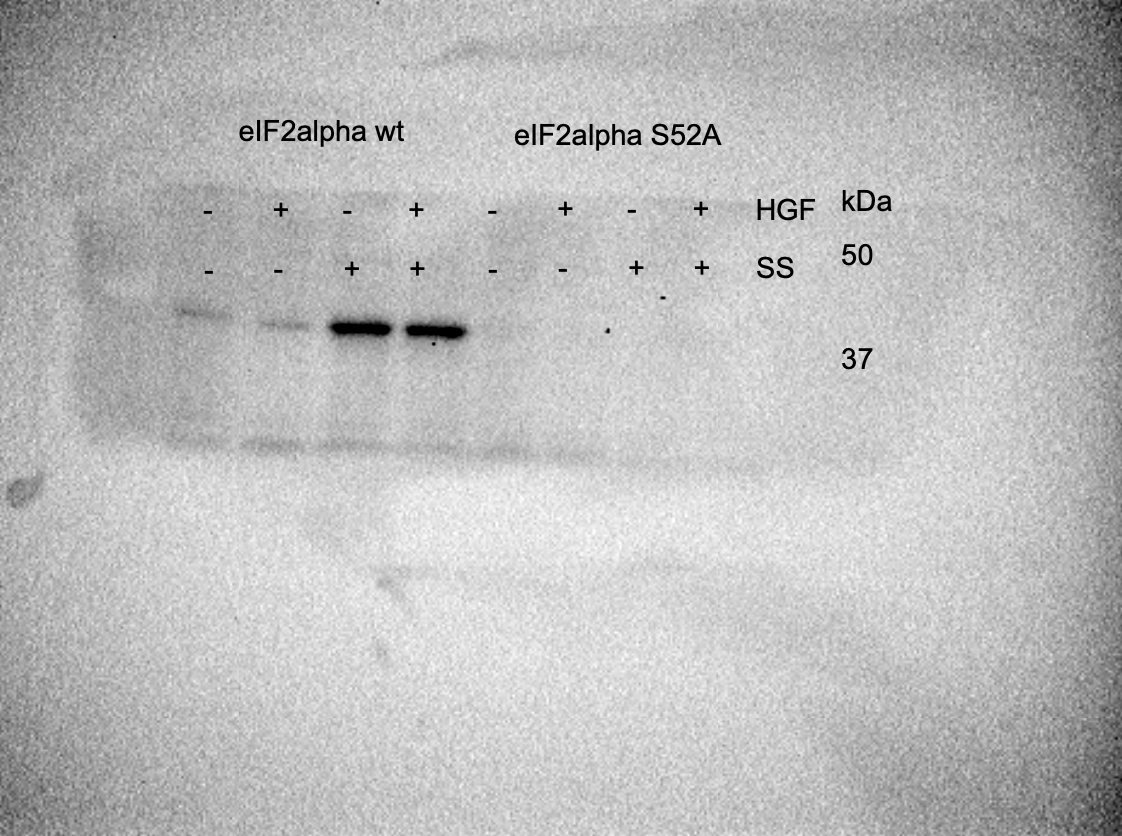

Supplement: Supplementary file 4 — Source data Fig. 3 [file 44318_2024_338_MOESM4_ESM.zip › SD figure 3/3D/eIF2a-P WB.tif]

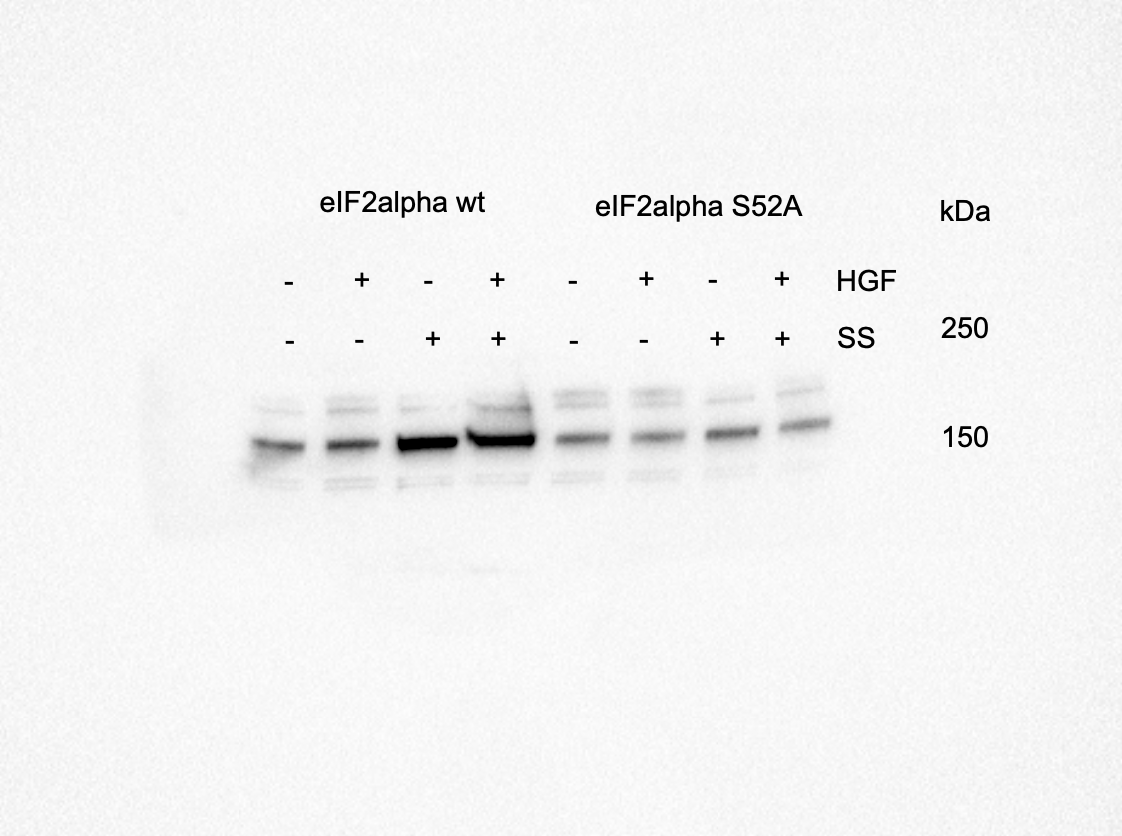

Supplement: Supplementary file 4 — Source data Fig. 3 [file 44318_2024_338_MOESM4_ESM.zip › SD figure 3/3D/MET WB.tif]

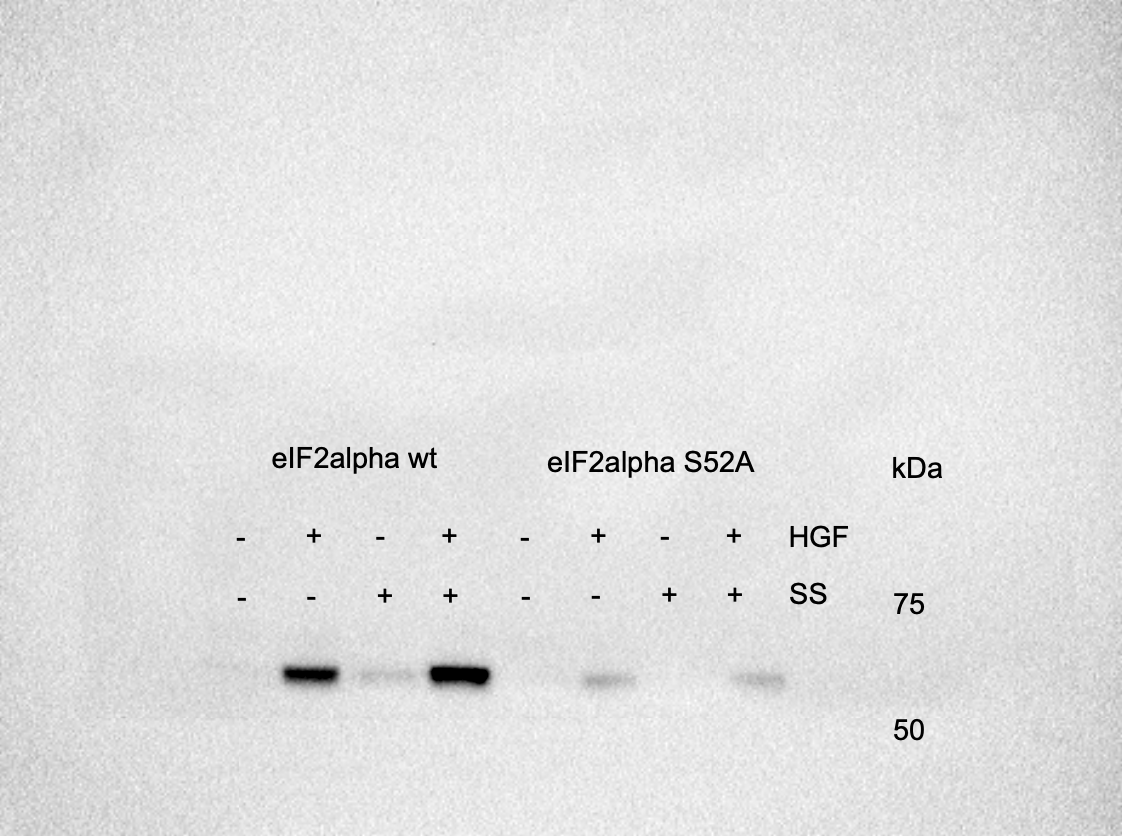

Supplement: Supplementary file 4 — Source data Fig. 3 [file 44318_2024_338_MOESM4_ESM.zip › SD figure 3/3D/AKT-P WB.tif]

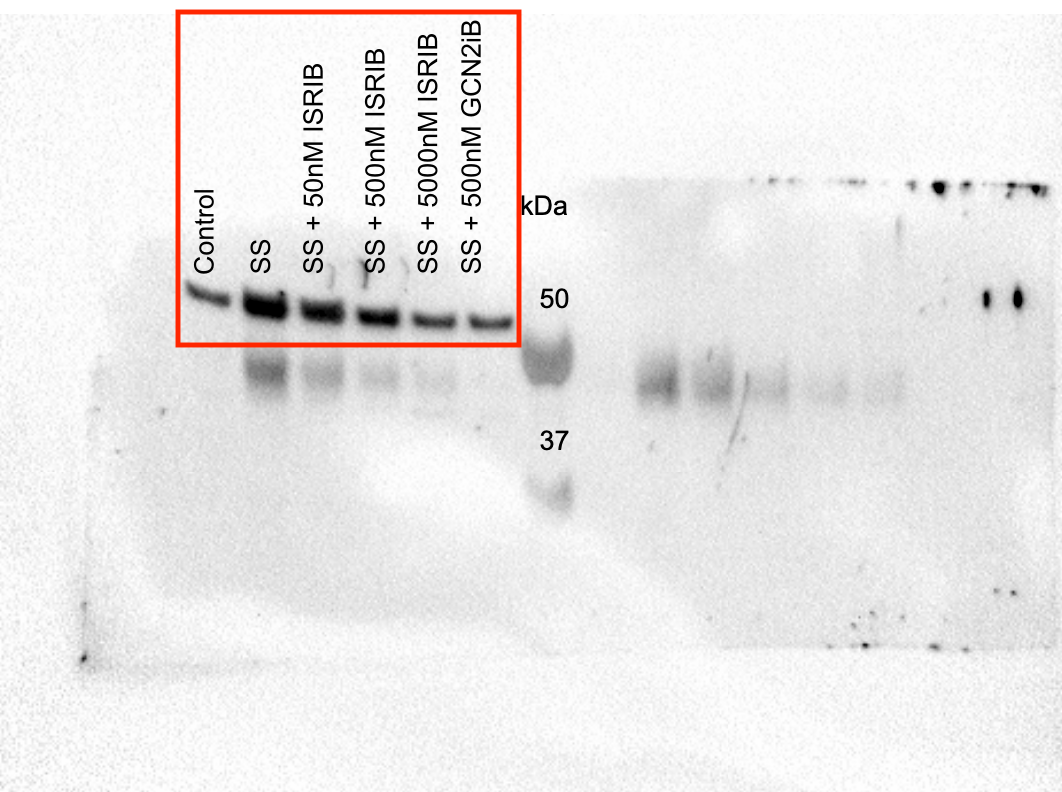

Supplement: Supplementary file 5 — Source data Fig. 4 [file 44318_2024_338_MOESM5_ESM.zip › SD figure 4/4E/ATF4 WB.tif]

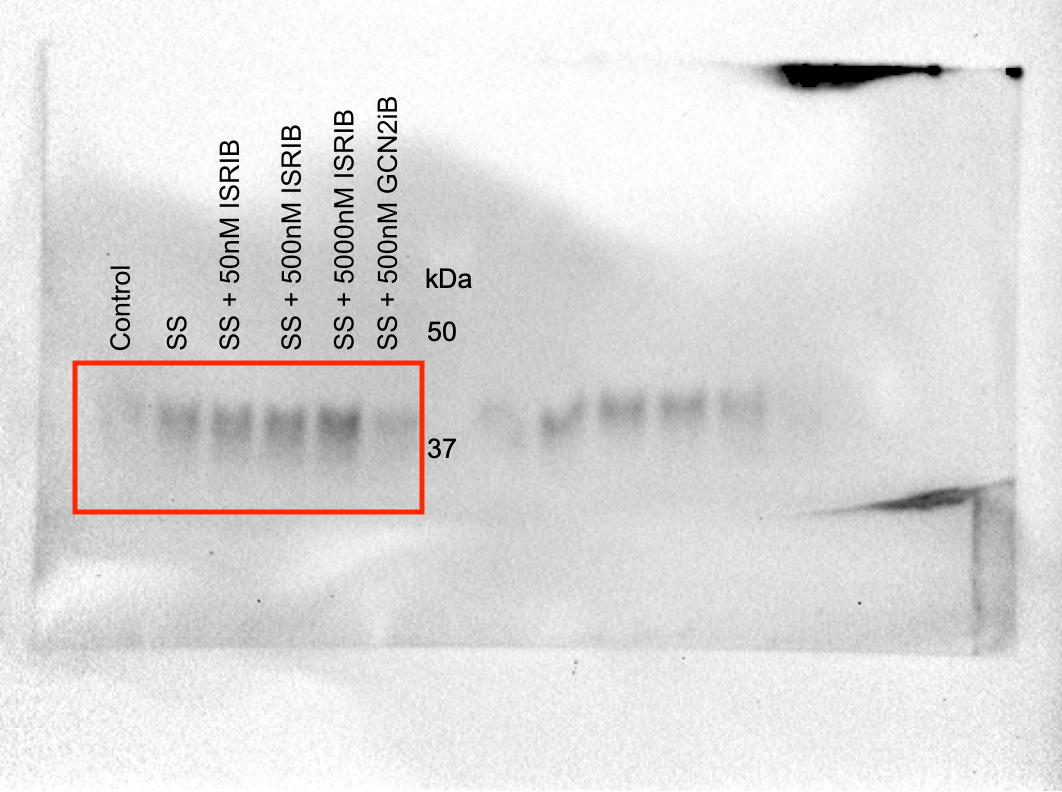

Supplement: Supplementary file 5 — Source data Fig. 4 [file 44318_2024_338_MOESM5_ESM.zip › SD figure 4/4E/eIF2alpha-P WB.tif]

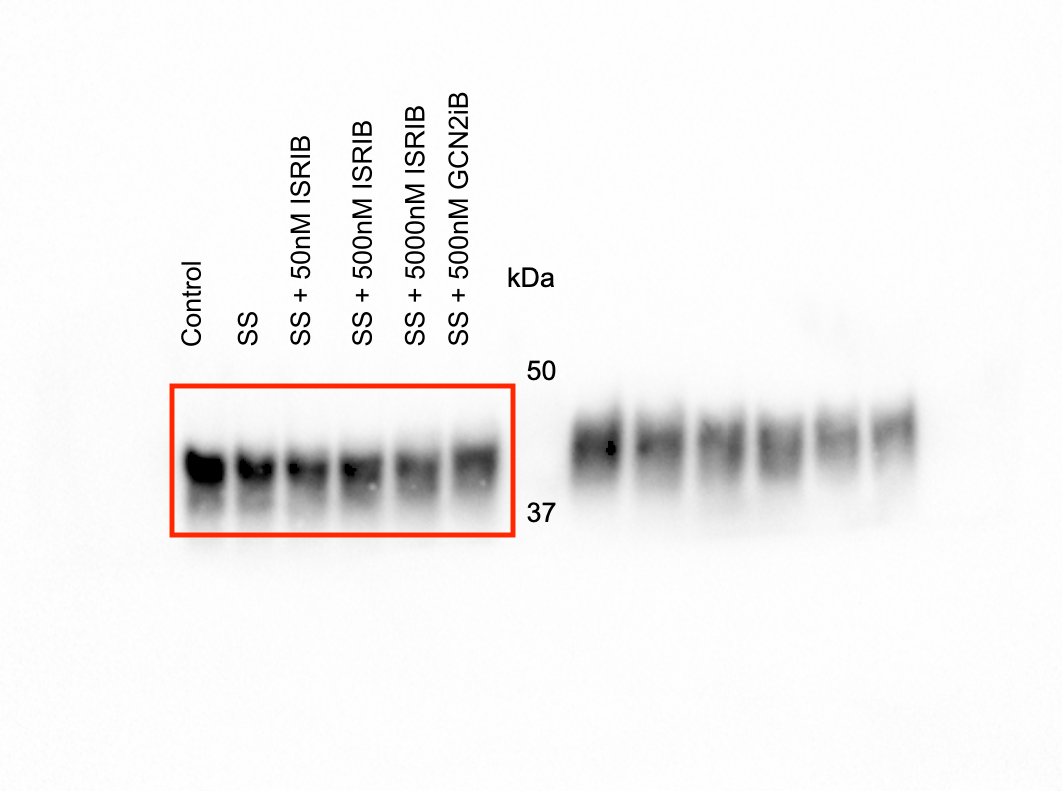

Supplement: Supplementary file 5 — Source data Fig. 4 [file 44318_2024_338_MOESM5_ESM.zip › SD figure 4/4E/TBP WB.tif]

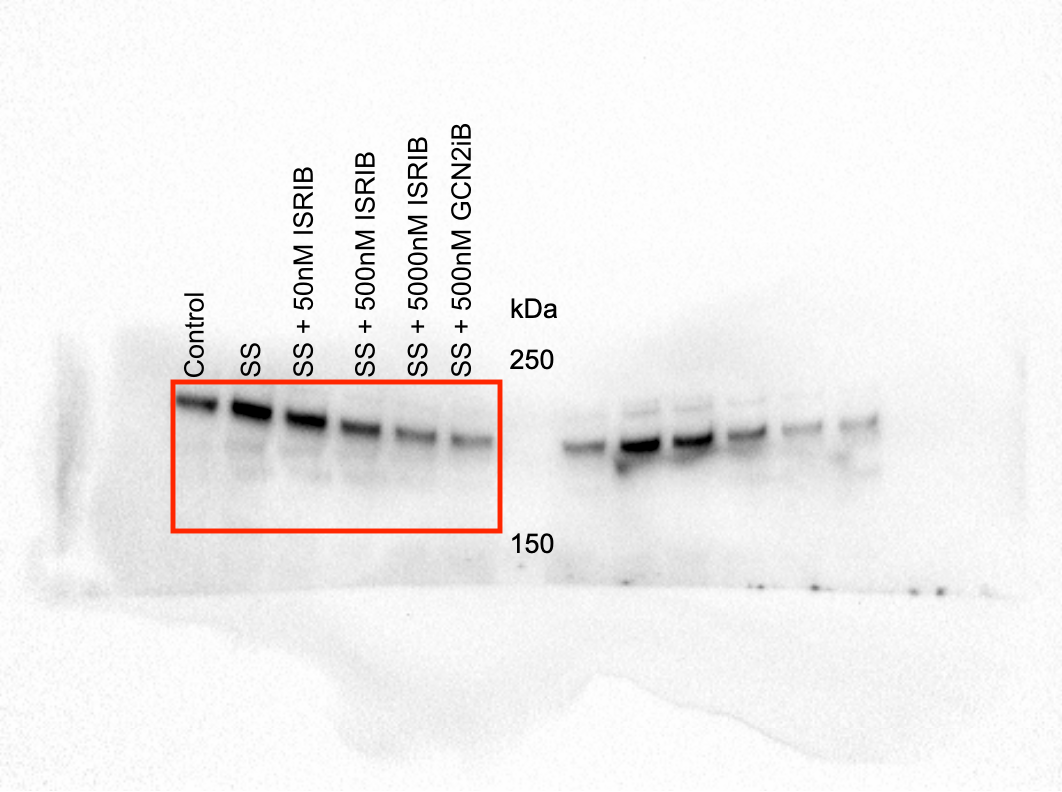

Supplement: Supplementary file 5 — Source data Fig. 4 [file 44318_2024_338_MOESM5_ESM.zip › SD figure 4/4E/MET WB.tif]

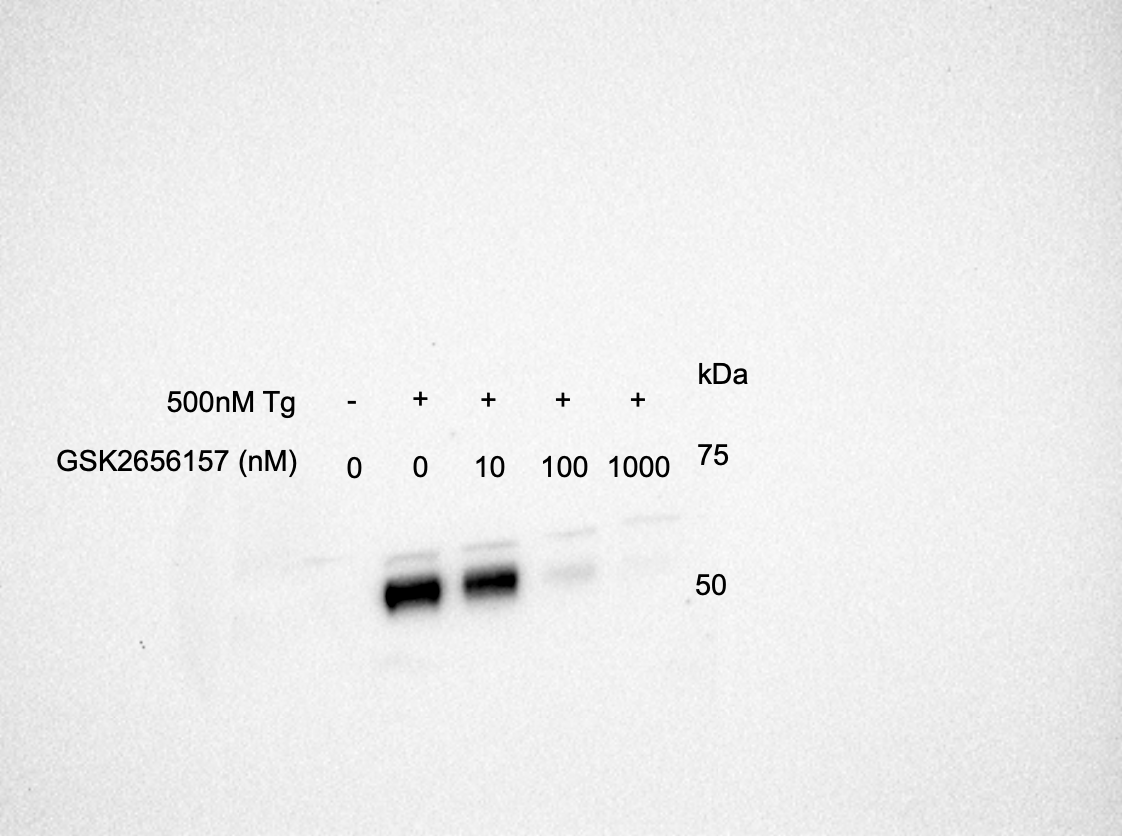

Supplement: Supplementary file 5 — Source data Fig. 4 [file 44318_2024_338_MOESM5_ESM.zip › SD figure 4/4C/ATF4 WB.tif]

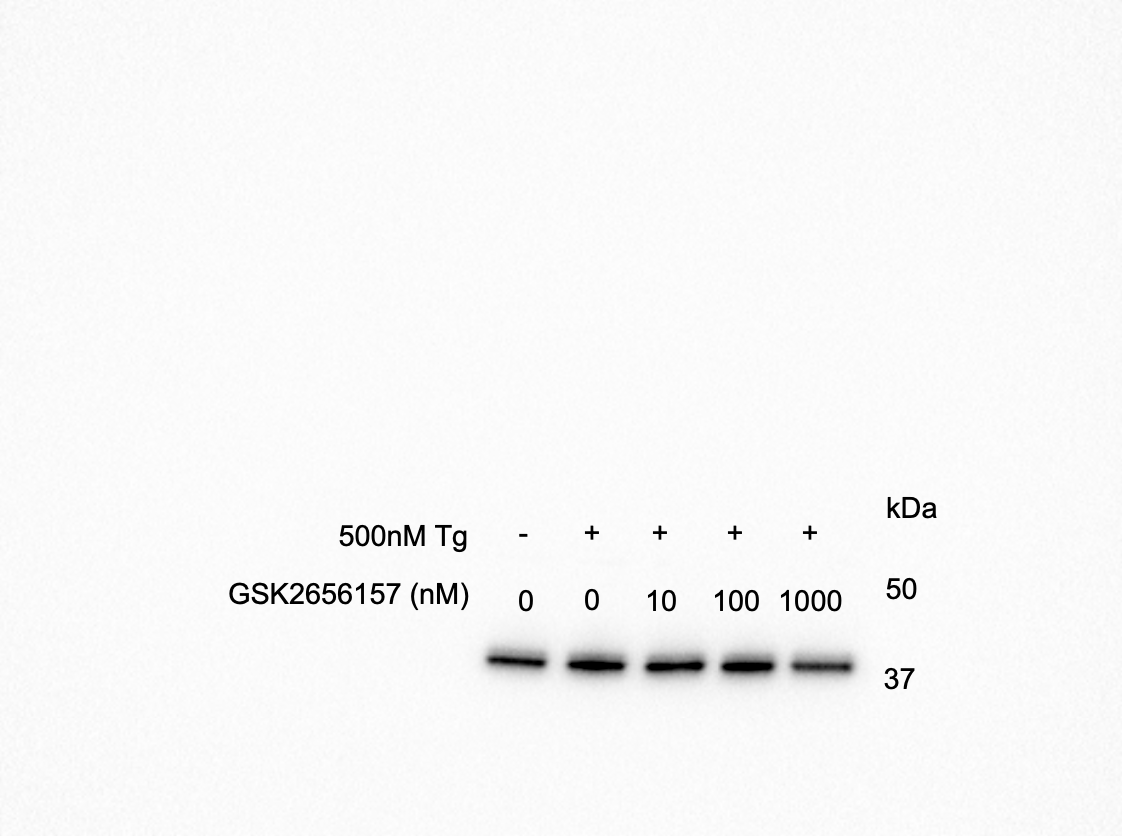

Supplement: Supplementary file 5 — Source data Fig. 4 [file 44318_2024_338_MOESM5_ESM.zip › SD figure 4/4C/TBP WB.tif]

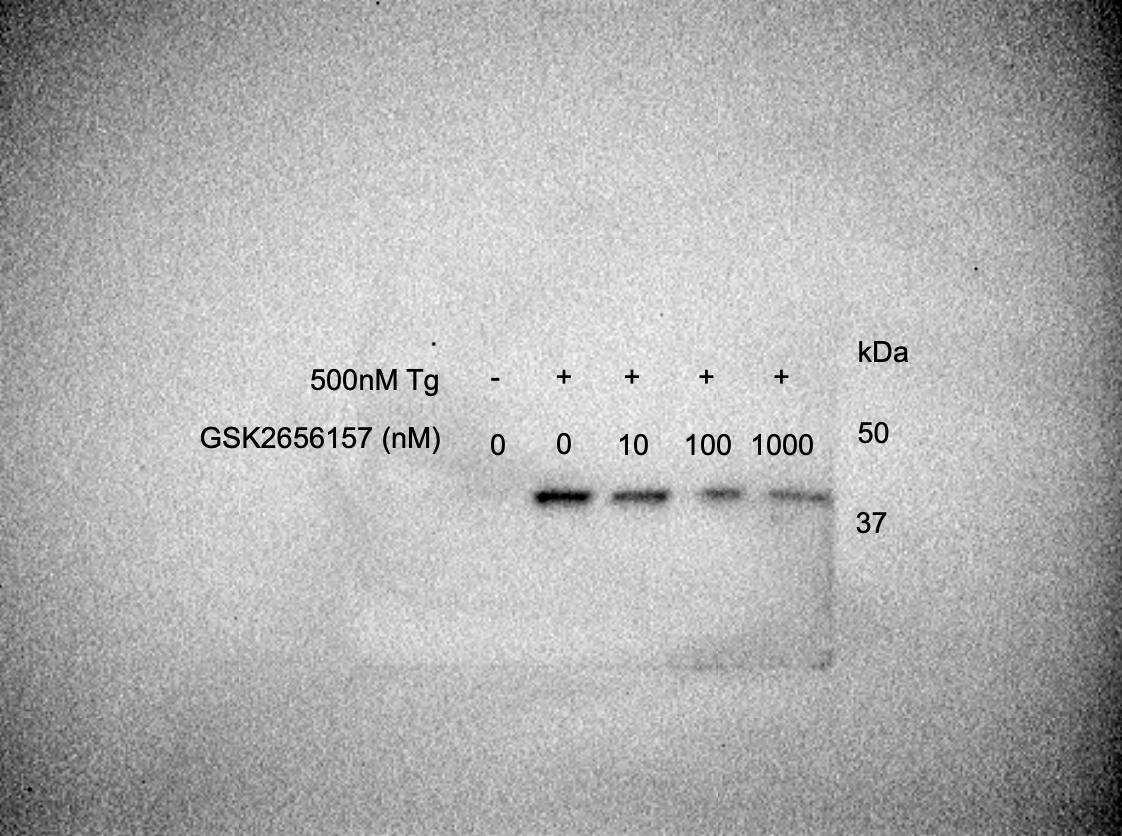

Supplement: Supplementary file 5 — Source data Fig. 4 [file 44318_2024_338_MOESM5_ESM.zip › SD figure 4/4C/eIF2alpha P WB.tif]

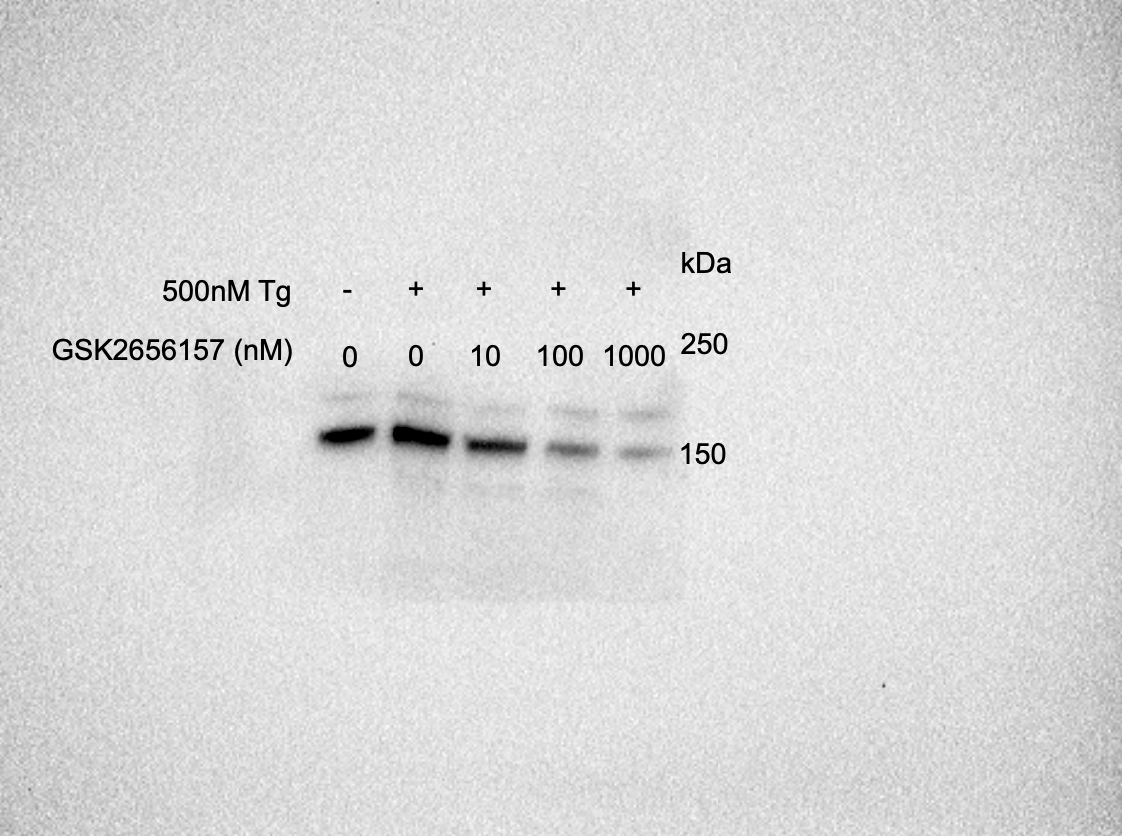

Supplement: Supplementary file 5 — Source data Fig. 4 [file 44318_2024_338_MOESM5_ESM.zip › SD figure 4/4C/MET WB.tif]

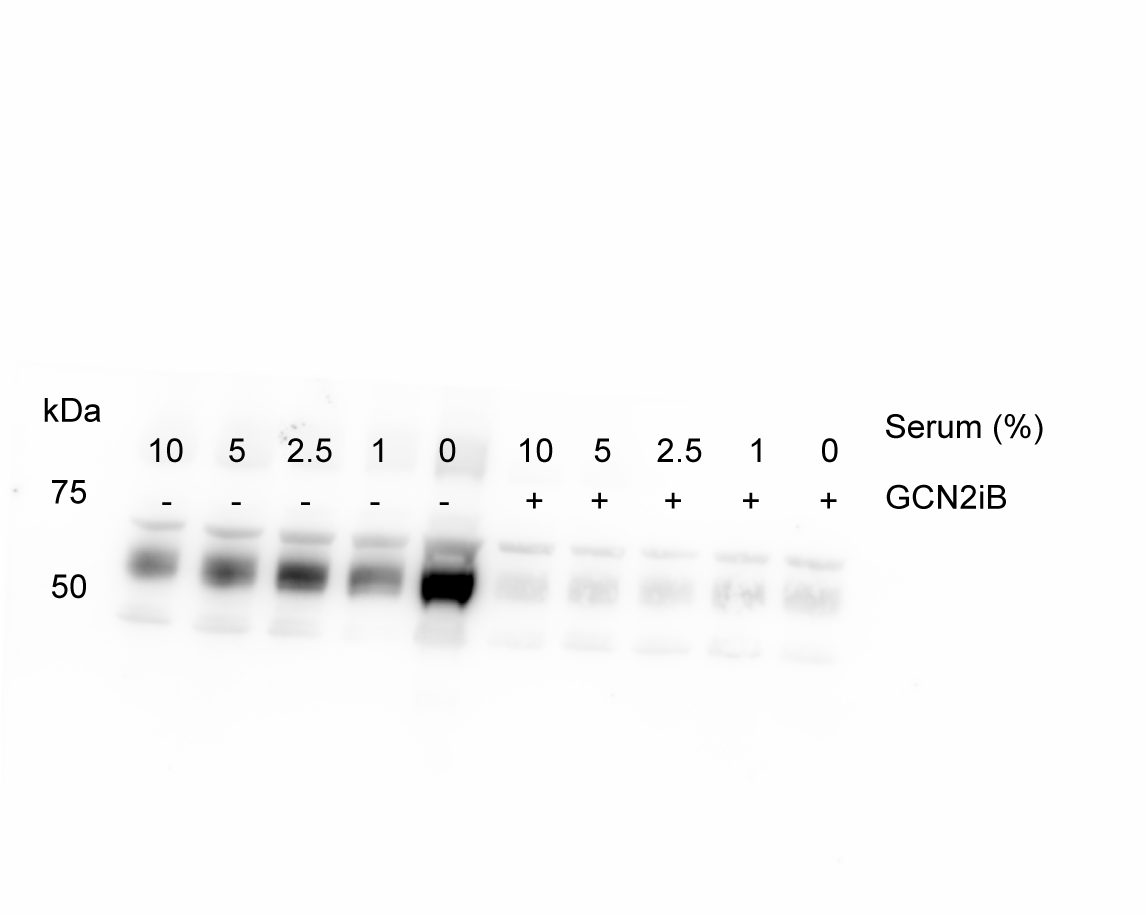

Supplement: Supplementary file 5 — Source data Fig. 4 [file 44318_2024_338_MOESM5_ESM.zip › SD figure 4/4A/ATF4 WB.tif]

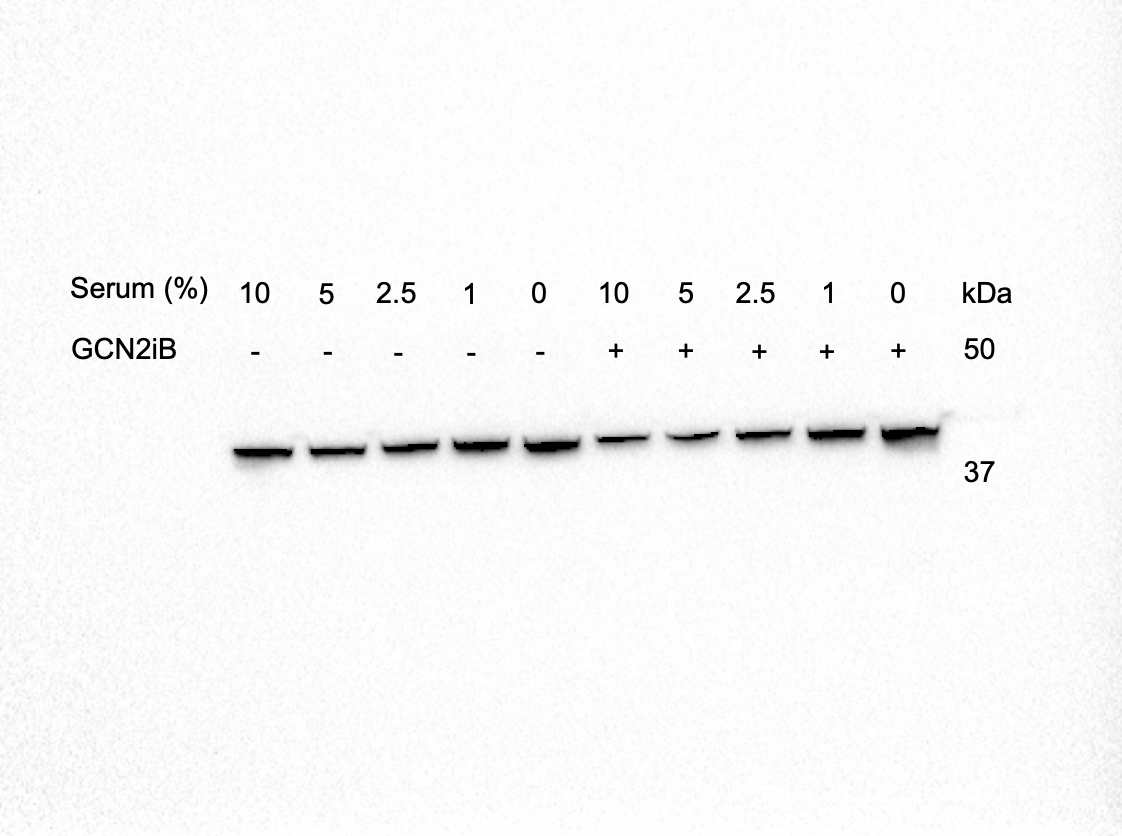

Supplement: Supplementary file 5 — Source data Fig. 4 [file 44318_2024_338_MOESM5_ESM.zip › SD figure 4/4A/TBP WB.tif]

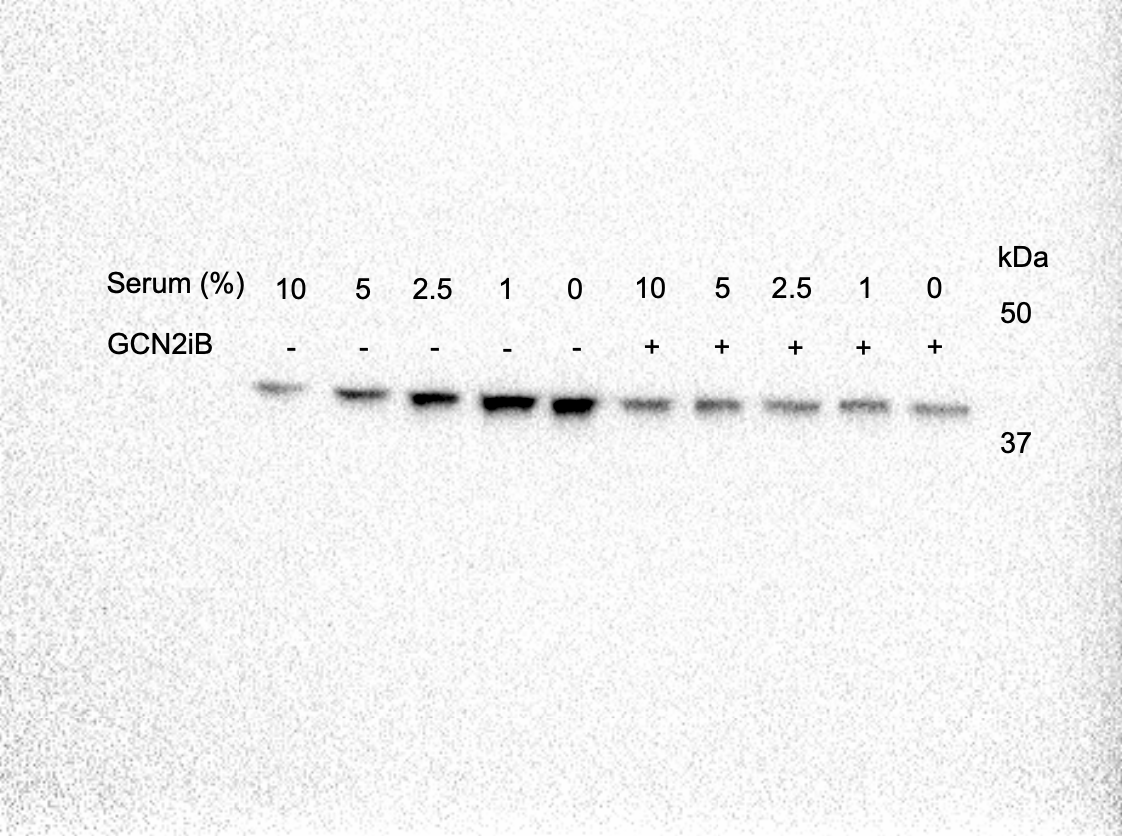

Supplement: Supplementary file 5 — Source data Fig. 4 [file 44318_2024_338_MOESM5_ESM.zip › SD figure 4/4A/eIF2a-P WB.tif]

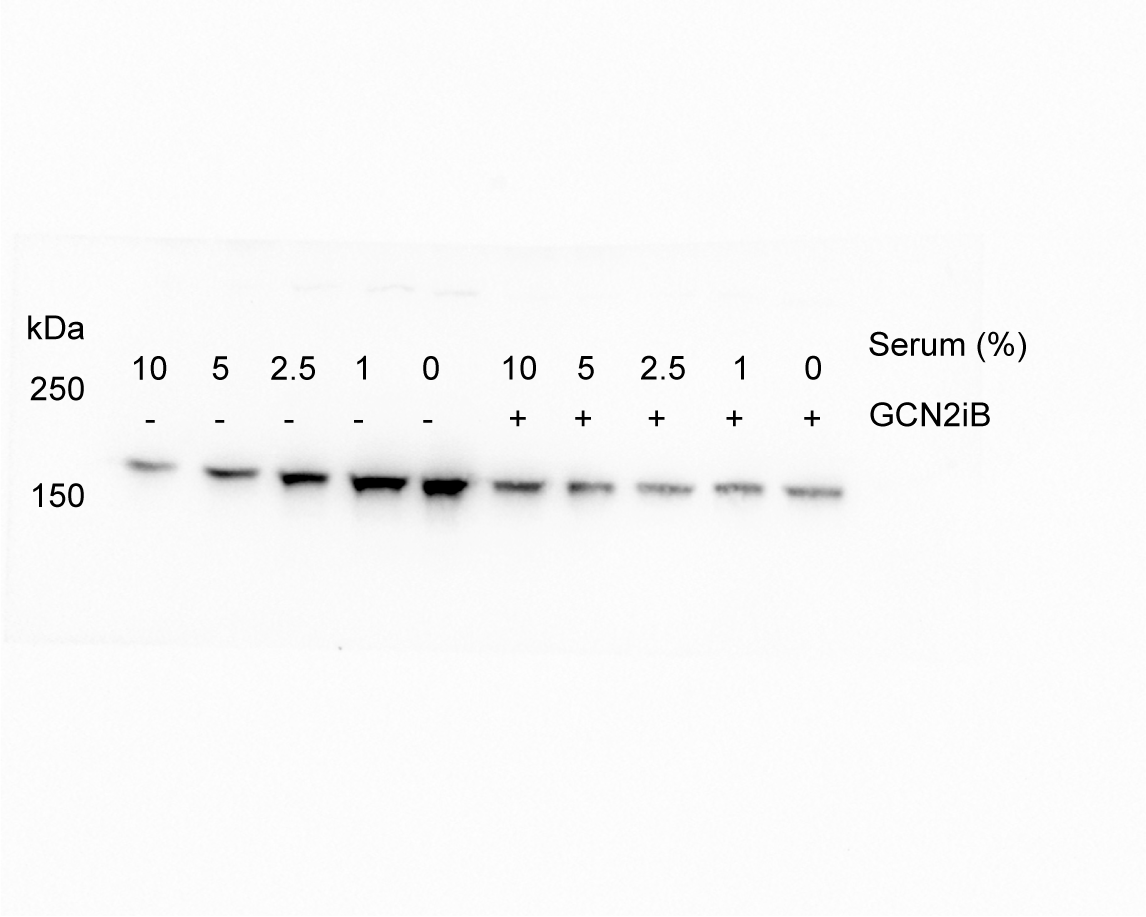

Supplement: Supplementary file 5 — Source data Fig. 4 [file 44318_2024_338_MOESM5_ESM.zip › SD figure 4/4A/MET WB.tif]

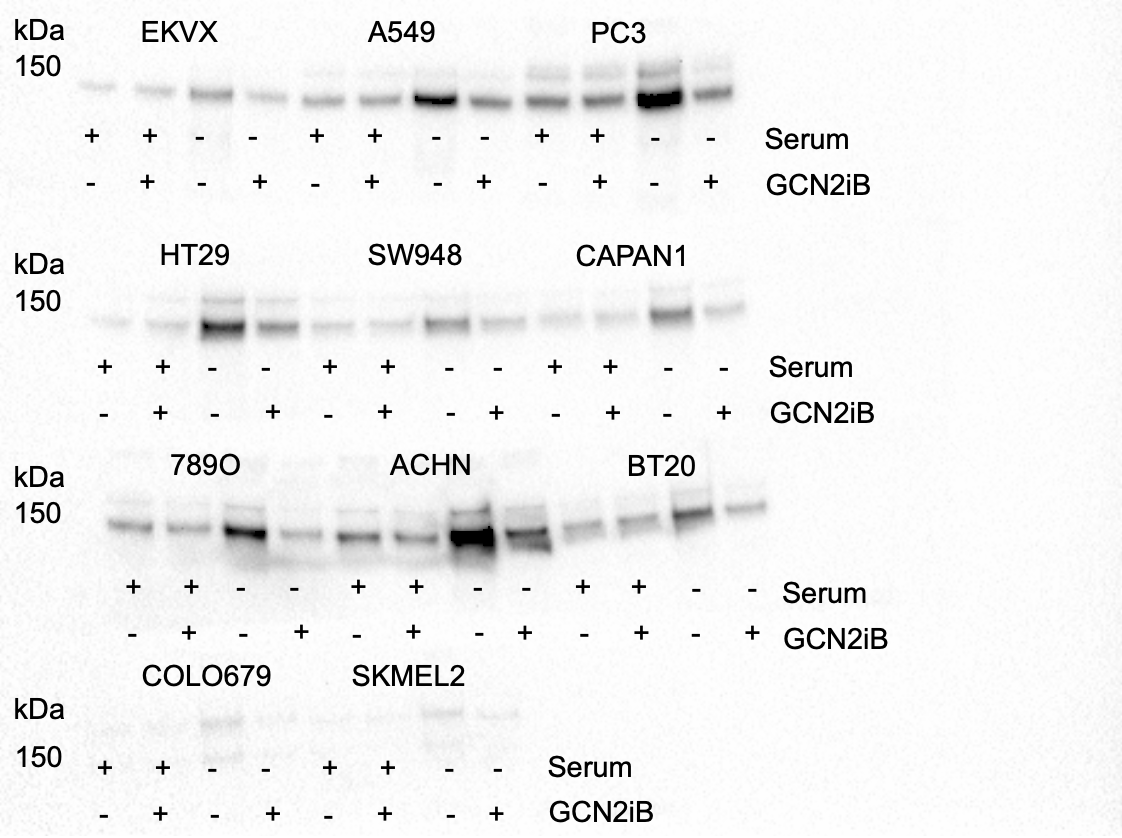

Supplement: Supplementary file 5 — Source data Fig. 4 [file 44318_2024_338_MOESM5_ESM.zip › SD figure 4/4G/MET WB low exposure.tif]

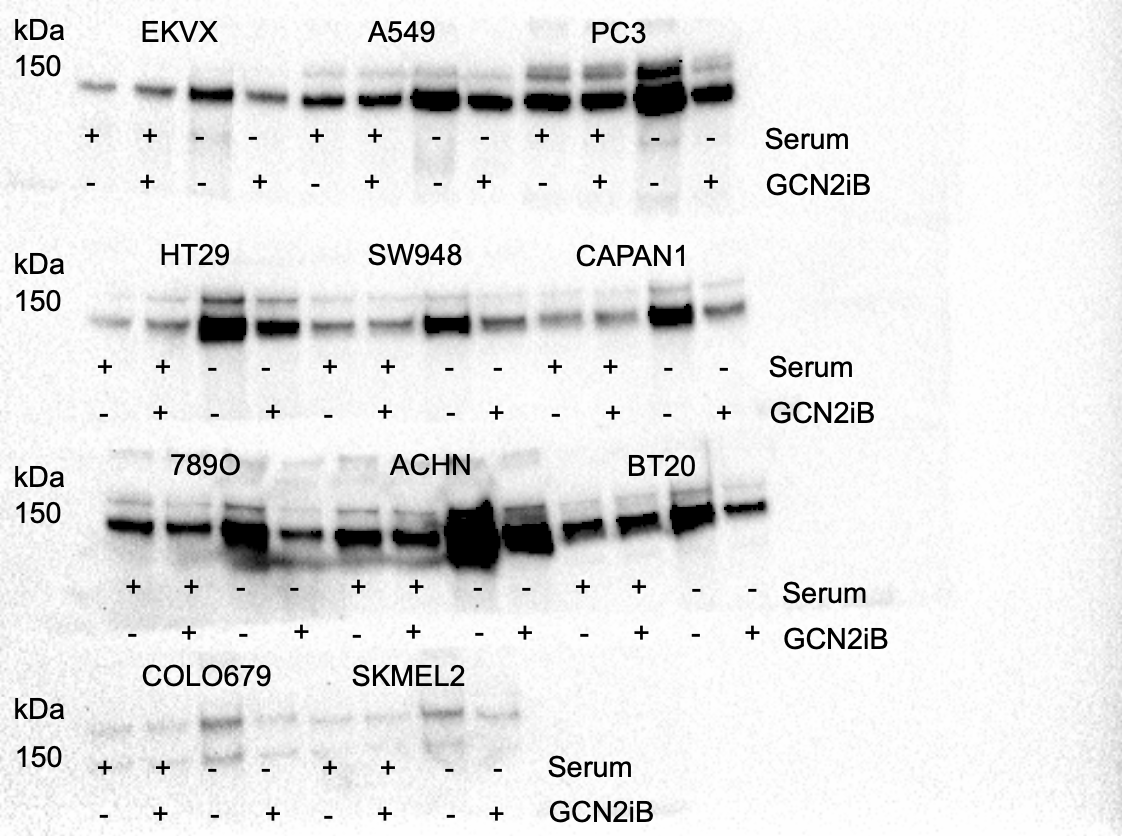

Supplement: Supplementary file 5 — Source data Fig. 4 [file 44318_2024_338_MOESM5_ESM.zip › SD figure 4/4G/MET WB high exposure.tif]

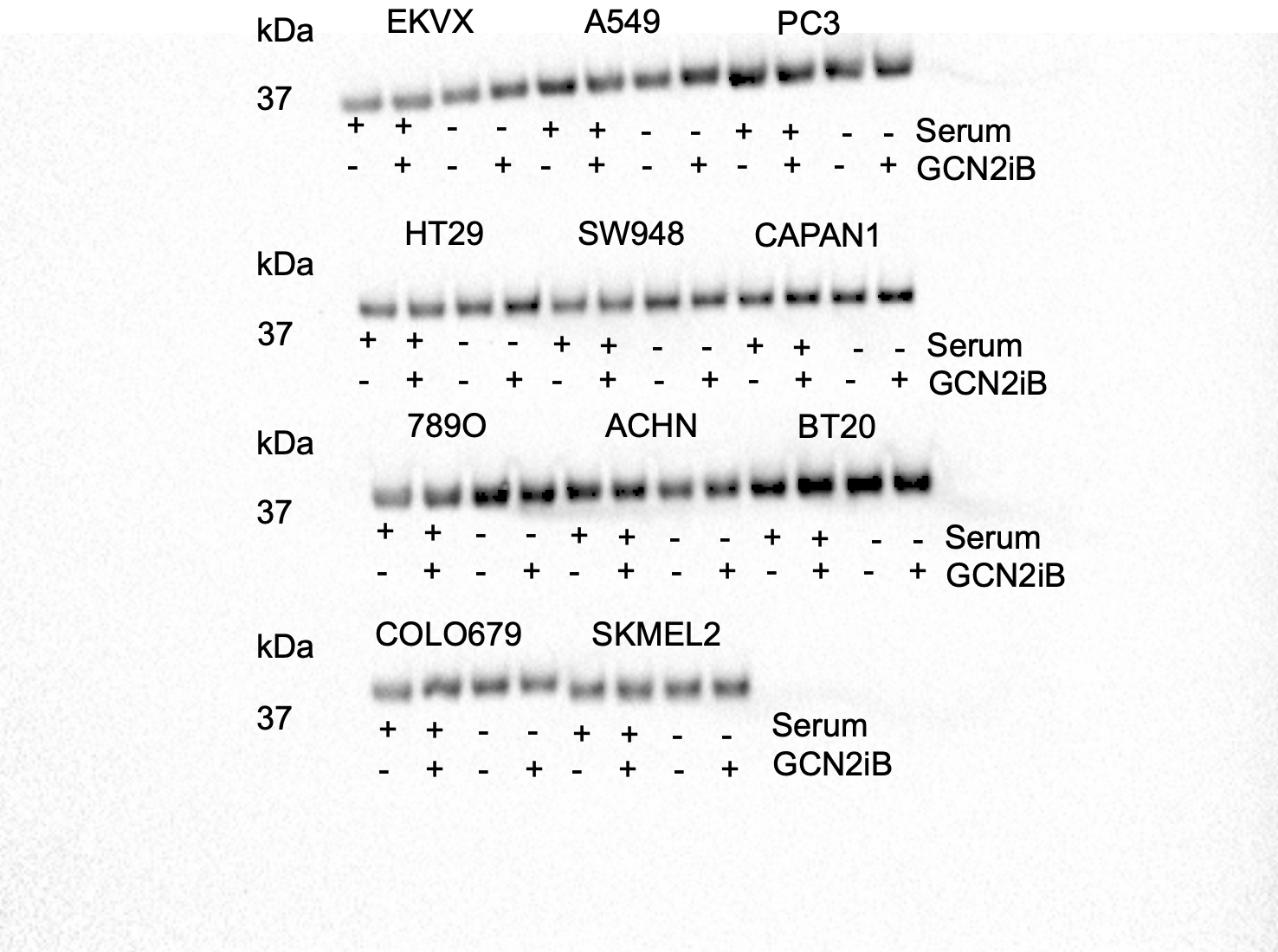

Supplement: Supplementary file 5 — Source data Fig. 4 [file 44318_2024_338_MOESM5_ESM.zip › SD figure 4/4G/TBP WB.tif]

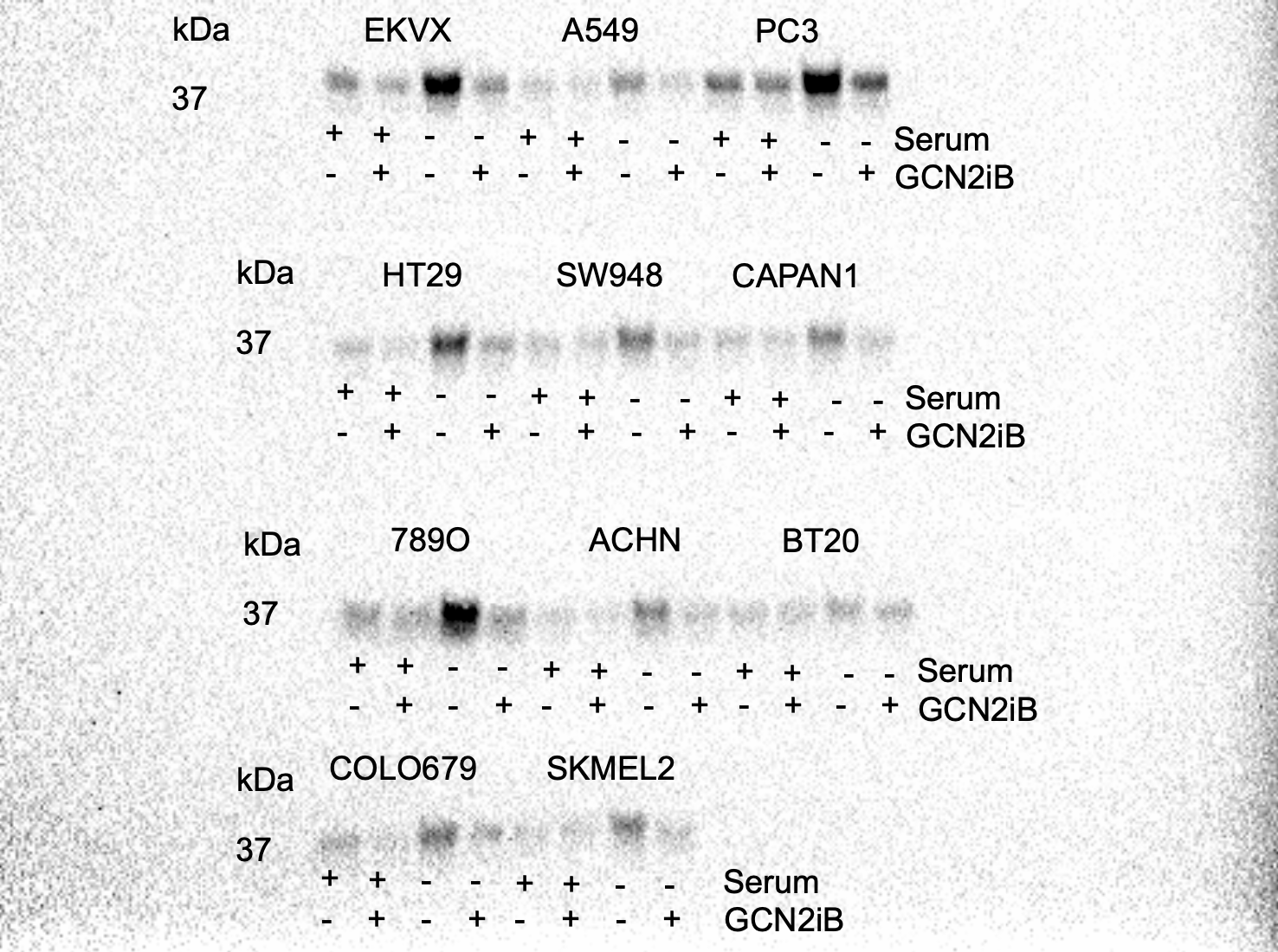

Supplement: Supplementary file 5 — Source data Fig. 4 [file 44318_2024_338_MOESM5_ESM.zip › SD figure 4/4G/eIF2alpha p WB.tif]

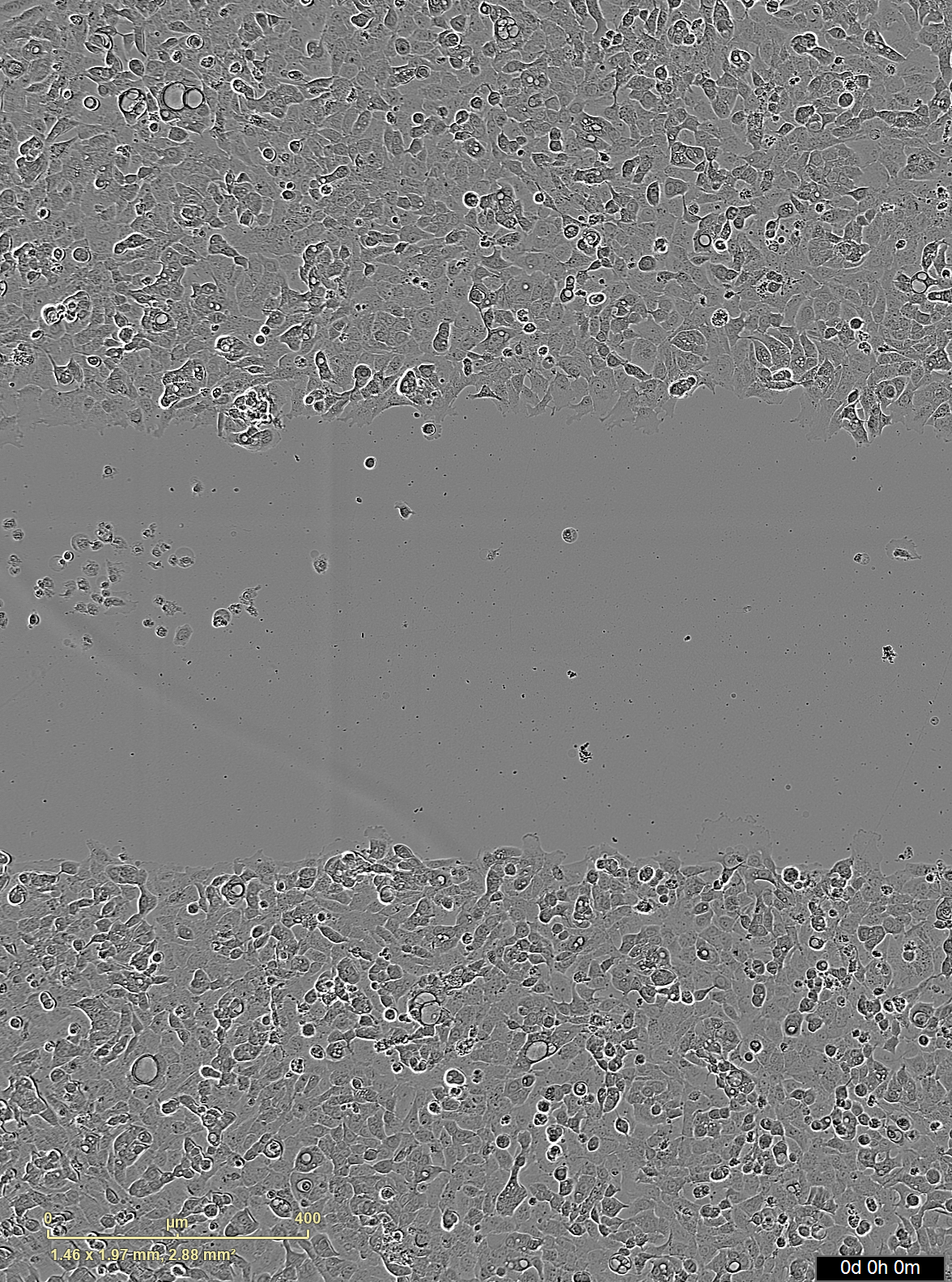

Supplement: Supplementary file 6 — Source data Fig. 5 [file 44318_2024_338_MOESM6_ESM.zip › SD figure 5/5C/No HGF 500nM GCN2iB 0h.tif]

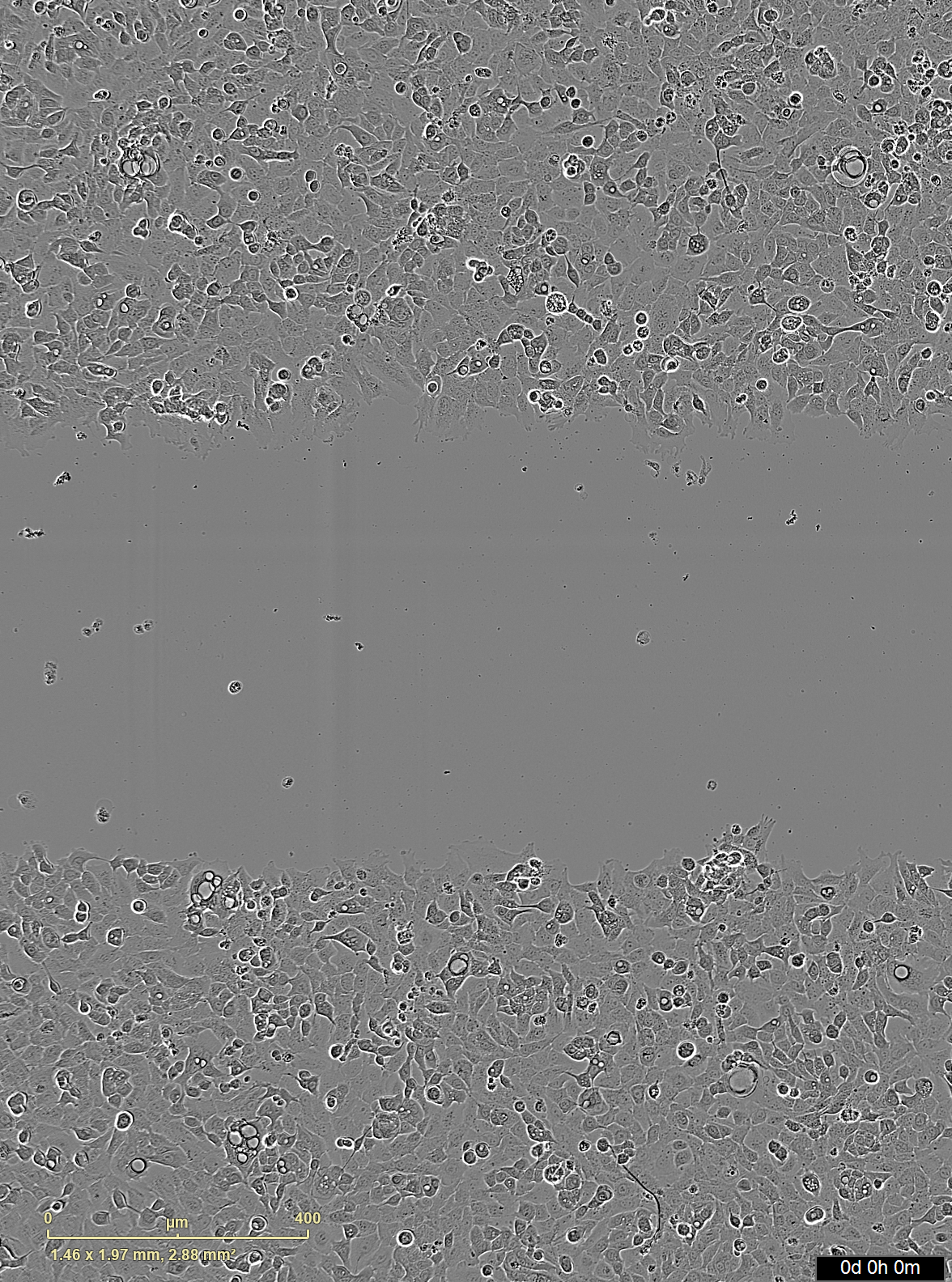

Supplement: Supplementary file 6 — Source data Fig. 5 [file 44318_2024_338_MOESM6_ESM.zip › SD figure 5/5C/No HGF No ISR inhibition 0h.tif]

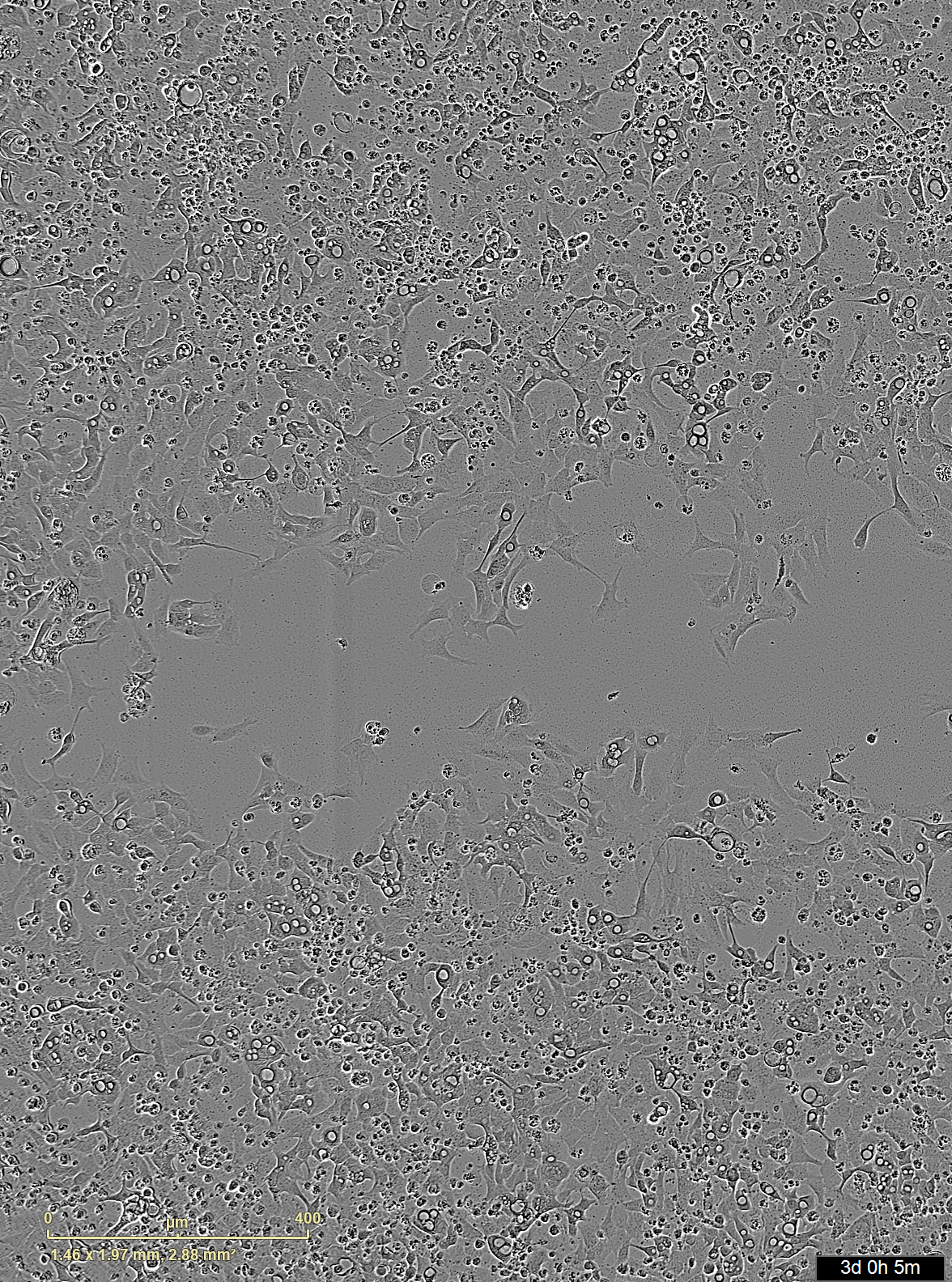

Supplement: Supplementary file 6 — Source data Fig. 5 [file 44318_2024_338_MOESM6_ESM.zip › SD figure 5/5C/50ng:ml HGF No ISR inhibition 48h.tif]

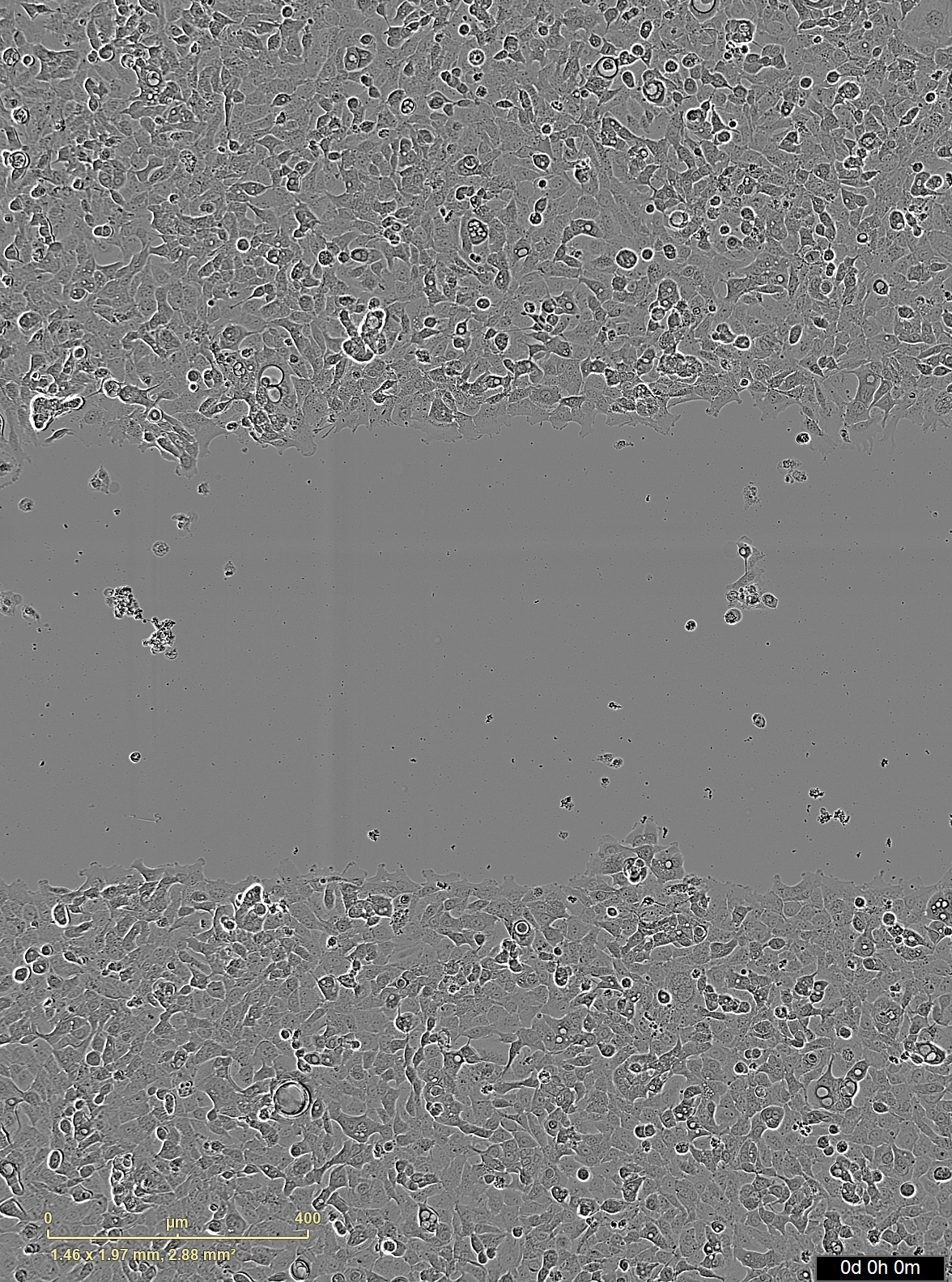

Supplement: Supplementary file 6 — Source data Fig. 5 [file 44318_2024_338_MOESM6_ESM.zip › SD figure 5/5C/50ng:ml HGF No ISR inhibition 0h.tif]

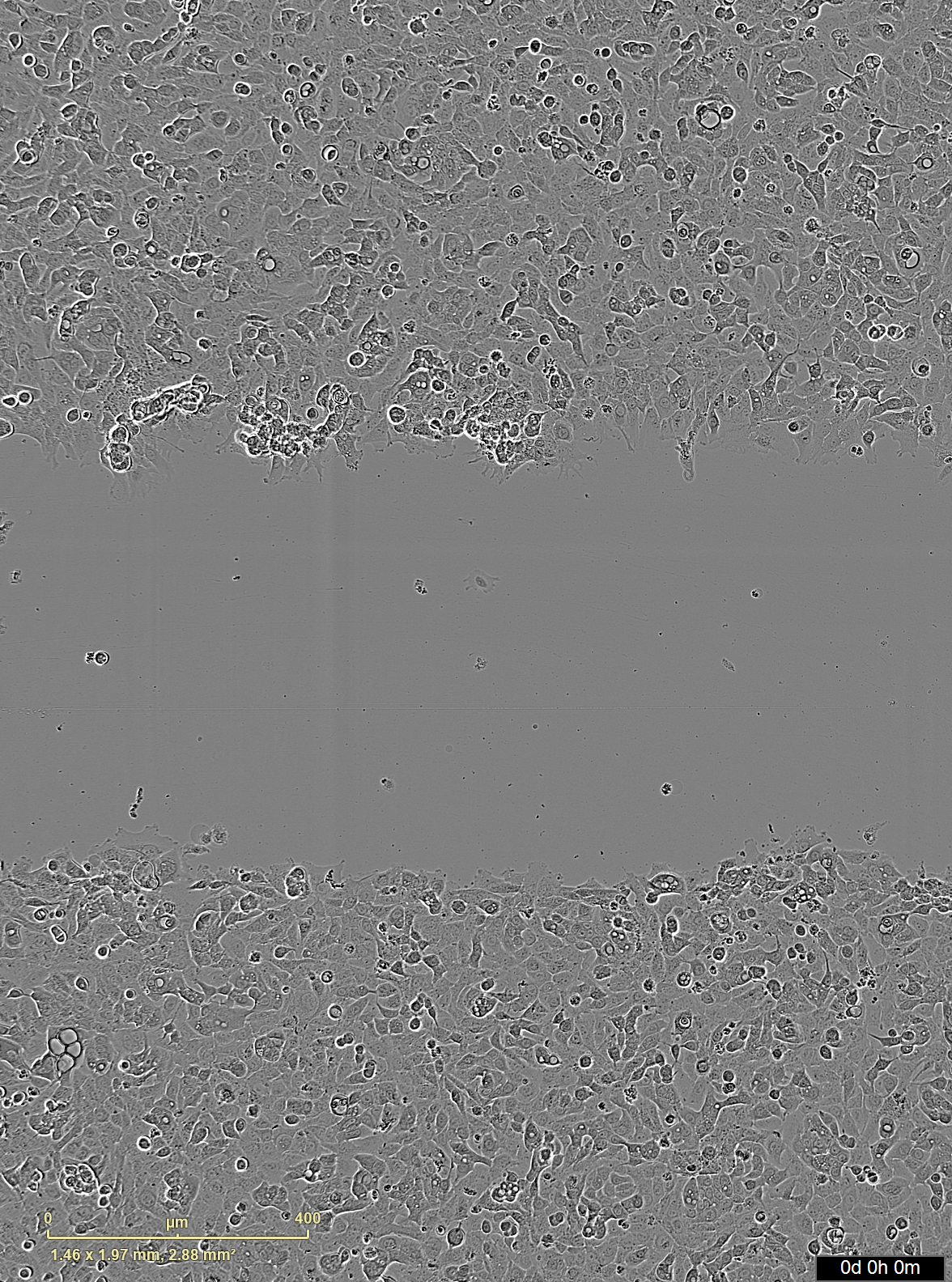

Supplement: Supplementary file 6 — Source data Fig. 5 [file 44318_2024_338_MOESM6_ESM.zip › SD figure 5/5C/50ng:ml HGF 500nM GCN2iB 0h.tif]

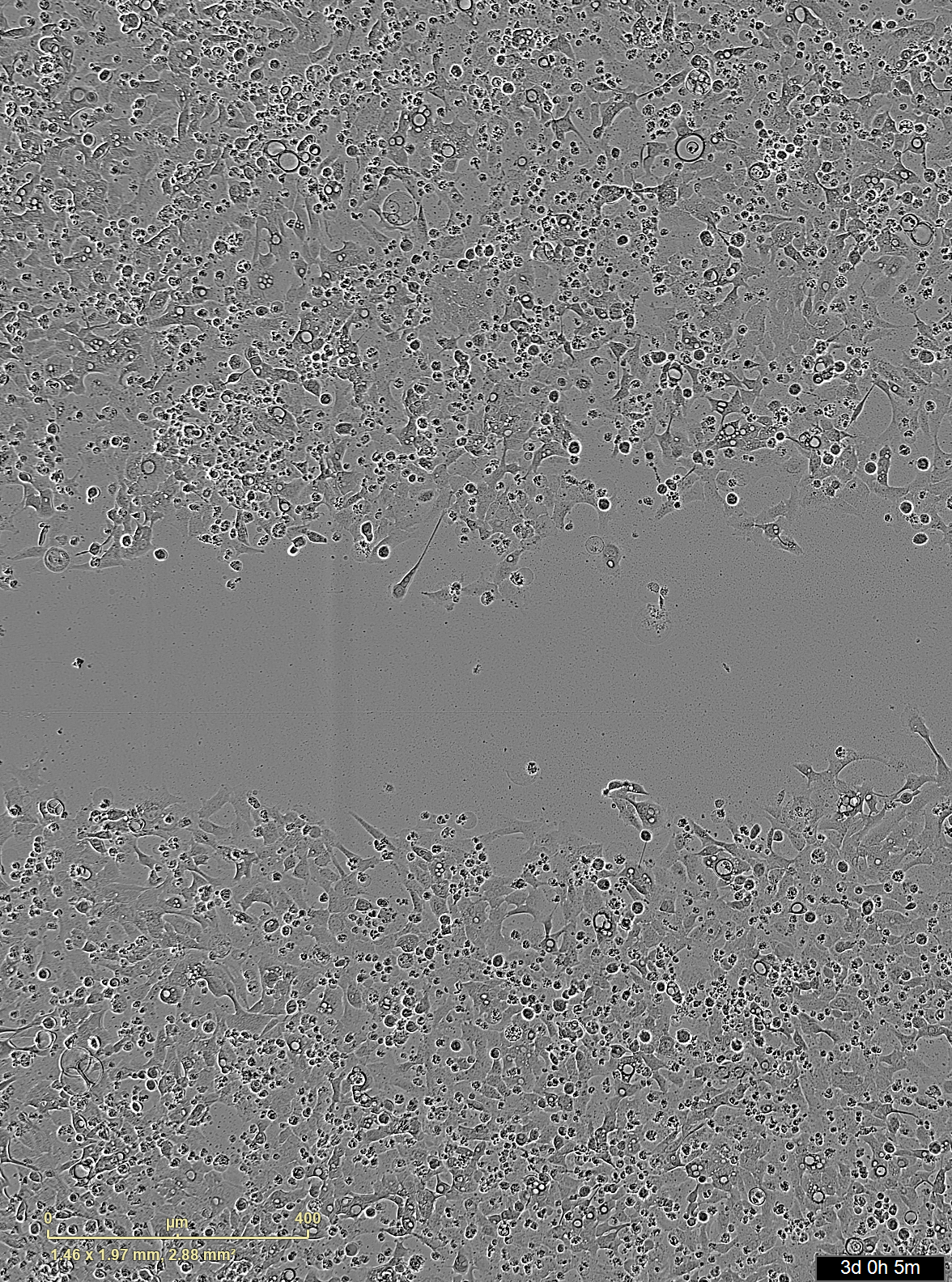

Supplement: Supplementary file 6 — Source data Fig. 5 [file 44318_2024_338_MOESM6_ESM.zip › SD figure 5/5C/50ng:ml HGF 500nM GCN2iB 48h.tif]

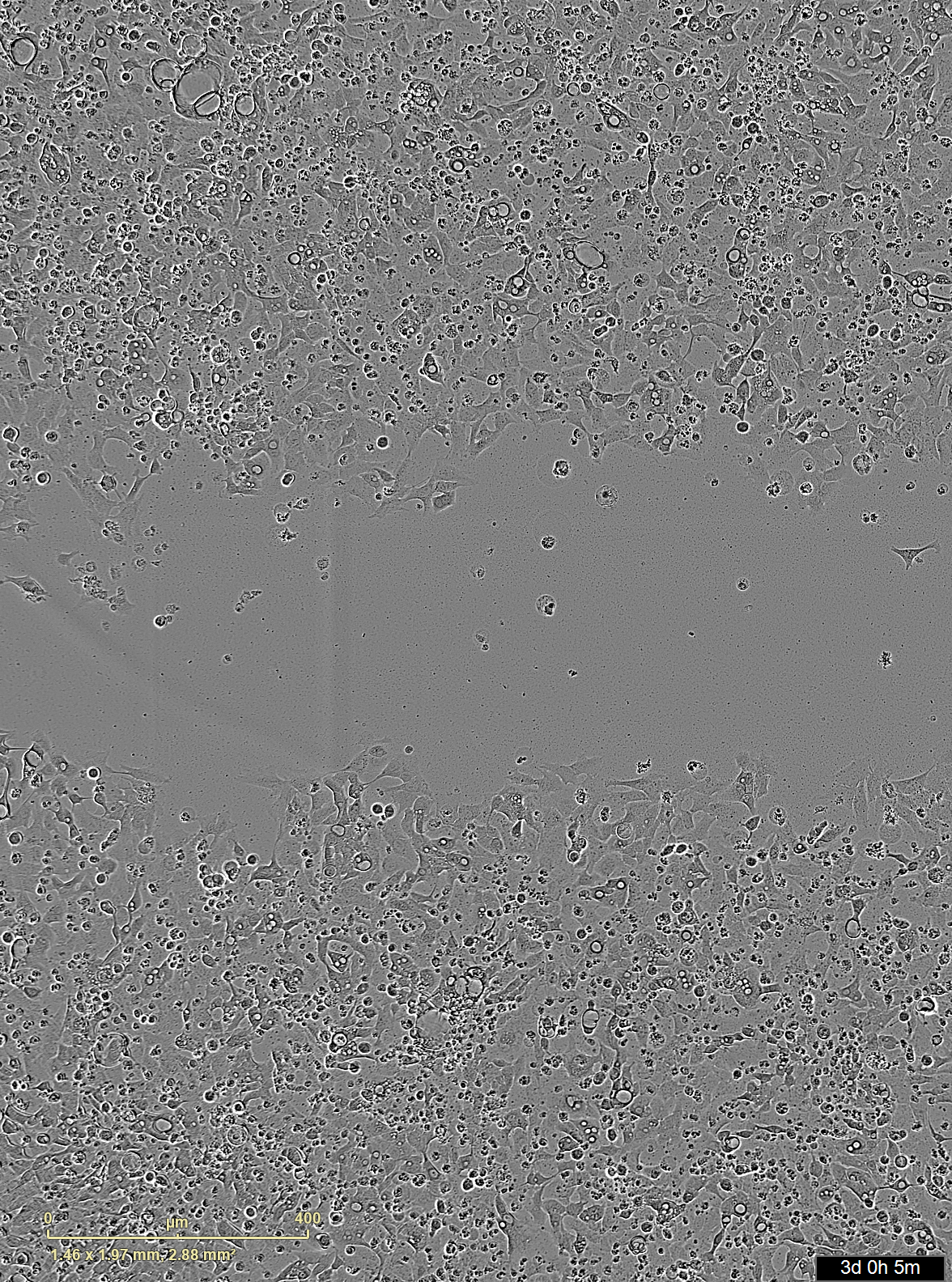

Supplement: Supplementary file 6 — Source data Fig. 5 [file 44318_2024_338_MOESM6_ESM.zip › SD figure 5/5C/No HGF 500nM GCN2iB 48h.tif]

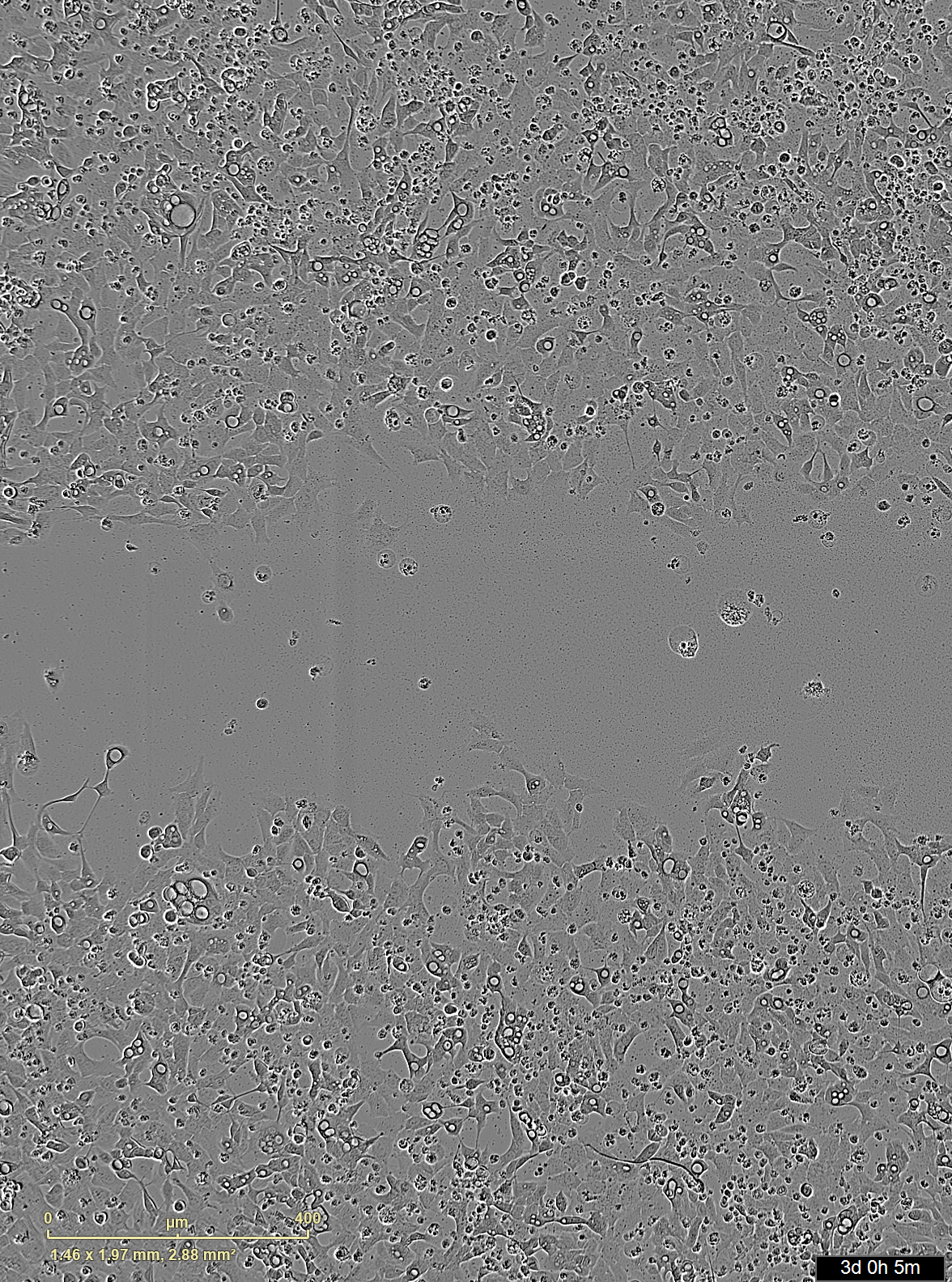

Supplement: Supplementary file 6 — Source data Fig. 5 [file 44318_2024_338_MOESM6_ESM.zip › SD figure 5/5C/No HGF No ISR inhibition 48h.tif]

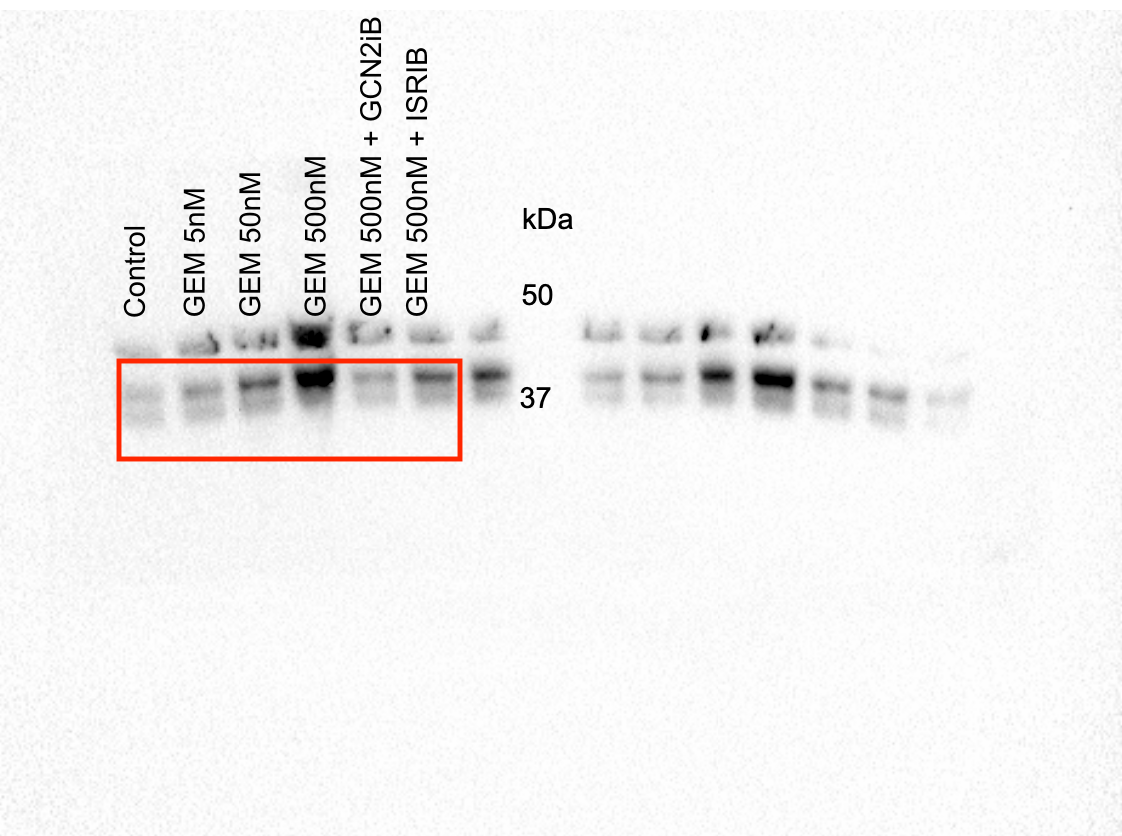

Supplement: Supplementary file 6 — Source data Fig. 5 [file 44318_2024_338_MOESM6_ESM.zip › SD figure 5/5E/eIF2alphaP WB.tif]

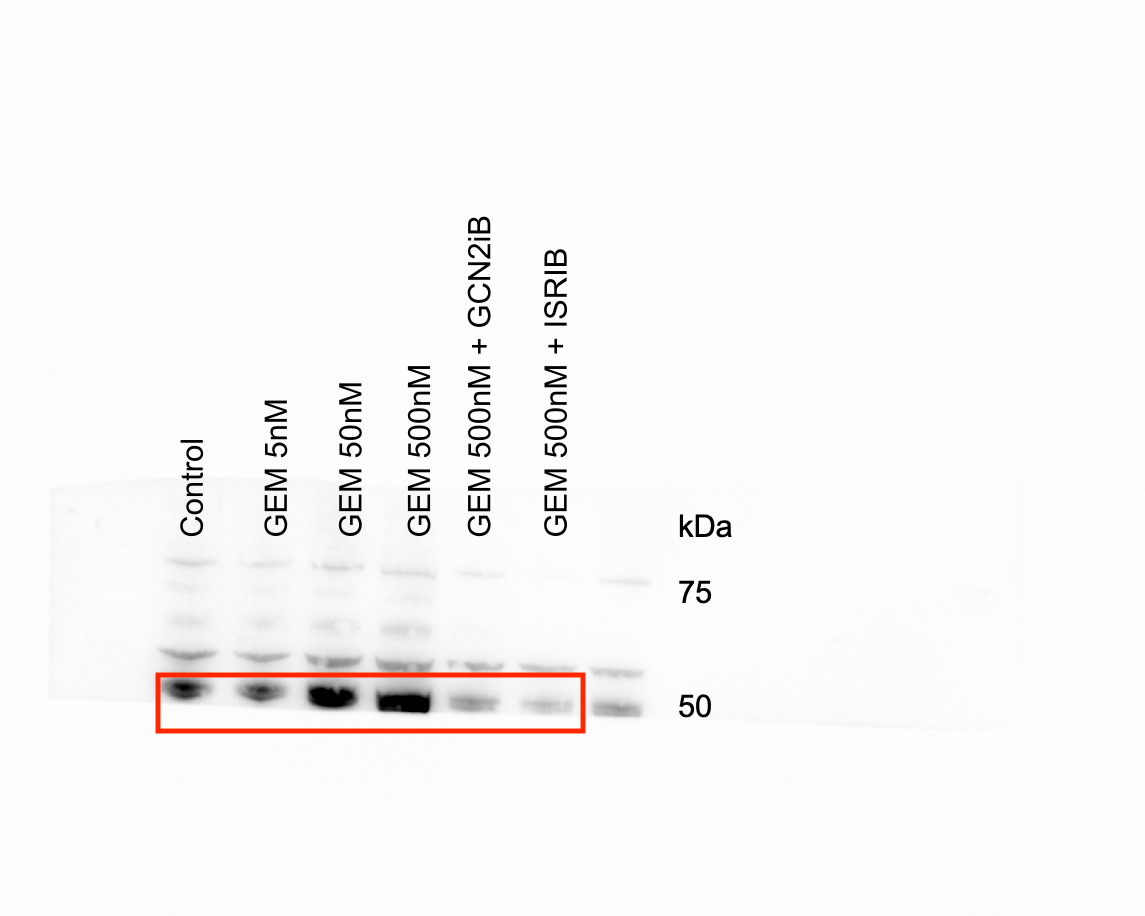

Supplement: Supplementary file 6 — Source data Fig. 5 [file 44318_2024_338_MOESM6_ESM.zip › SD figure 5/5E/ATF4 WB.tif]

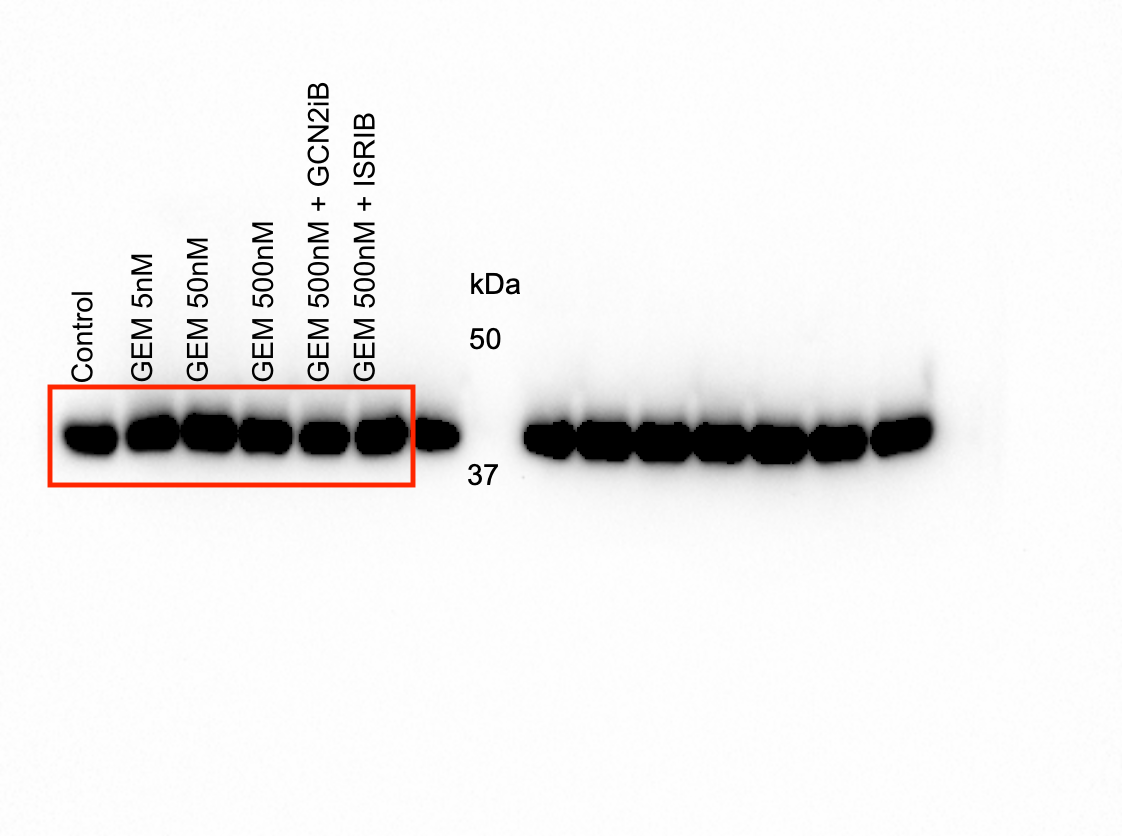

Supplement: Supplementary file 6 — Source data Fig. 5 [file 44318_2024_338_MOESM6_ESM.zip › SD figure 5/5E/TBP WB.tif]

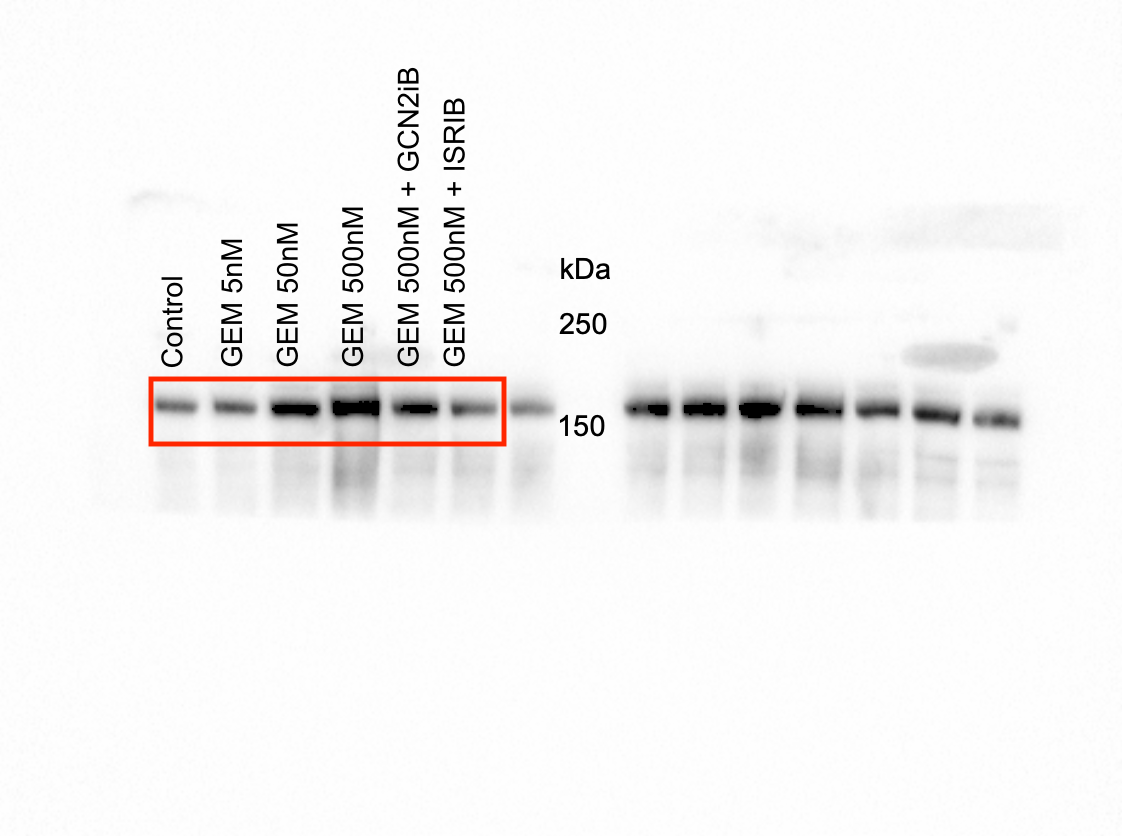

Supplement: Supplementary file 6 — Source data Fig. 5 [file 44318_2024_338_MOESM6_ESM.zip › SD figure 5/5E/MET WB.tif]

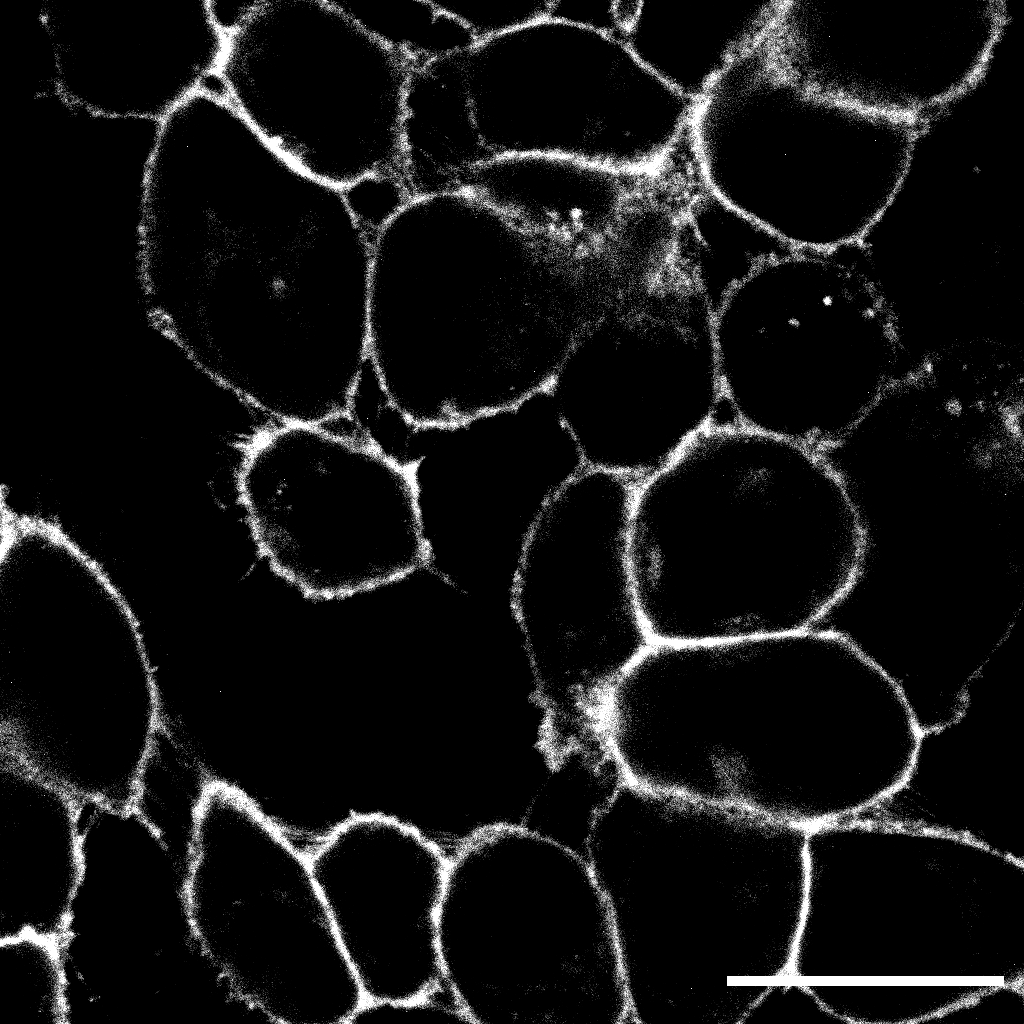

Supplement: Supplementary file 6 — Source data Fig. 5 [file 44318_2024_338_MOESM6_ESM.zip › SD figure 5/5B/FBS_HGF_CAD.png]

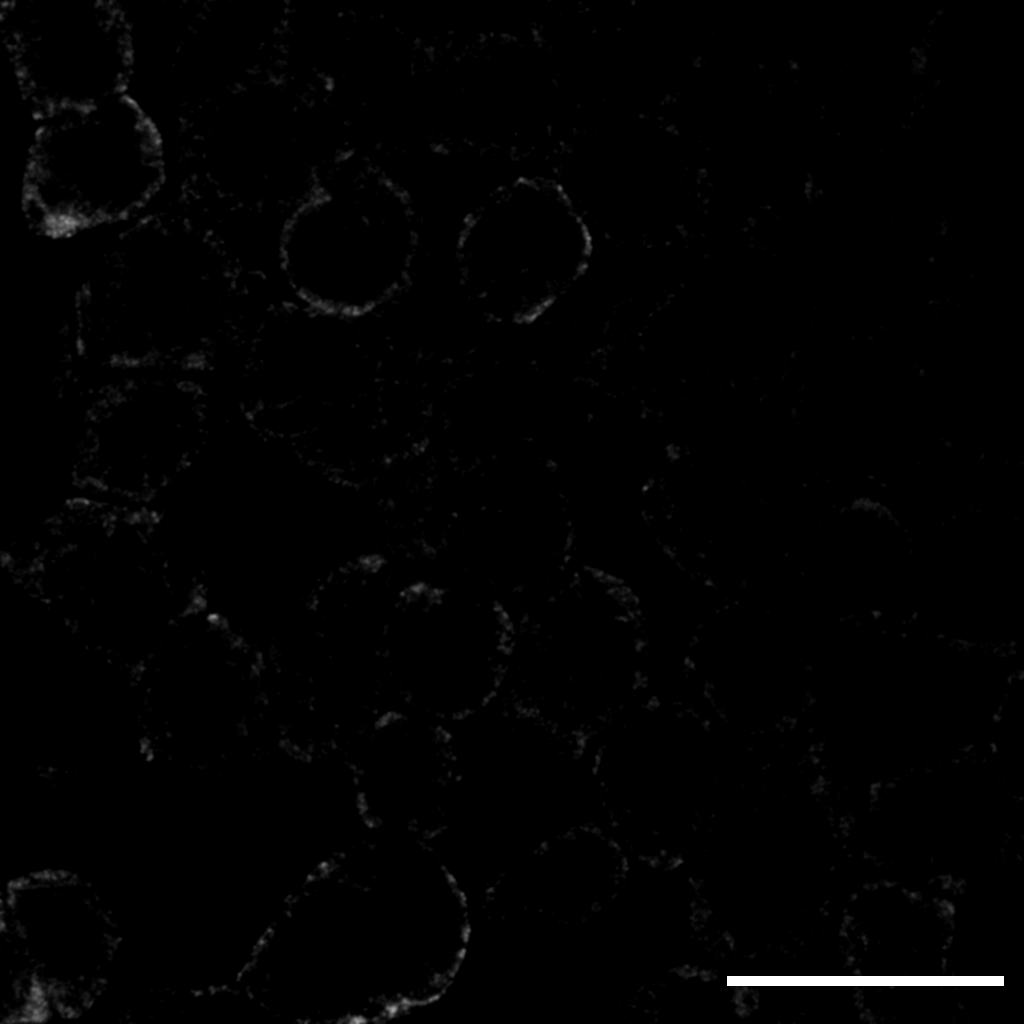

Supplement: Supplementary file 6 — Source data Fig. 5 [file 44318_2024_338_MOESM6_ESM.zip › SD figure 5/5B/FBS_ACTA2.png]

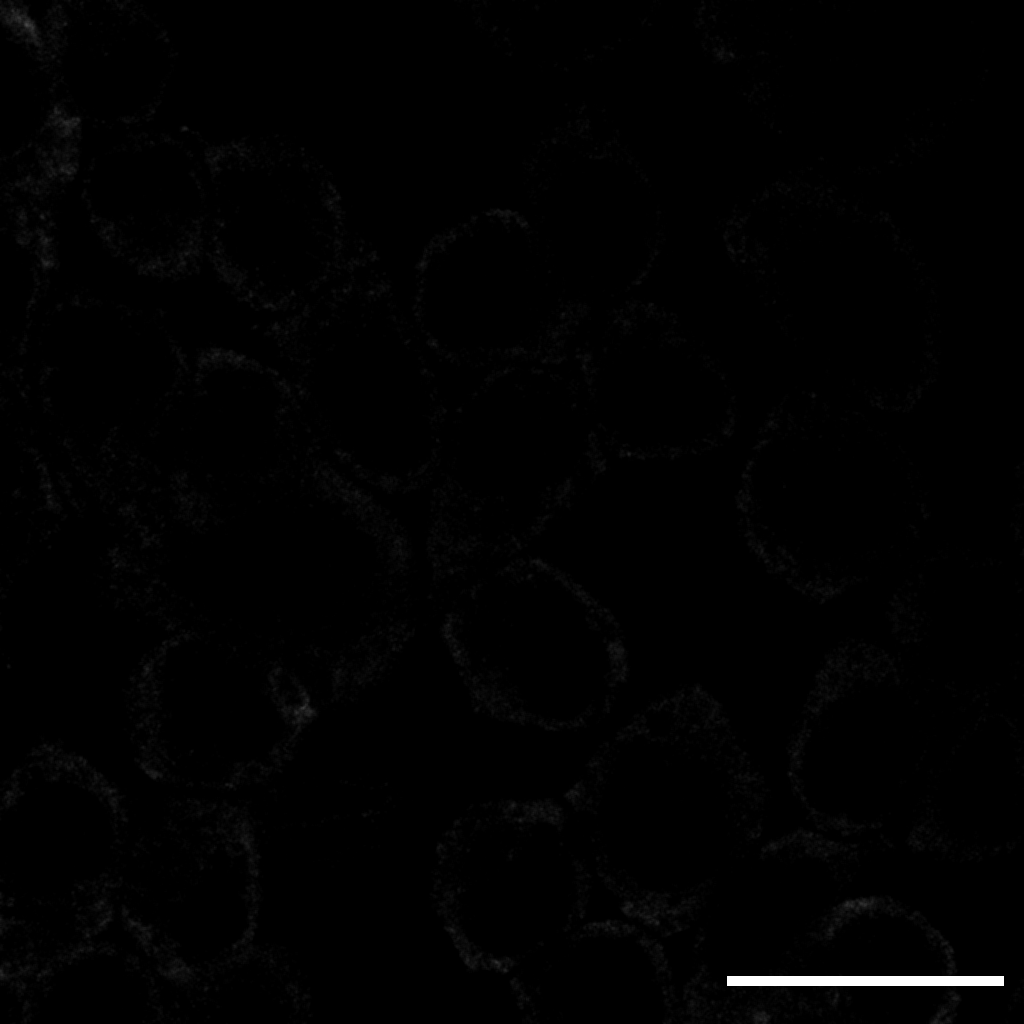

Supplement: Supplementary file 6 — Source data Fig. 5 [file 44318_2024_338_MOESM6_ESM.zip › SD figure 5/5B/SS_GCN2iB_ACTA2.png]

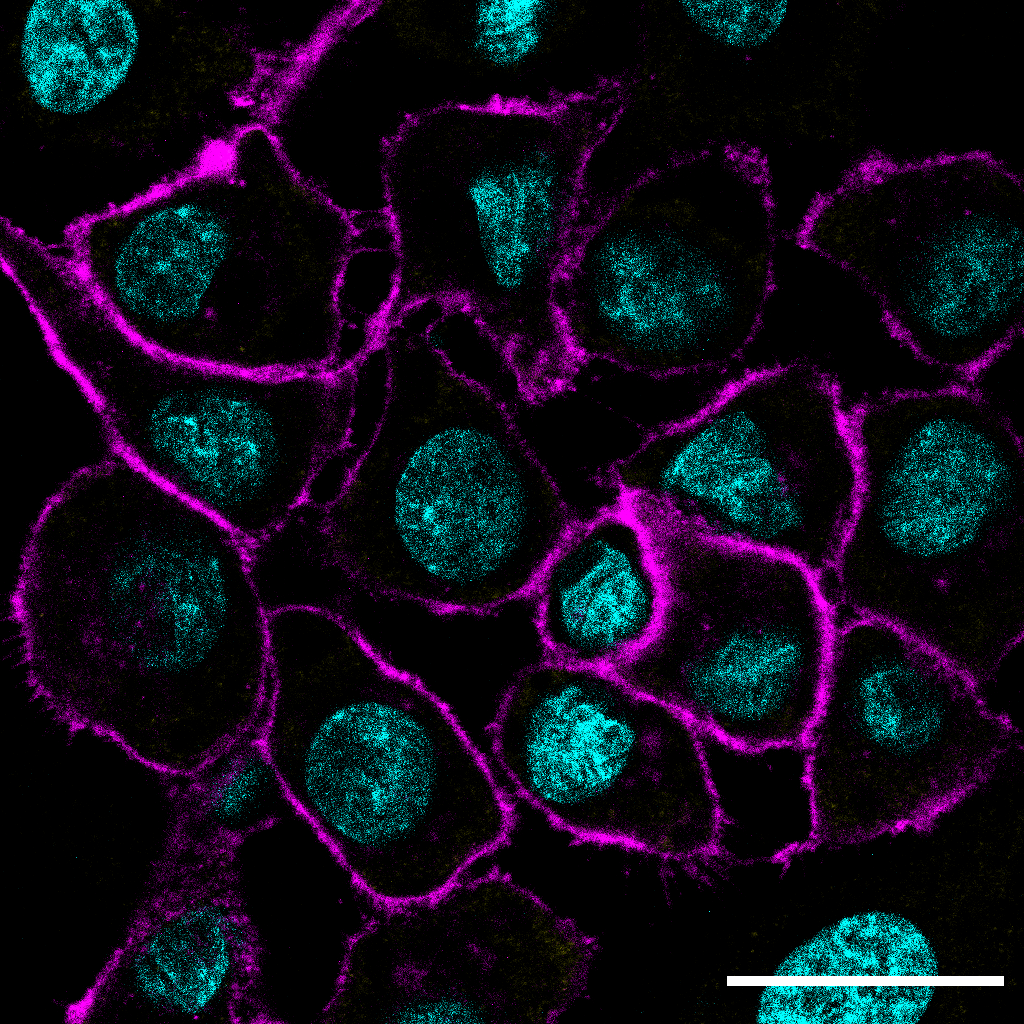

Supplement: Supplementary file 6 — Source data Fig. 5 [file 44318_2024_338_MOESM6_ESM.zip › SD figure 5/5B/SS_Merge.png]

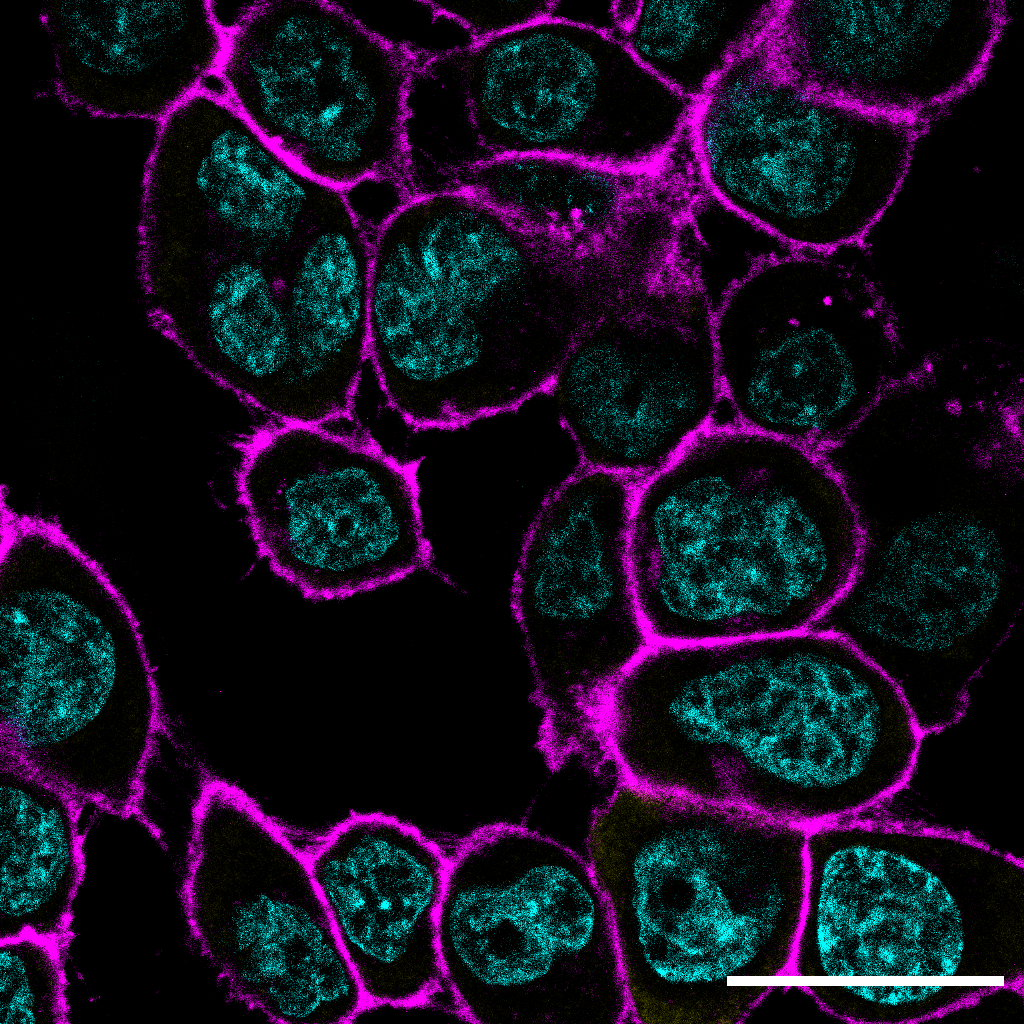

Supplement: Supplementary file 6 — Source data Fig. 5 [file 44318_2024_338_MOESM6_ESM.zip › SD figure 5/5B/FBS_HGF_Merge.png]

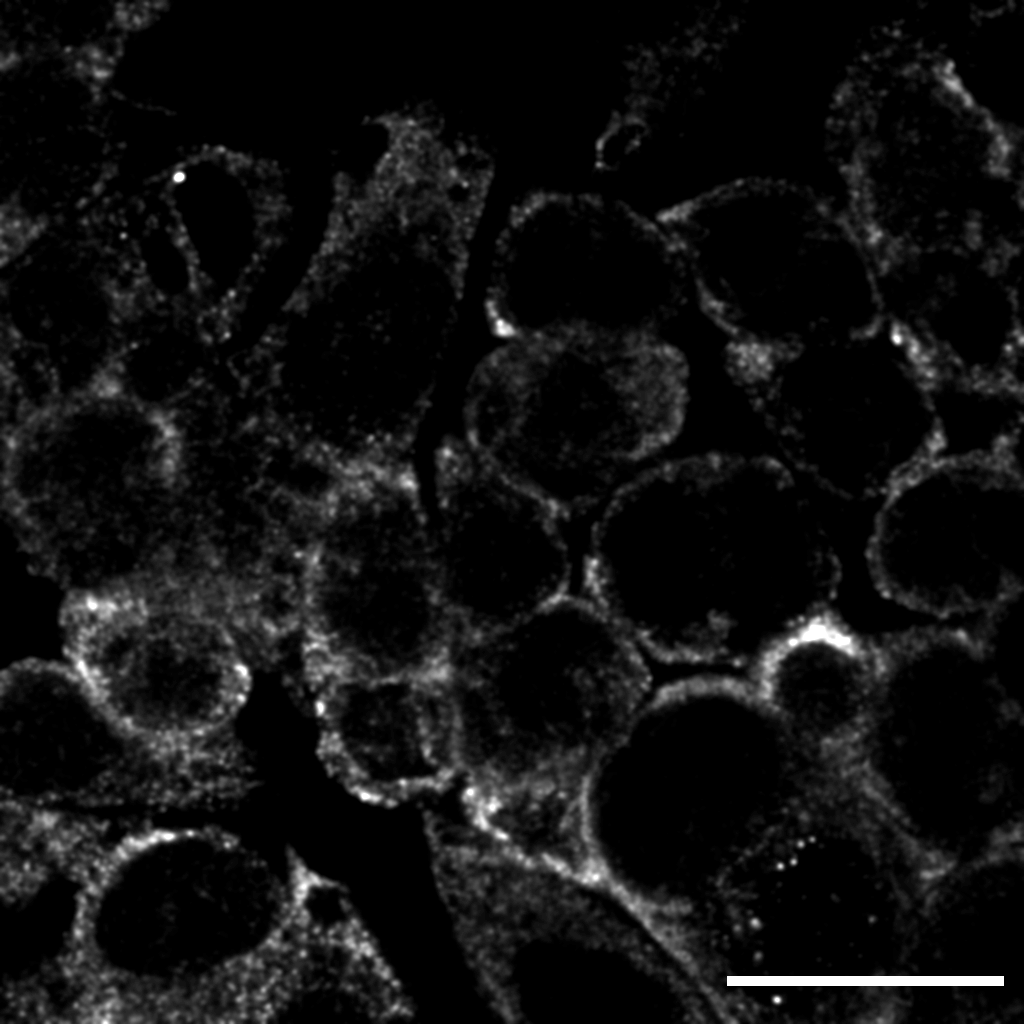

Supplement: Supplementary file 6 — Source data Fig. 5 [file 44318_2024_338_MOESM6_ESM.zip › SD figure 5/5B/SS_HGF_ACTA2.png]

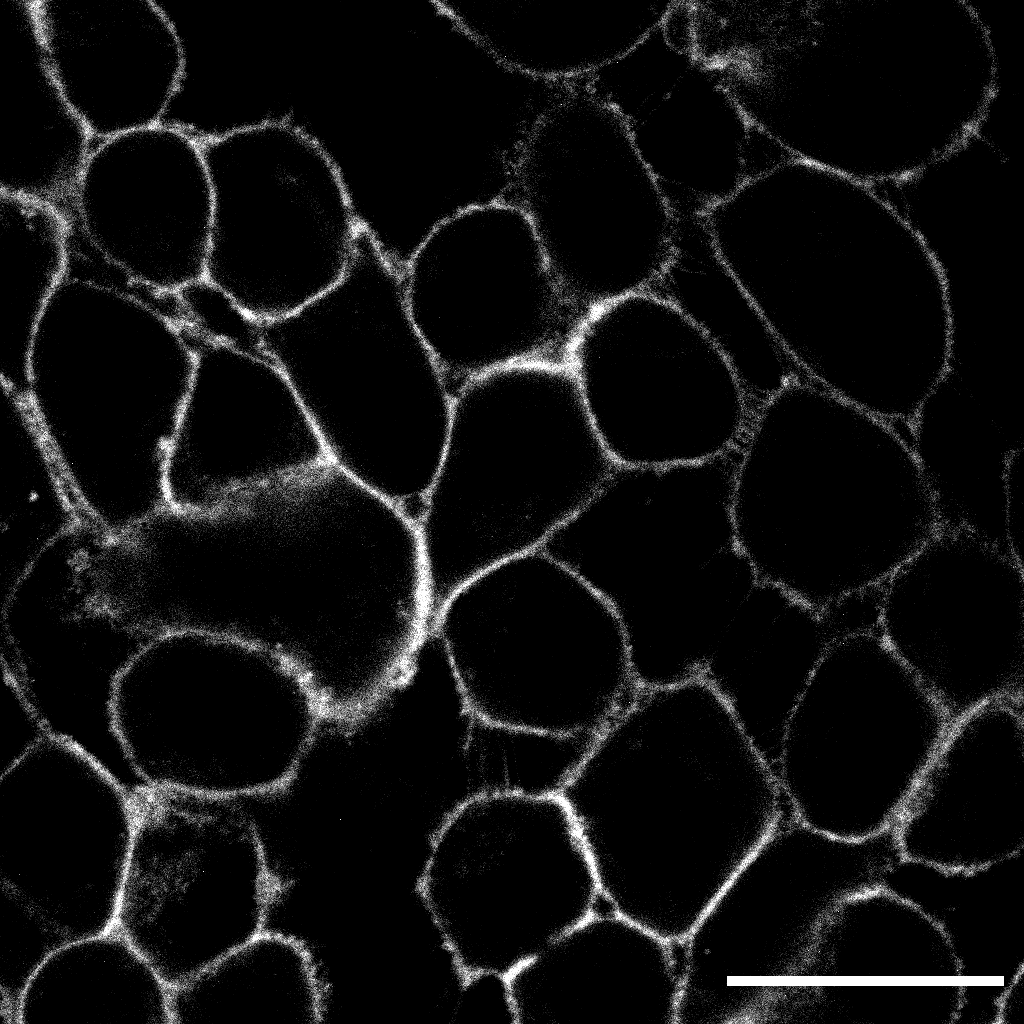

Supplement: Supplementary file 6 — Source data Fig. 5 [file 44318_2024_338_MOESM6_ESM.zip › SD figure 5/5B/SS_GCN2iB_CAD.png]

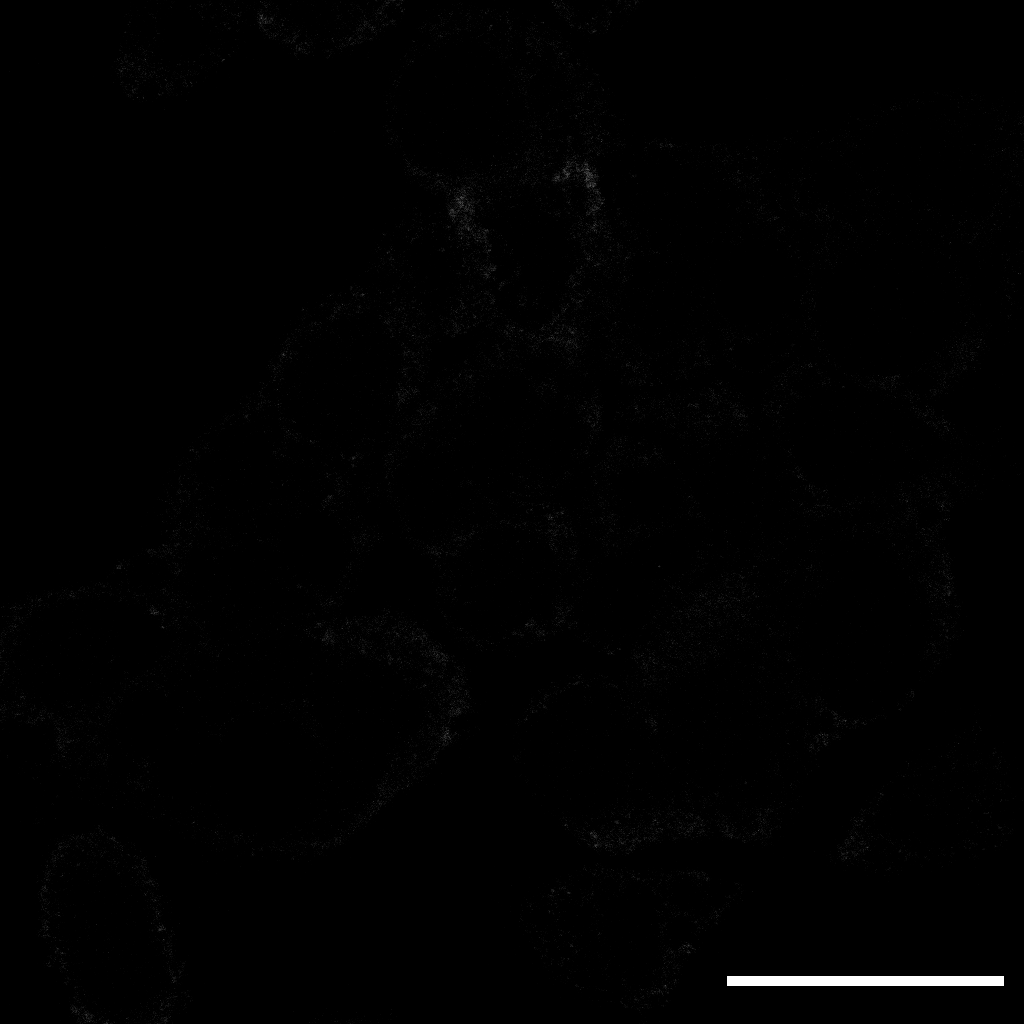

Supplement: Supplementary file 6 — Source data Fig. 5 [file 44318_2024_338_MOESM6_ESM.zip › SD figure 5/5B/SS_GCN2iB_HGF_ACTA2.png]

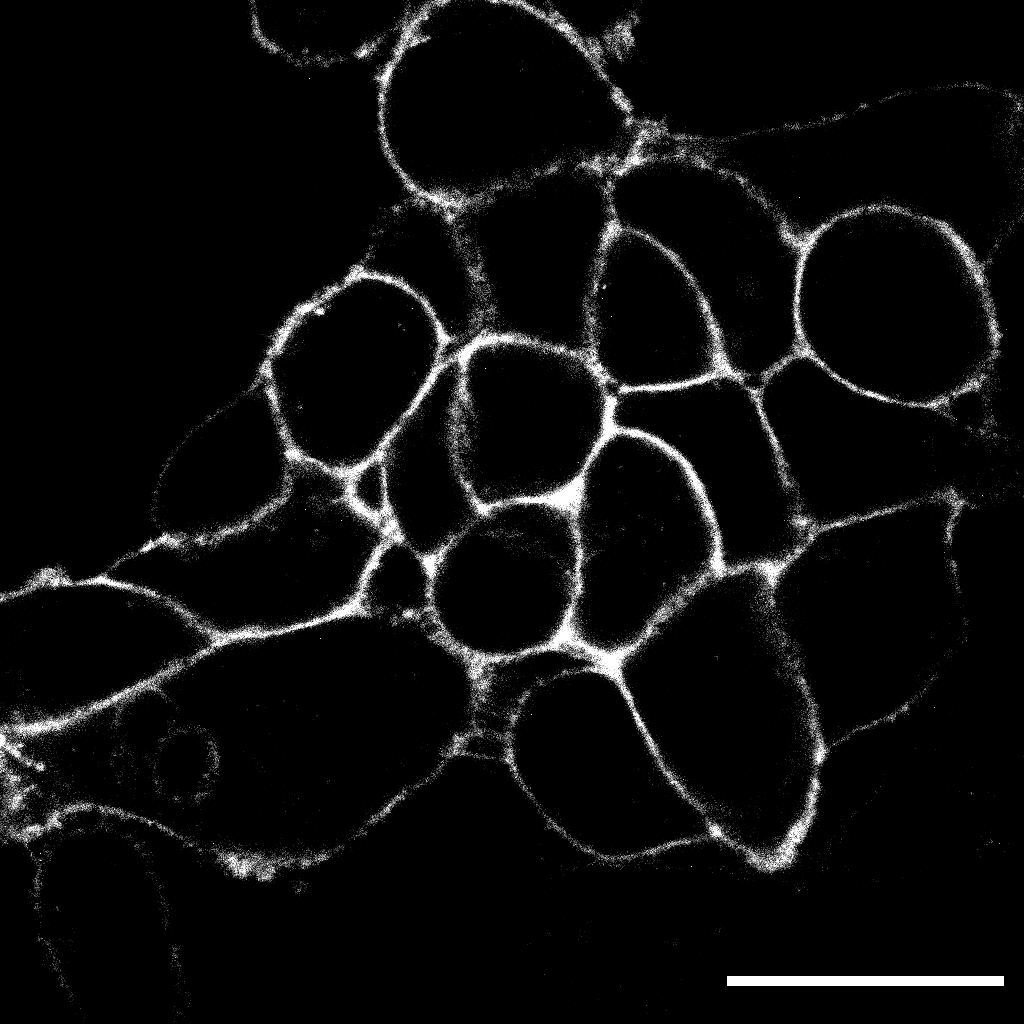

Supplement: Supplementary file 6 — Source data Fig. 5 [file 44318_2024_338_MOESM6_ESM.zip › SD figure 5/5B/SS_GCN2iB_HGF_CAD.png]

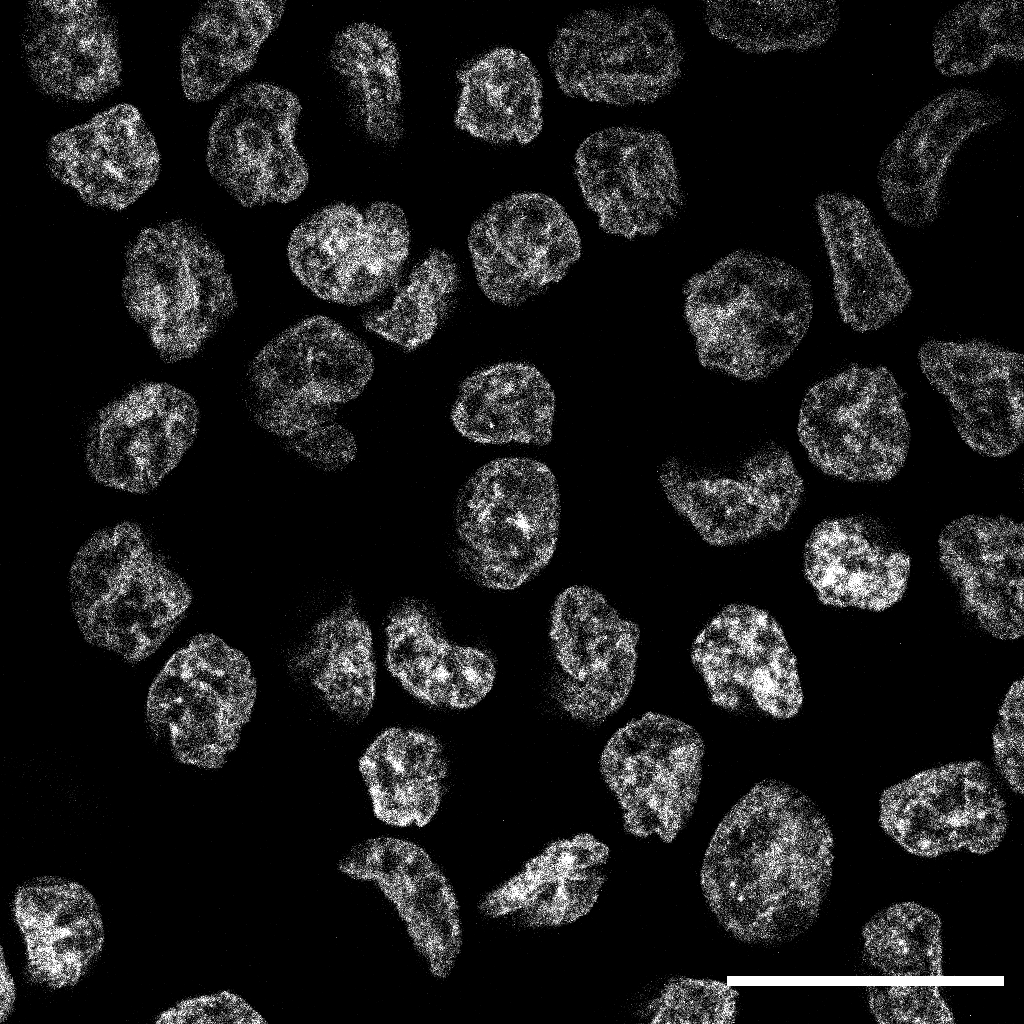

Supplement: Supplementary file 6 — Source data Fig. 5 [file 44318_2024_338_MOESM6_ESM.zip › SD figure 5/5B/FBS_DAPI.png]

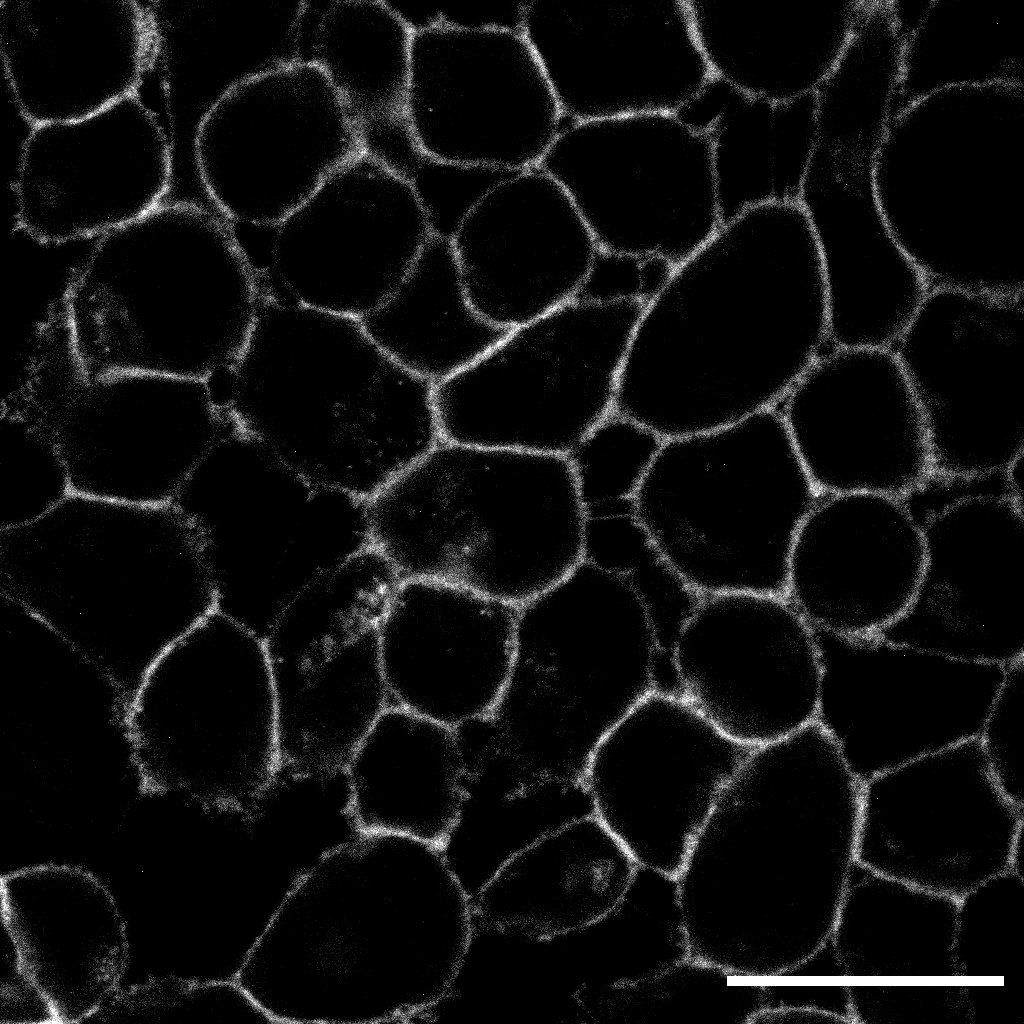

Supplement: Supplementary file 6 — Source data Fig. 5 [file 44318_2024_338_MOESM6_ESM.zip › SD figure 5/5B/FBS_CAD.png]

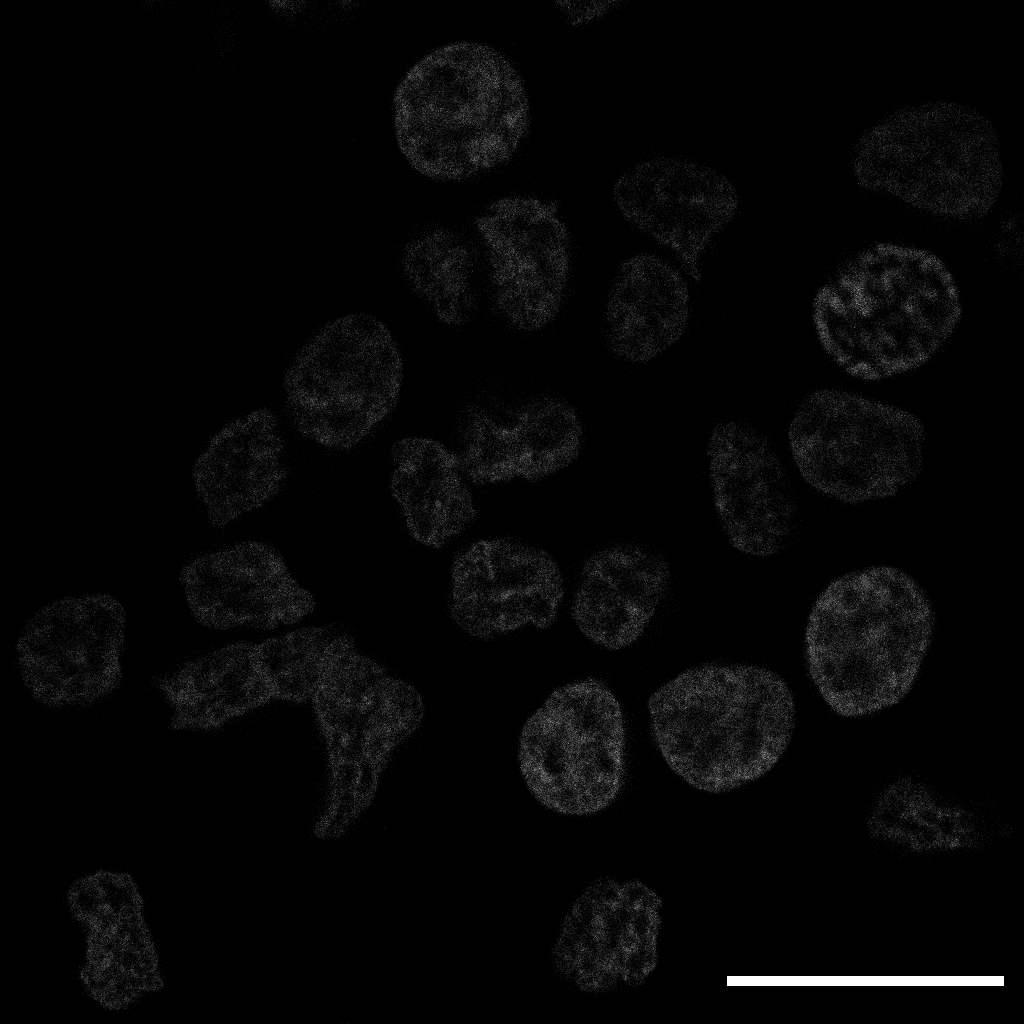

Supplement: Supplementary file 6 — Source data Fig. 5 [file 44318_2024_338_MOESM6_ESM.zip › SD figure 5/5B/SS_GCN2iB_HGF_DAPI.png]

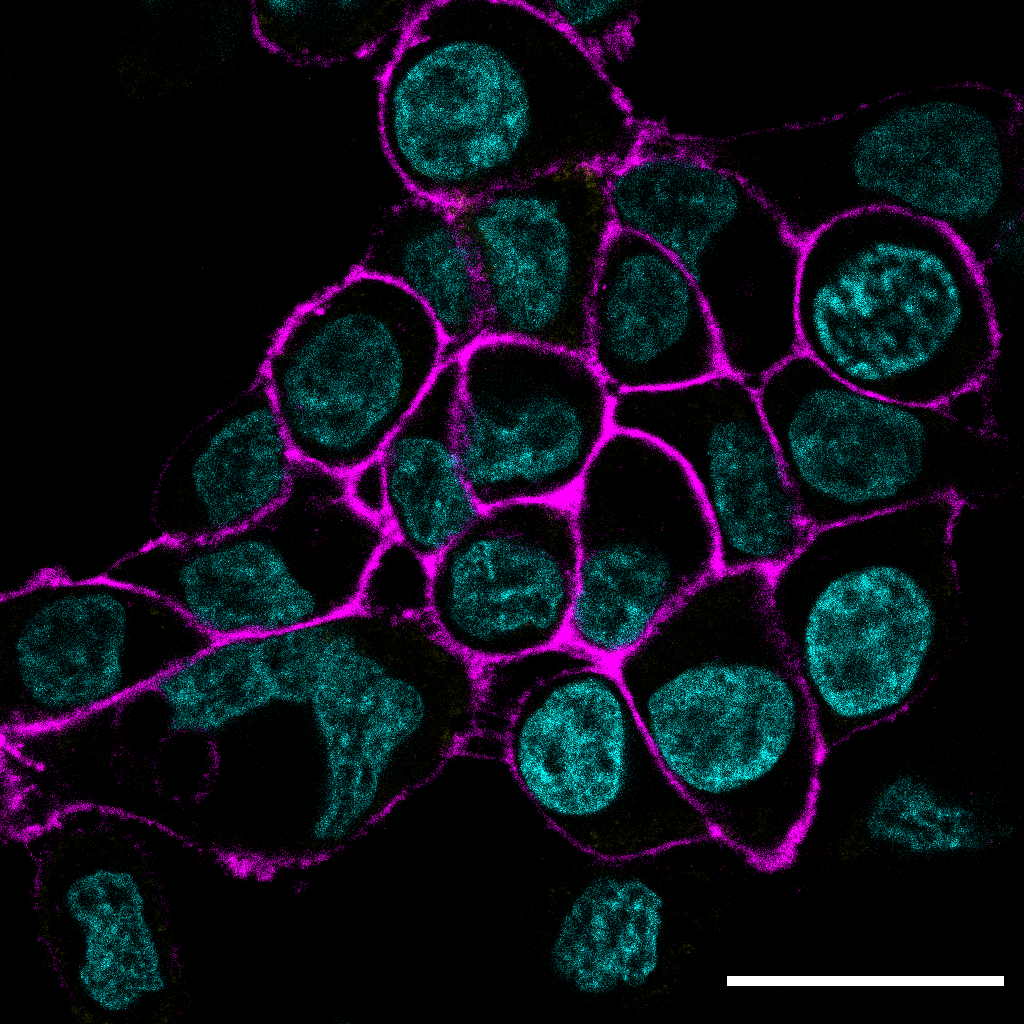

Supplement: Supplementary file 6 — Source data Fig. 5 [file 44318_2024_338_MOESM6_ESM.zip › SD figure 5/5B/SS_GCN2iB_HGF_Merge.png]

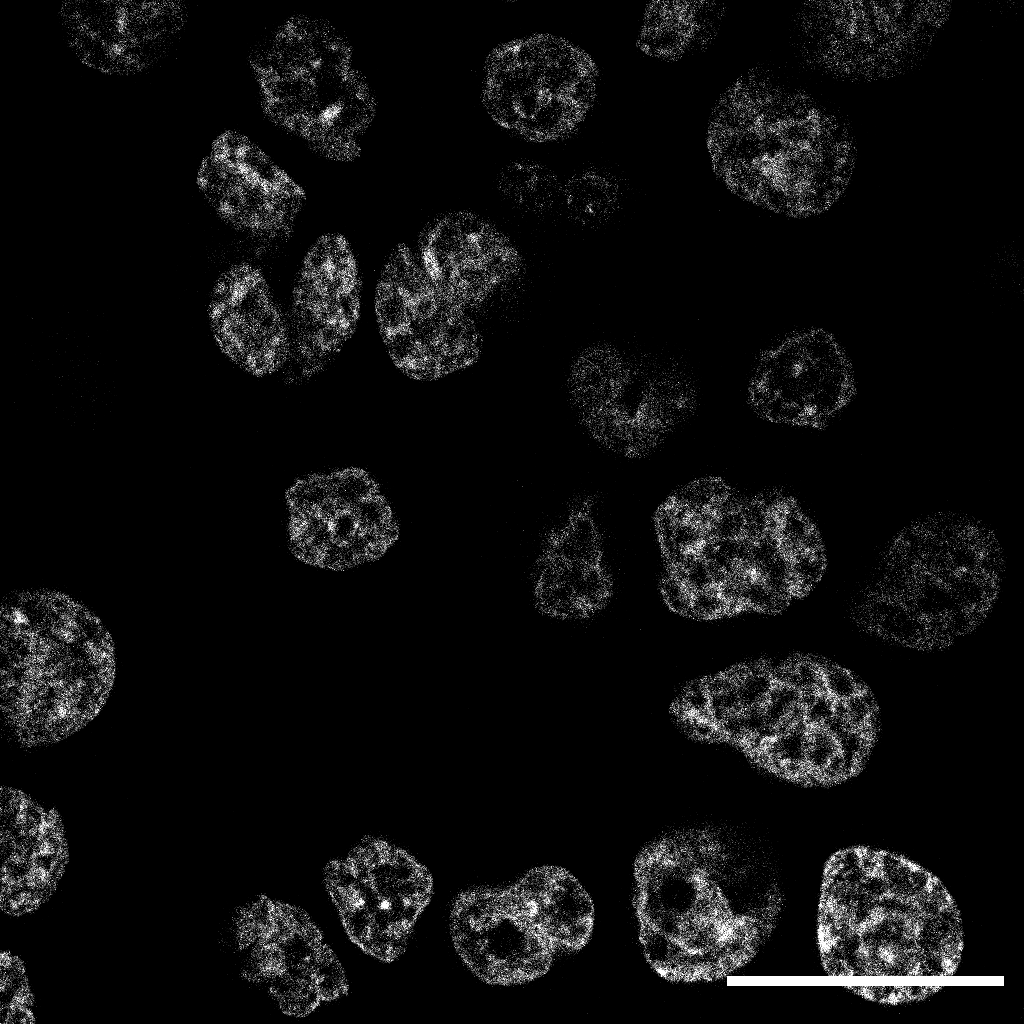

Supplement: Supplementary file 6 — Source data Fig. 5 [file 44318_2024_338_MOESM6_ESM.zip › SD figure 5/5B/FBS_HGF_DAPI.png]

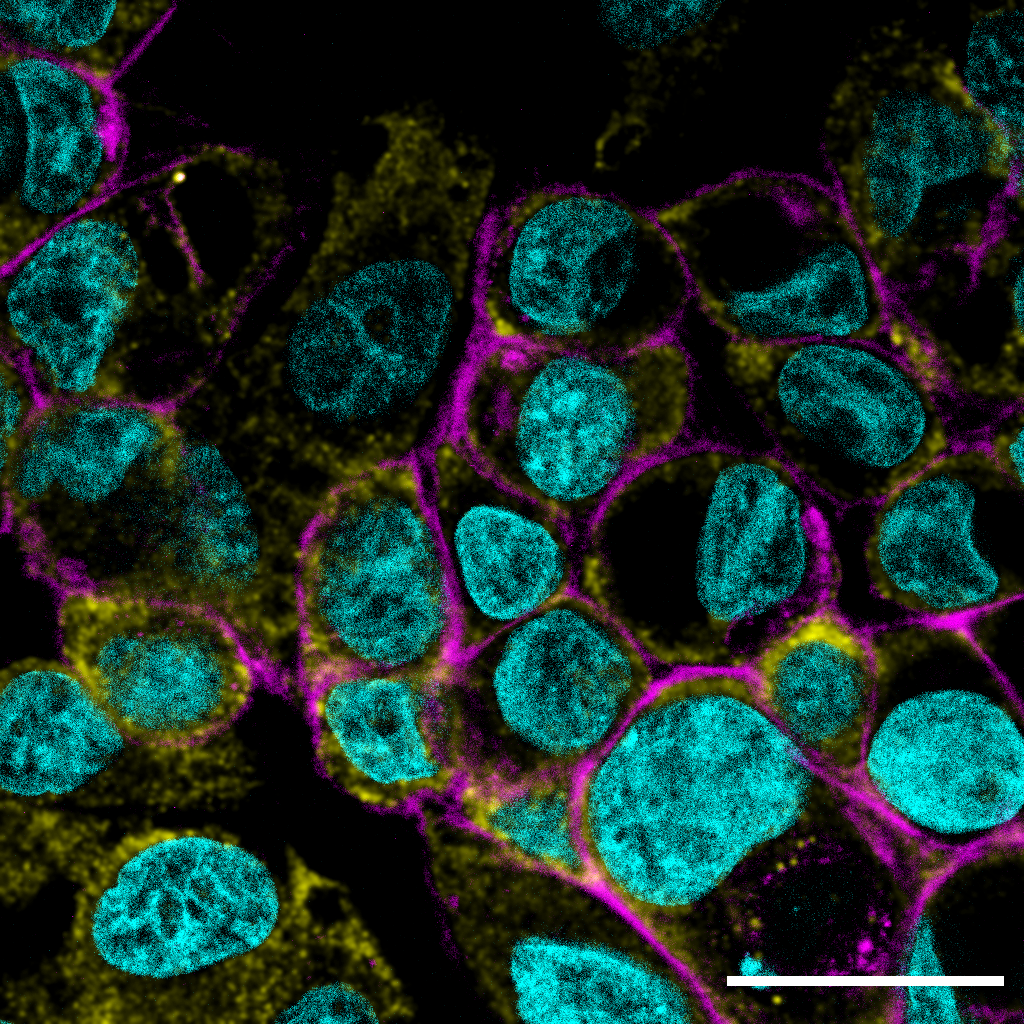

Supplement: Supplementary file 6 — Source data Fig. 5 [file 44318_2024_338_MOESM6_ESM.zip › SD figure 5/5B/SS_HGF__Merge.png]

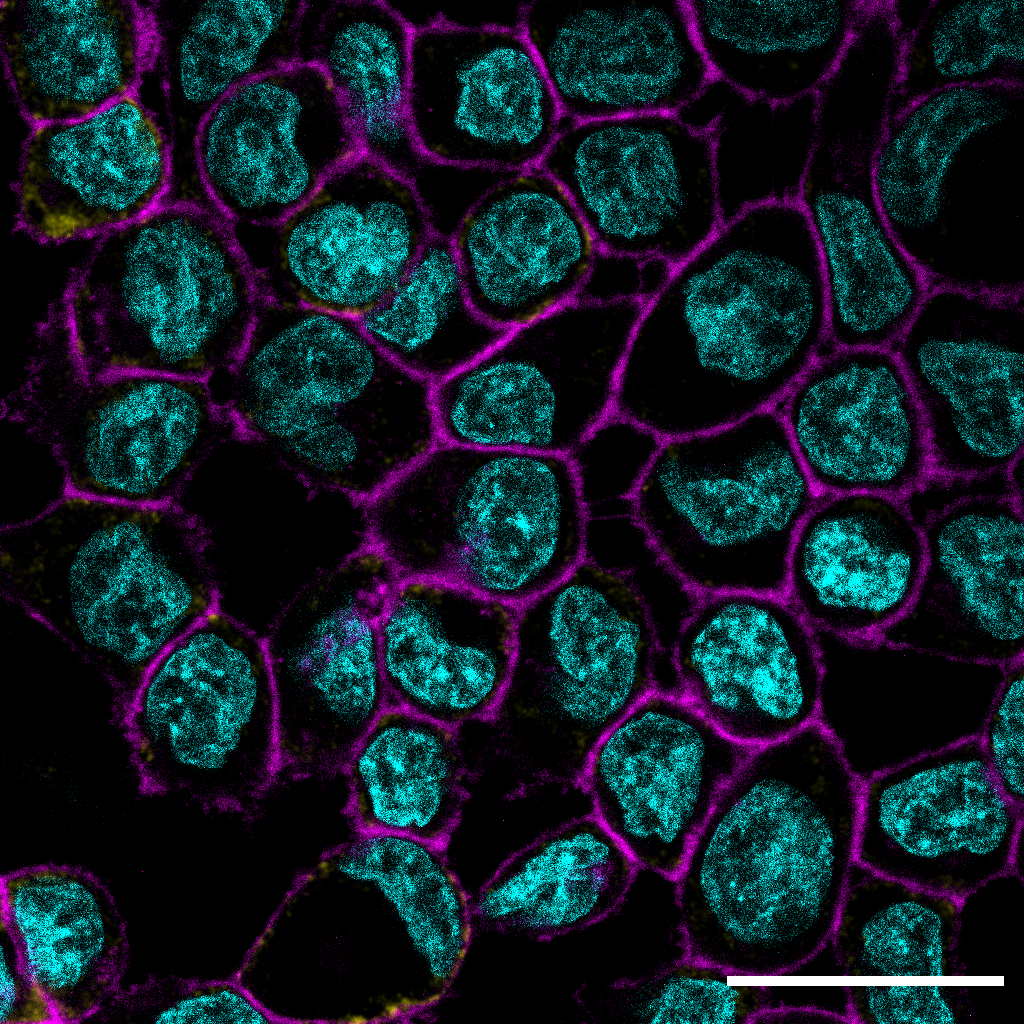

Supplement: Supplementary file 6 — Source data Fig. 5 [file 44318_2024_338_MOESM6_ESM.zip › SD figure 5/5B/FBS_Merge.png]

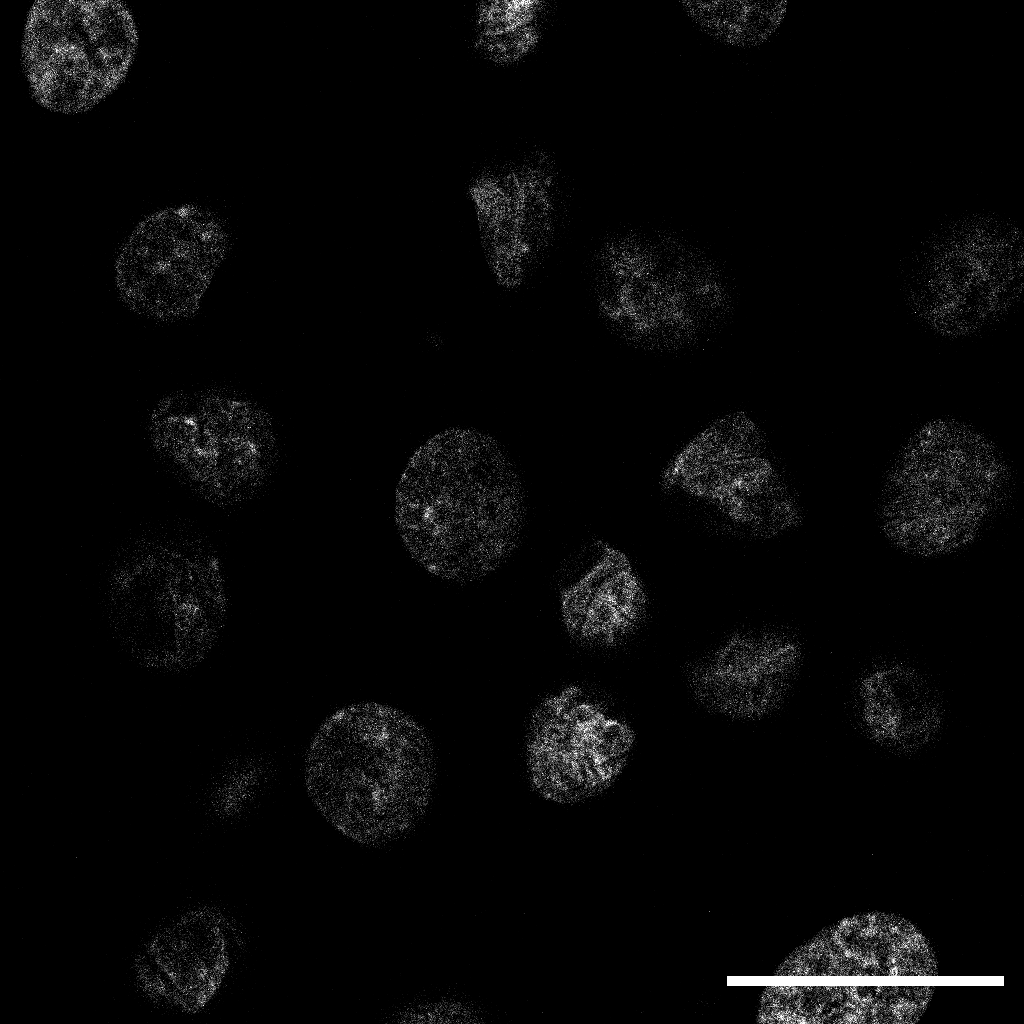

Supplement: Supplementary file 6 — Source data Fig. 5 [file 44318_2024_338_MOESM6_ESM.zip › SD figure 5/5B/SS_DAPI.png]

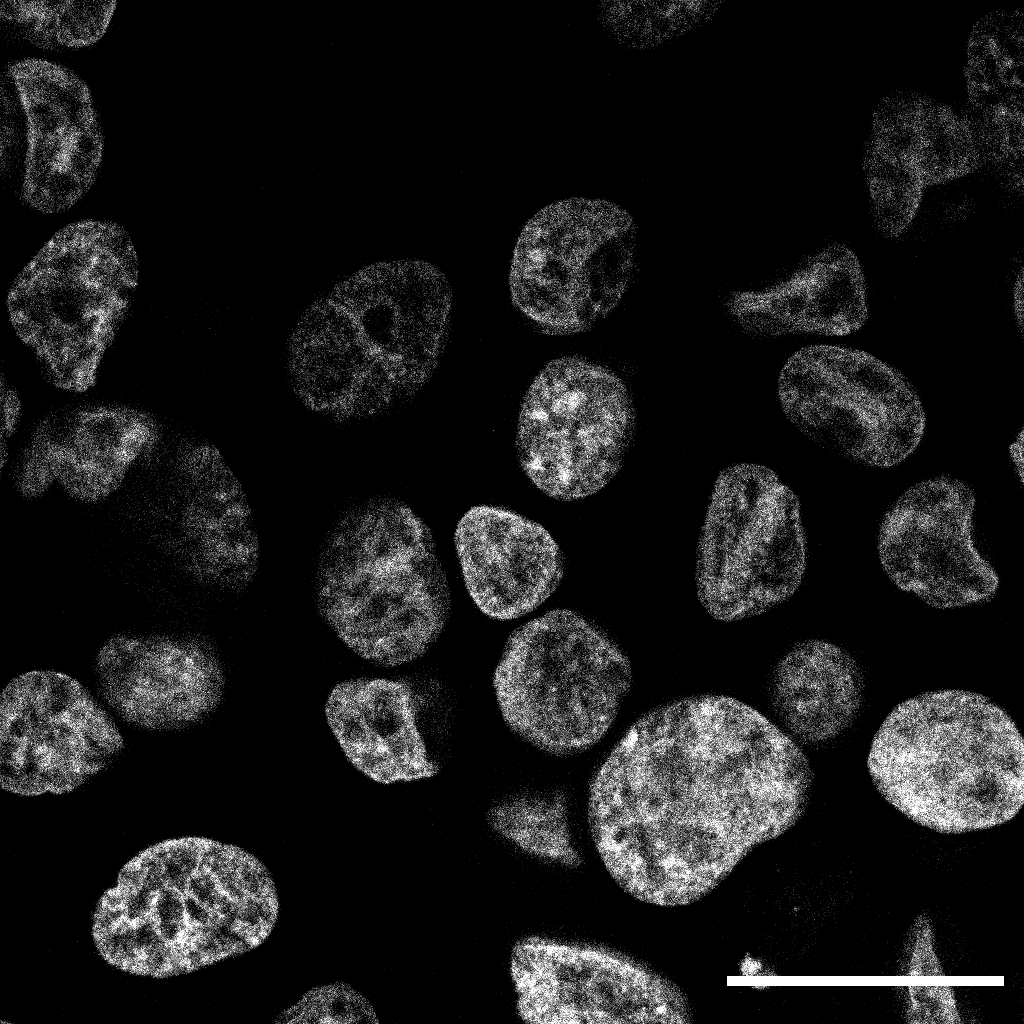

Supplement: Supplementary file 6 — Source data Fig. 5 [file 44318_2024_338_MOESM6_ESM.zip › SD figure 5/5B/SS_HGF_DAPI.png]

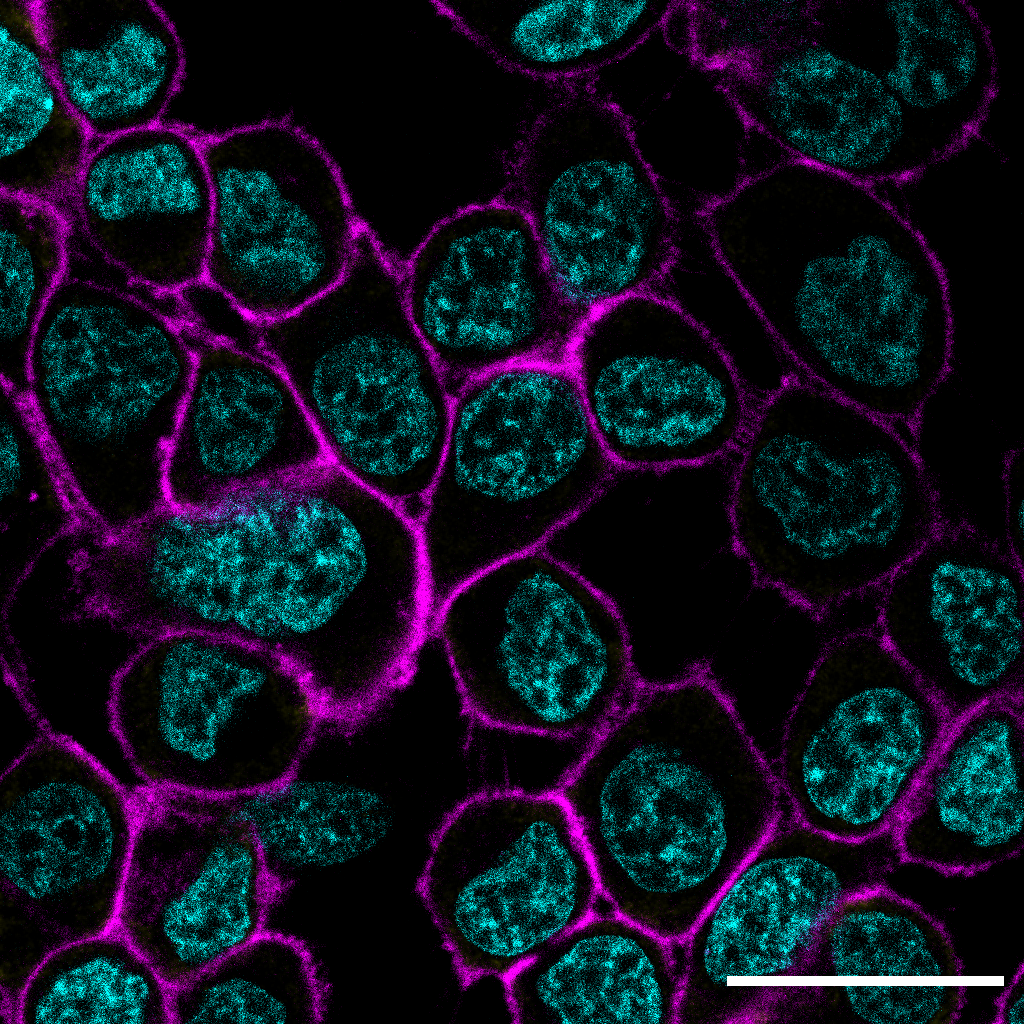

Supplement: Supplementary file 6 — Source data Fig. 5 [file 44318_2024_338_MOESM6_ESM.zip › SD figure 5/5B/SS_GCN2iB_Merge.png]

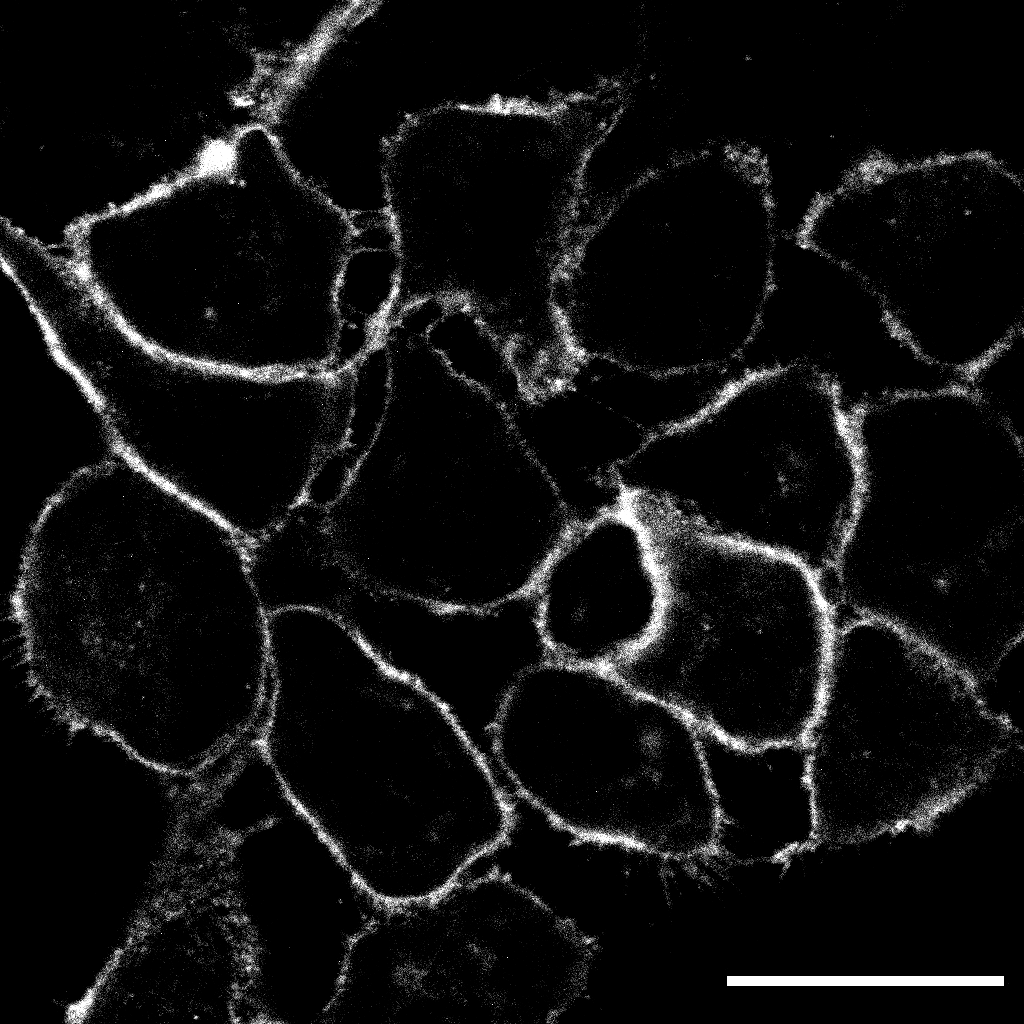

Supplement: Supplementary file 6 — Source data Fig. 5 [file 44318_2024_338_MOESM6_ESM.zip › SD figure 5/5B/SS_CAD.png]

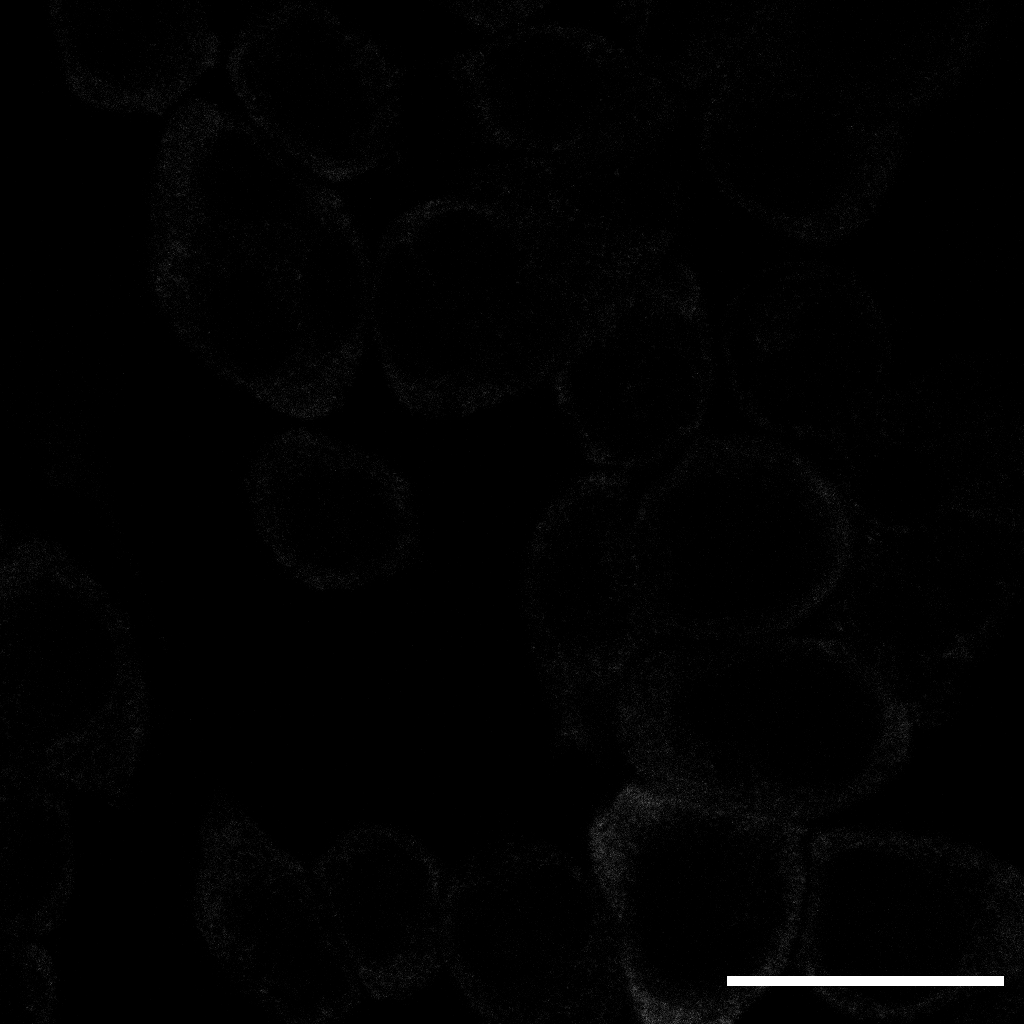

Supplement: Supplementary file 6 — Source data Fig. 5 [file 44318_2024_338_MOESM6_ESM.zip › SD figure 5/5B/FBS_HGF_ACTA2.png]

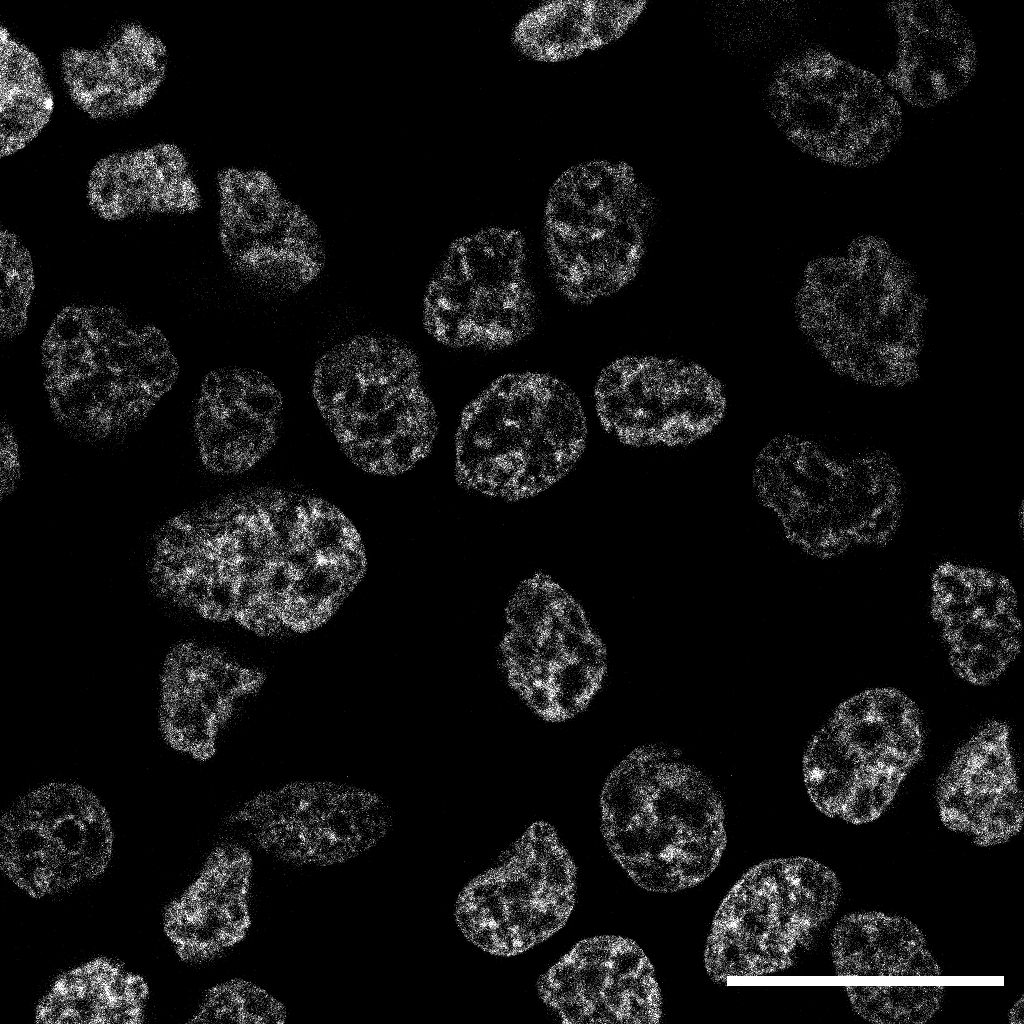

Supplement: Supplementary file 6 — Source data Fig. 5 [file 44318_2024_338_MOESM6_ESM.zip › SD figure 5/5B/SS_GCN2iB_DAPI.png]

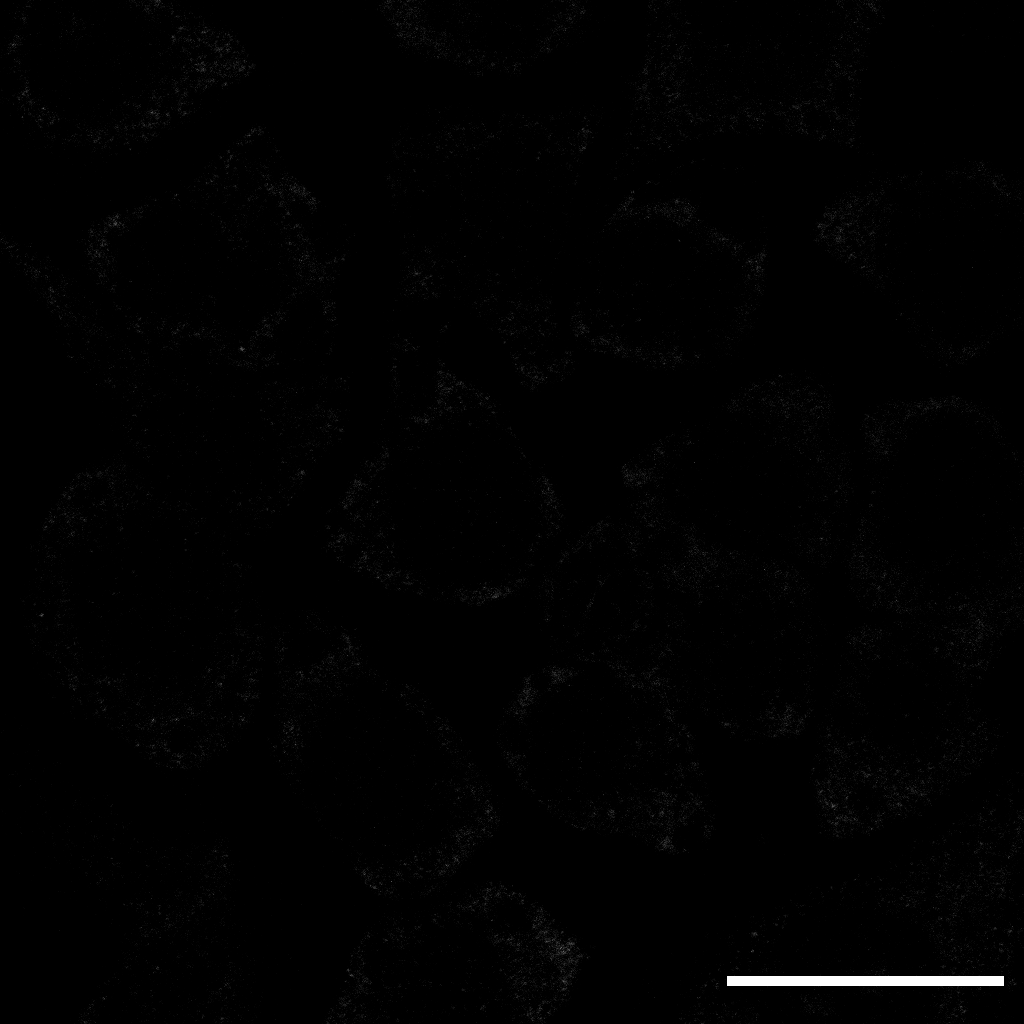

Supplement: Supplementary file 6 — Source data Fig. 5 [file 44318_2024_338_MOESM6_ESM.zip › SD figure 5/5B/SS_ACTA2.png]

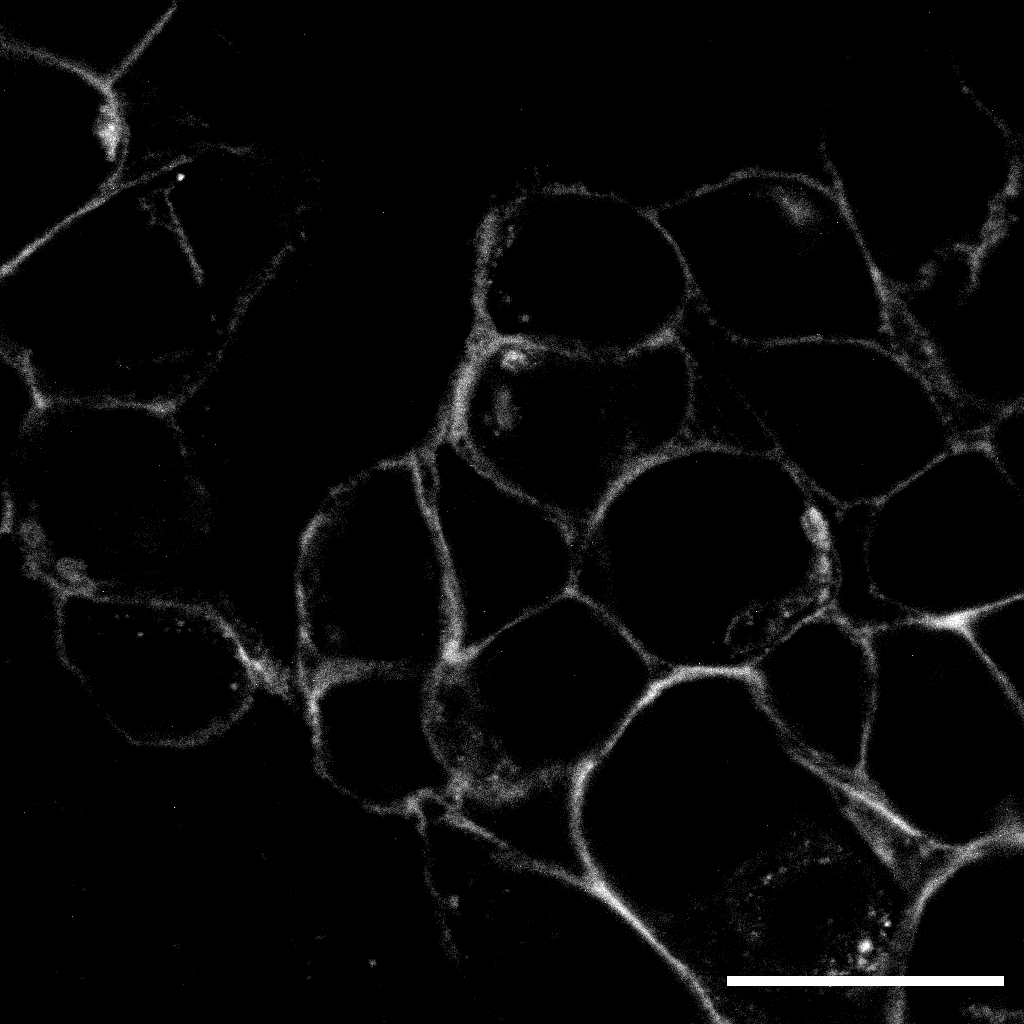

Supplement: Supplementary file 6 — Source data Fig. 5 [file 44318_2024_338_MOESM6_ESM.zip › SD figure 5/5B/SS_HGF_CAD.png]
