# Supplementary material for: The integrated stress response drives MET oncogene overexpression in cancers
Source: EMBO J. 2025 Jan 7;44(4):1107–30. doi: 10.1038/s44318-024-00338-4 (PMC11832788; doi:10.1038/s44318-024-00338-4)
Supplement: Supplementary file 7 — Expanded View Figures [file 44318_2024_338_MOESM7_ESM.pdf]

Expanded View Figures

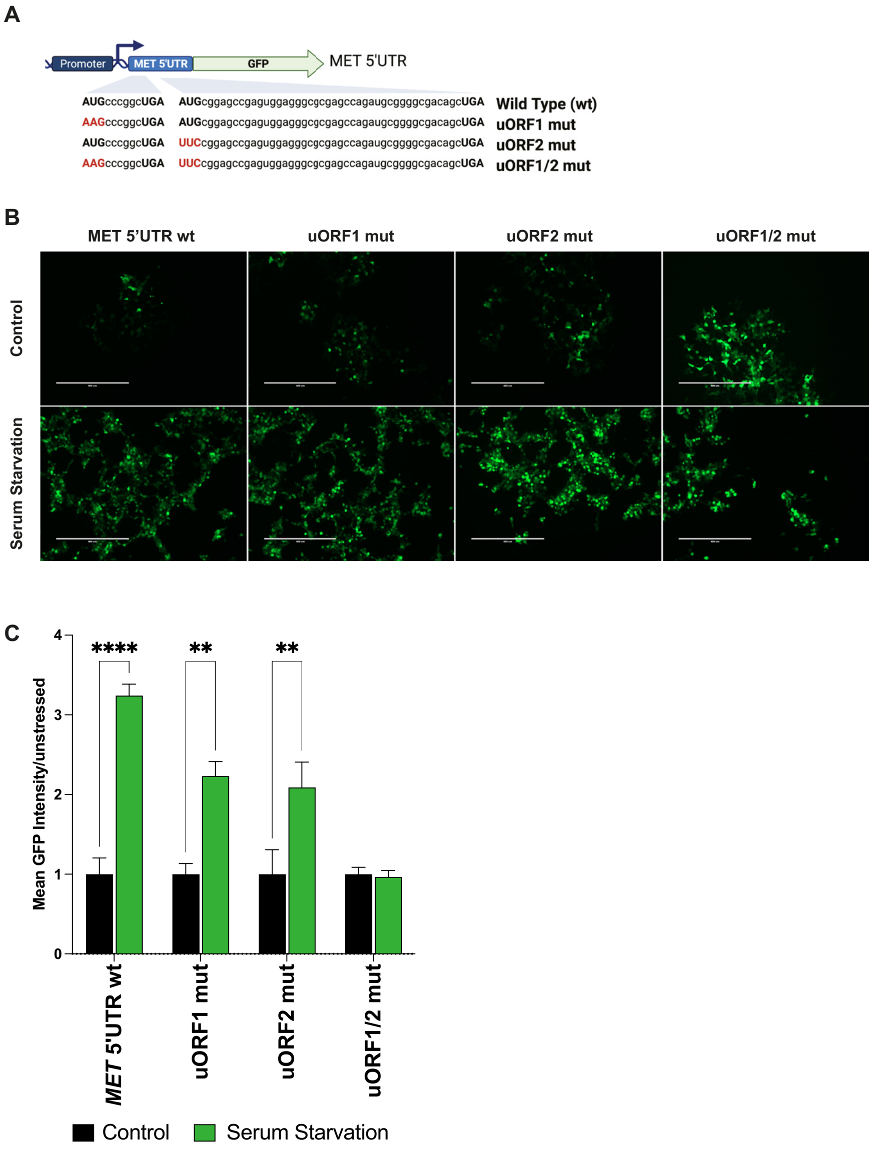

Figure EV1. ***MET*** **mRNA translation is upregulated by a broad range of stress stimuli through the 5′UTR.**

(**A**) Schematic representation of the GFP reporter construct under the control of the MET 5′UTR. Sequences depict wild-type (wt) and mutant (mut) versions of uORF1, uORF2, or the double mutant uORF1/2. (**B**) Fluorescence microscopy images of cells transfected with constructs containing the MET wt 5′UTR or its uORF mutants cultured under control conditions or serum starvation. Scale bars represent 400 µm. (**C**) Quantification of GFP fluorescence intensity from images in (**B**). Data are presented as mean GFP intensity normalised to the control condition for each construct. Results are mean ± SEM ($n = 3$ biological replicates). The P value is calculated using one-way ANOVA with Tukey's post hoc test. Represented P values for control vs. serum starvation wt: 4.47×10^{-5} (****); control vs. serum starvation uORF1 mut: 2.01×10^{-3} (**); control vs. serum starvation uORF2 mut: 5.80×10^{-3} (**); control vs. serum starvation uORF1/2 mut: 0.995 (non-significant).

© The Author(s)

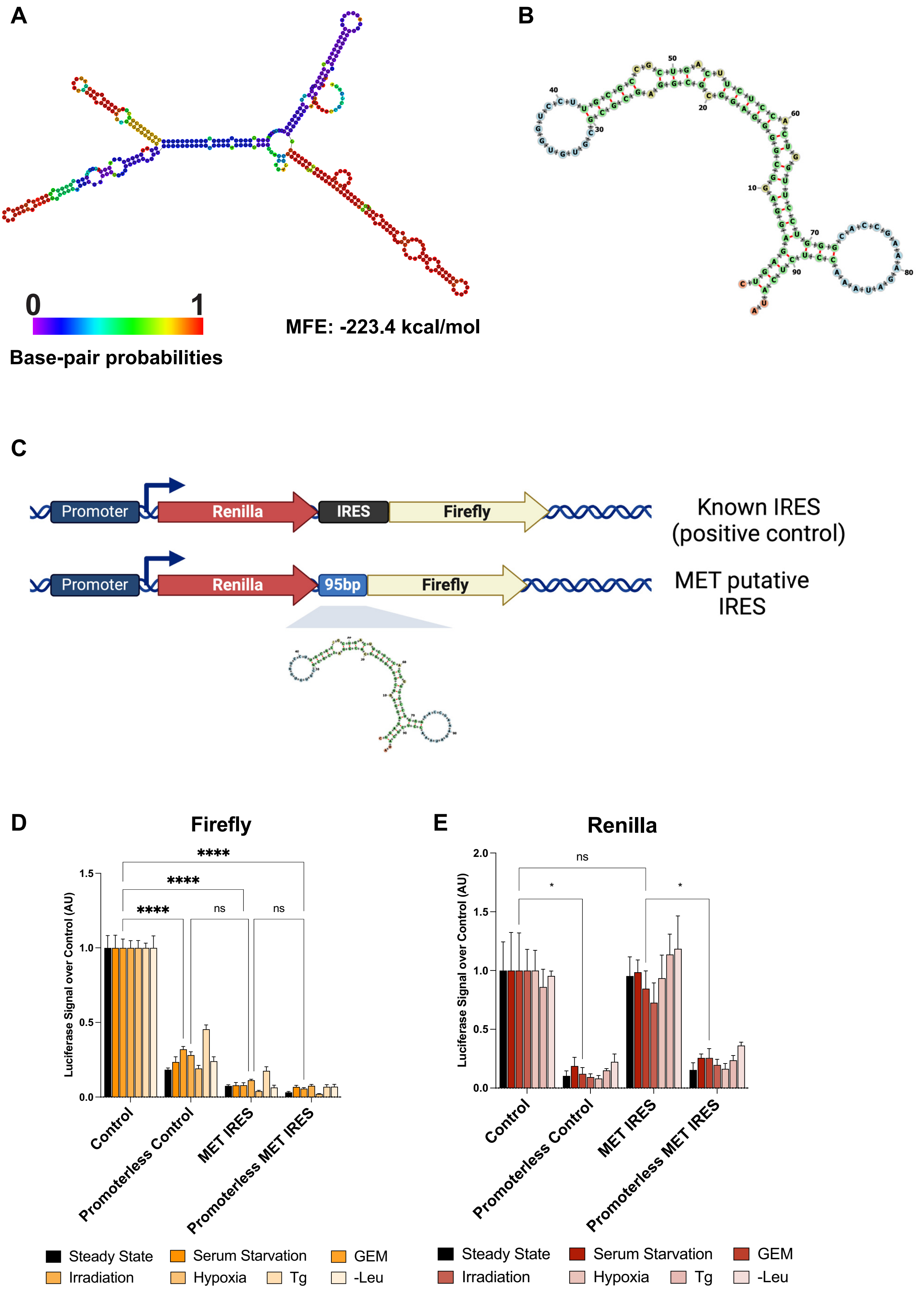
A
0
1
Base-pair probabilities
MFE: -223.4 kcal/mol
B
C
Promoter
Renilla
IRES
Firefly
Known IRES (positive control)
Promoter
Renilla
95bp
Firefly
MET putative IRES
D
Firefly
Luciferase Signal over Control (AU)
****
****
****
ns
ns
Control
Promoterless Control
MET IRES
Promoterless MET IRES
Steady State
Serum Starvation
GEM
Irradiation
Hypoxia
Tg
-Leu
E
Renilla
Luciferase Signal over Control (AU)
ns
*
*
Control
Promoterless Control
MET IRES
Promoterless MET IRES
Steady State
Serum Starvation
GEM
Irradiation
Hypoxia
Tg
-Leu

 © The Author(s)

Figure EV2. Structural prediction of *MET* 5′UTR and functional assessment of a putative *MET* IRES.

(**A**) Predicted secondary structure of the *MET* 5′UTR as determined by RNAfold (Lorenz et al, 2011). The structure is colour-coded according to base-pair probabilities, with the minimum free energy (MFE) calculated to be −223.4 kcal/mol. (**B**) Secondary structure of the putative *MET* IRES as described by the human IRES atlas (Yang et al, 2021). The IRES is 95 bases in length, starting at base 302 and ending at base 396. (**C**) Schematic representation of the bicistronic reporter constructs used to assess IRES activity. The predicted *MET* IRES sequence was cloned between the Renilla luciferase (RLuc) and Firefly luciferase (FLuc) genes. A vector containing a known functional IRES sequence was used as a positive control (Poulin et al, 1998). (**D**) Firefly Luciferase measurements assessing the activity of the MET putative IRES in comparison to a known IRES sequence. Promoterless vectors were used as negative controls. The error bars represent mean ± SEM with n = 3 biological replicates. The *P* value is calculated using two-way ANOVA with Tukey's post hoc test and represents the row factor (different constructs) and column factor (different treatments). (**E**) Renilla results reflect promoter-driven expression, showing that the CMV promoter drives the first cistron consistently across conditions, ensuring that the system functions as expected. Results are represented and analysed as described in (**D**). Significance markers: ****$P < 0.0001$, *$P < 0.05$, and "ns" indicates not significant.

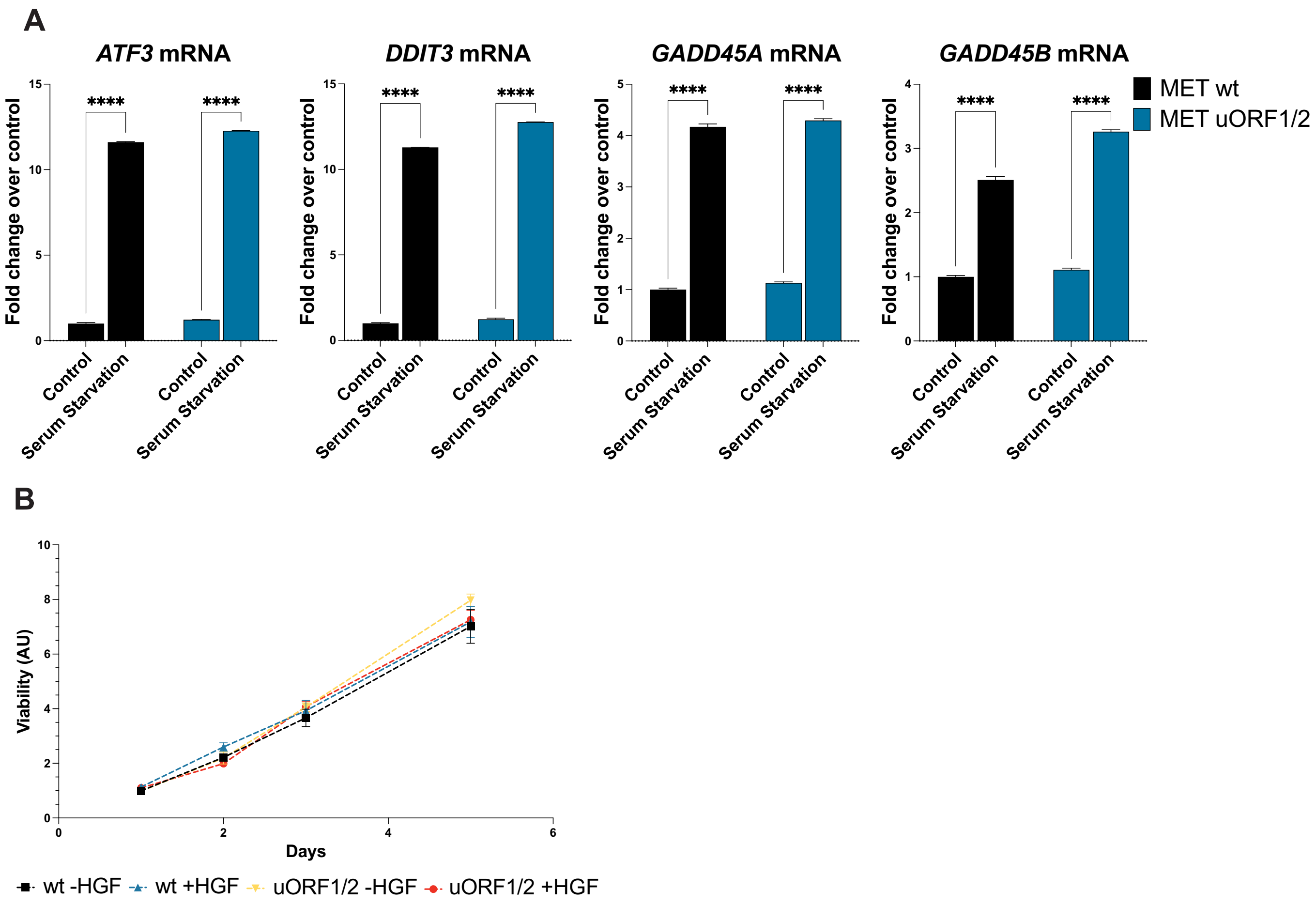

Figure EV3. ***MET*** **translation and the subsequent biological activity are regulated by the two uORFs.**

(**A**) Bar graphs presenting the relative mRNA levels of *ATF3*, *DDIT3*, *GADD45A*, and *GADD45B* in cells expressing either MET wt or MET 5′UTR with uORF1/2 mutations. The cells were subjected to ± serum starvation. Data are normalised to *TBP* expression levels. Results are represented as mean ± SEM ($n = 3$ biological replicates) and analysed by one-way ANOVA with Tukey post hoc test. Exact *P* values: ATF3 (Control vs. Serum Starvation, MET wt: $P = 4.56 \times 10^{-6}$, MET uORF1/2: $P = 1.67 \times 10^{-5}$); DDIT3 (Control vs. Serum Starvation, MET wt: $P = 2.09 \times 10^{-5}$, MET uORF1/2: $P = 3.98 \times 10^{-5}$); GADD45A (Control vs. Serum Starvation, MET wt: $P = 2.11 \times 10^{-5}$, MET uORF1/2: $P = 1.12 \times 10^{-5}$); GADD45B (Control vs. Serum Starvation, MET wt: $P = 1.33 \times 10^{-5}$, MET uORF1/2: $P = 9.08 \times 10^{-5}$). (**B**) Viability assay showing the growth curves of cells over six days under control conditions, comparing wild-type with uORF1/2 double-mutant cells ± HGF. Two-way ANOVA with repeated measures was used to analyse data ($n > 3$ biological replicates). Significance marker: ****$P < 0.0001$.

 © The Author(s)

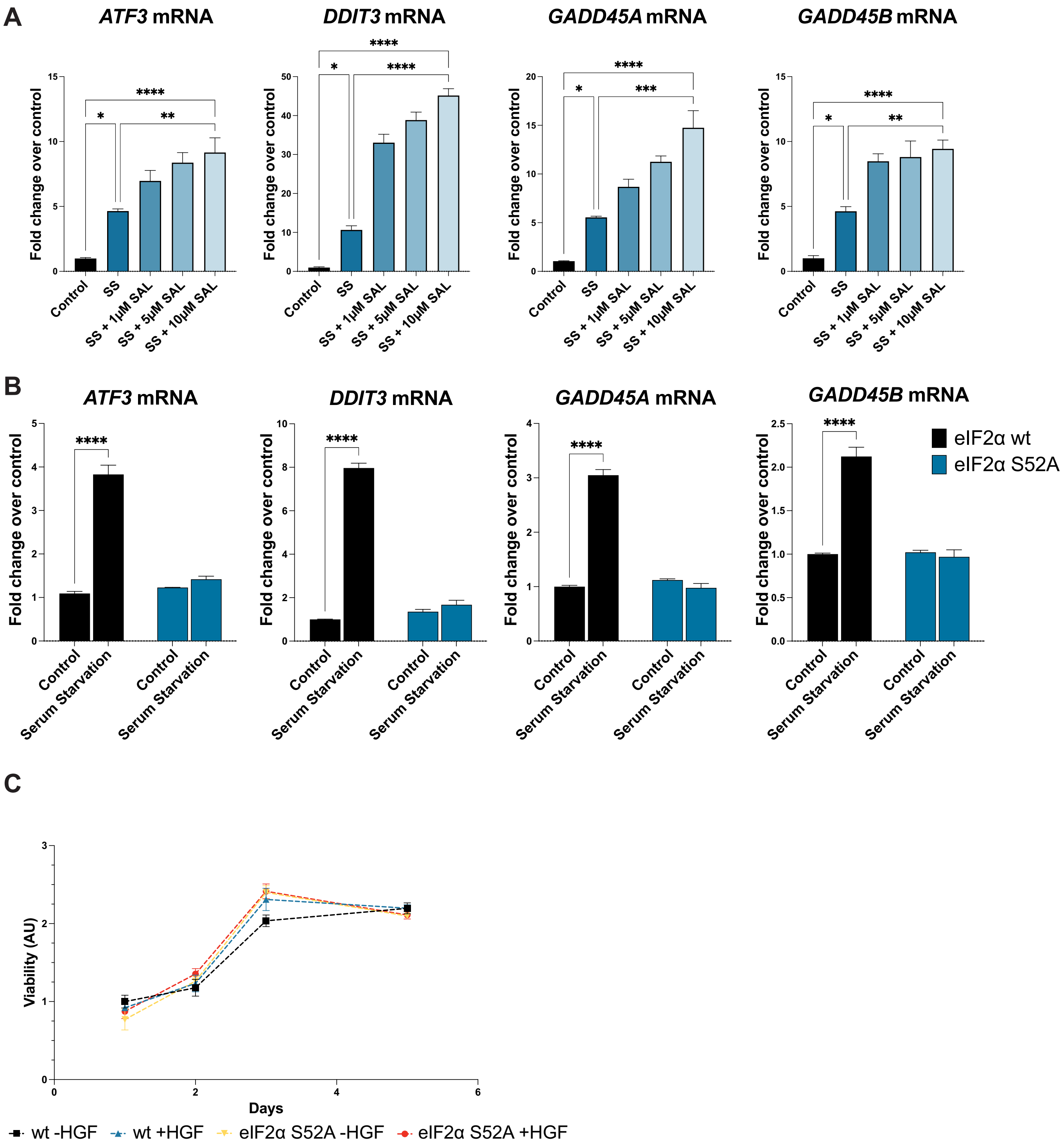
A
ATF3 mRNA
DDIT3 mRNA
GADD45A mRNA
GADD45B mRNA
Fold change over control
Control
SS
SS + 1μM SAL
SS + 5μM SAL
SS + 10μM SAL
*
**
***
****
B
ATF3 mRNA
DDIT3 mRNA
GADD45A mRNA
GADD45B mRNA
Fold change over control
Control
Serum Starvation
****
eIF2α wt
eIF2α S52A
C
Viability (AU)
Days
wt -HGF
wt +HGF
eIF2α S52A -HGF
eIF2α S52A +HGF

© The Author(s)

Figure EV4. MET expression and the ensuing biological activity are controlled by the *integrated stress response* regulator eIF2α.

(**A**) RT-qPCR results showing the expression of ATF4 target genes (*ATF3*, *DDIT3*, *GADD45A*, and *GADD45B)* in EKVX cells treated with ± SS ± increasing amount of Salubrinal for 24 h. Data are represented as mean ± SEM with $n = 3$ biological triplicates and were analysed using one-way ANOVA with Tukey's post hoc test. (**B**) Bar graphs presenting the relative mRNA levels of *ATF3*, *DDIT3*, *GADD45A*, and *GADD45B* in cells expressing either MET wt or MET 5'UTR with eIF2*α* S52A mutation. The cells were subjected to ± serum starvation. Data are normalised to *TBP* expression levels and are represented as mean ± SEM ($n = 3$ biological replicates), and data are analysed by one-way ANOVA with post hoc tests comparing the effects of serum starvation on gene expression. Exact p values: ATF3 (Control vs. Serum Starvation, eIF2α wt: $P = 5.01 \times 10^{-5}$); DDIT3 (Control vs. Serum Starvation, eIF2α wt: $P = 3.02 \times 10^{-5}$); GADD45A (Control vs. Serum Starvation, eIF2α wt: $P = 2.16 \times 10^{-5}$); GADD45B (Control vs. Serum Starvation, eIF2α wt: $P = 9.31 \times 10^{-5}$). (**C**) Viability assay showing the growth curves of cells over six days under control conditions ±HGF, comparing wild-type with eIF2*α* S52A mutation. Data are represented as mean ± SEM with $n > 3$ biological replicates. Statistical test was performed using two-way ANOVA with repeated measures (non-significant). Significance marker: ****$P < 0.0001$, ***$P < 0.001$, **$P < 0.01$, and *$P < 0.05$.

© The Author(s)

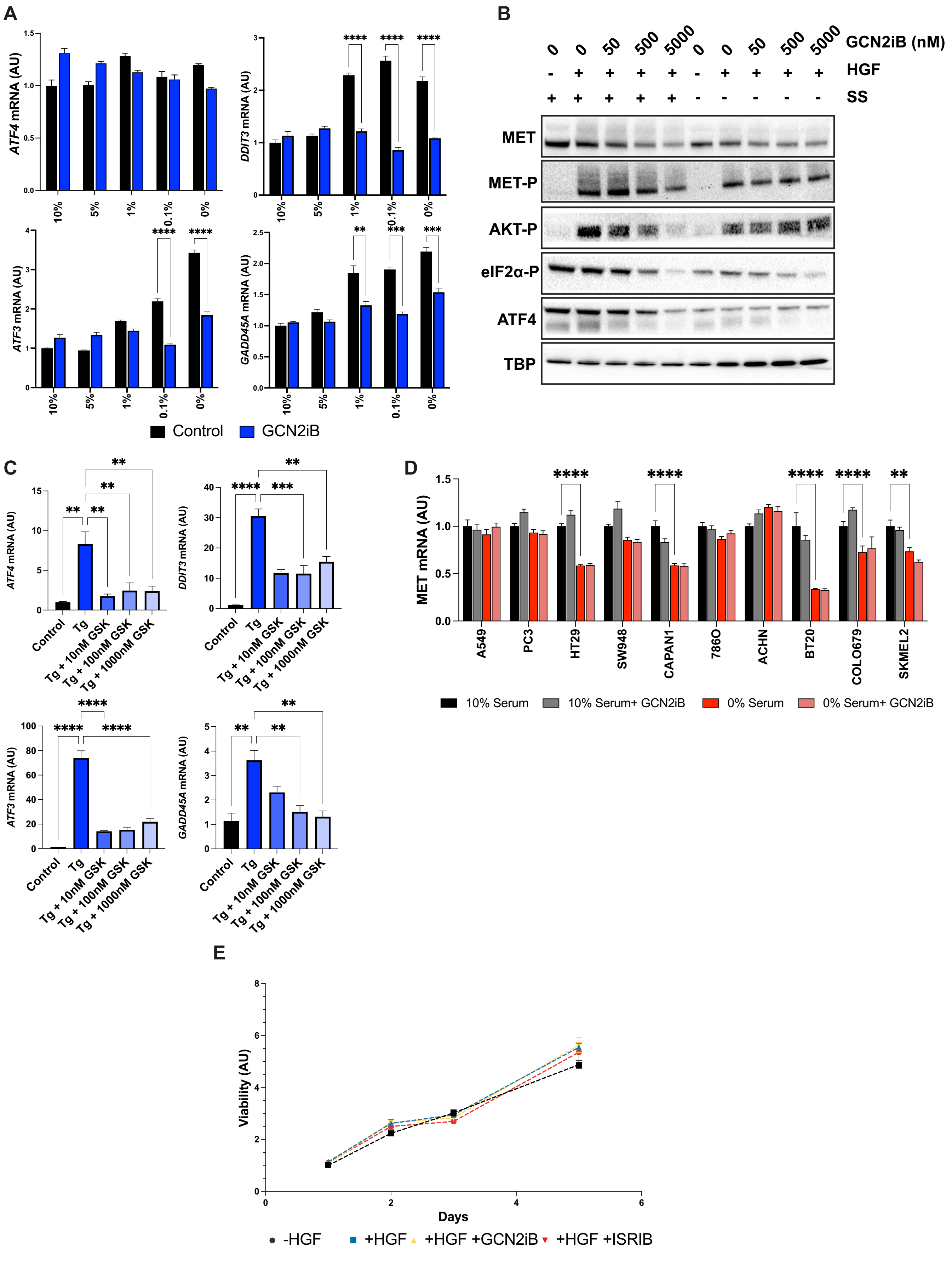

A
ATF4 mRNA (AU)
DDIT3 mRNA (AU)
ATF3 mRNA (AU)
GADD45A mRNA (AU)
10%
5%
1%
0.1%
0%
Control
GCN2iB
B
0
50
500
5000
GCN2iB (nM)
HGF
SS
MET
MET-P
AKT-P
eIF2α-P
ATF4
TBP
C
Control
Tg
Tg + 10nM GSK
Tg + 100nM GSK
Tg + 1000nM GSK
D
MET mRNA (AU)
A549
PC3
HT29
SW948
CAPAN1
786O
ACHN
BT20
COLO679
SKMEL2
10% Serum
10% Serum+ GCN2iB
0% Serum
0% Serum+ GCN2iB
E
Viability (AU)
Days
-HGF
+HGF
+HGF +GCN2iB
+HGF +ISRIB

Figure EV5. Stress-induced MET upregulation is impinged by ISR inhibition.

(**A**) RT-qPCR analysis of *ATF4*, *DDIT3*, *ATF3*, and *GADD45A* in EKVX cells under control condition (10% serum) or decreasing amount of serum (from 5% to 0%) ± 500 nM GCN2iB. The graphs depict fold changes over control with *TBP* as a reference gene. Error bars represent SEM with $n = 3$ biological replicates. Data were analysed using two-way ANOVA. (**B**) Cells were cultured in 10% serum (control) or under serum starvation ± increasing concentrations of GCN2iB for 24 h and treated with ± 50 ng/mL of HGF for 15 min. Western blot panels showing MET, MET-P, AKT-P, eIF2α-P, and ATF4 levels, with TBP as the loading control. (**C**) RT-qPCR analysis of *ATF4*, *DDIT3*, *ATF3*, and *GADD45A* mRNA levels in EKVX cells treated with 500 nM Tg for 24 h in the presence or absence of the PERK inhibitor GSK2656157 (GSK, 10 nM, 100 nM, or 1000 nM). mRNA levels were normalised to *TBP*, and results are presented as fold changes over control conditions. Error bars represent SEM with $n = 3$ biological replicates. Data were analysed by one-way ANOVA with Tukey's post hoc test. (**D**) RT-qPCR experiments showing *MET* mRNA expression across a panel of cell lines under control condition (10% serum) or serum starvation (0% serum) ± 500 nM GCN2iB for 24 h. Results are fold change over control condition normalised with *TBP*. Results are presented as mean ± SEM with $n = 3$ biological replicate and analysed using two-way ANOVA. (**E**) The viability of EKVX cells is quantified over six days under control condition (10% Serum) ± HGF (50 ng/mL) ± ISR inhibitors GCN2iB or ISRIB. Error bars represent SEM with $n > 3$ biological replicates. Two-way ANOVA with repeated measures was used to evaluate significance (non-significant). Significance is denoted as ****$P < 0.0001$, ***$P < 0.001$, and **$P < 0.01$.

© The Author(s)
